# Effects of aerobic exercise intervention on the association between brain functional connectivity and cognition among older adults

**DOI:** 10.1016/j.nbas.2026.100162

**Published:** 2026-07-28

**Authors:** Myungjin Jung, Marco Pipoly, Bryan Madero, Chris Oehler, Kelsey Baller, Vincent A. Magnotta, Jeffrey D. Long, Gary L. Pierce, Michelle W. Voss

**Affiliations:** a Department of Kinesiology, Health Promotion and Recreation, University of North Texas, Denton, TX 76201, USA; b Department of Psychological and Brain Sciences, The University of Iowa, Iowa City, IA 52242, USA; c Center for Vital Longevity, University of Texas at Dallas, Dallas, TX 75235, USA; d Department of Radiology, The University of Iowa, Iowa City, IA 52242, USA; e Department of Psychiatry, The University of Iowa, Iowa City, IA 52242, USA; f Department of Biostatistics, The University of Iowa, Iowa City, IA 52242, USA; g Department of Health and Human Physiology, The University of Iowa, Iowa City, IA 52242, USA; h Interdisciplinary Graduate Program in Neuroscience, The University of Iowa, Iowa City, IA 52242, USA; i Iowa Neuroscience Institute, The University of Iowa, Iowa City, IA 52242, USA

**Keywords:** Cognitive performance, Neural network, Physical activity, Resting state fMRI, Time-varying effect modeling

## Abstract

Chronic aerobic exercise is known to protect against brain and cognitive aging in animals, but it remains unclear whether these benefits are consistent across the span of older adulthood. Using time-varying effect modeling (TVEM) analysis, this study investigated how the associations between changes in cognition and brain network functional connectivity (FC), induced by a 6-month aerobic exercise intervention, vary with age among older adults aged 55 to 80 (*n* = 107). Participants were divided into two groups based on exercise intensity: moderate-to-vigorous (ModVig group) and light-intensity (Light group). Before and after the intervention, participants completed cognitive tasks measuring executive function (EF) and processing speed (PS) and underwent resting-state fMRI sessions. FC was assessed across large-scale brain networks, including the frontoparietal control (FPCN), default mode (DMN), dorsal attention (DAN), limbic (LN), salience (SN), somatomotor (SMN), and visual network (VN). Change scores for each cognitive task and FC network were calculated by subtracting pre-intervention scores from post-intervention scores. Older adults in the ModVig group demonstrated improved PS performance at ages 58 to 65 and increased FC at ages 67 to 69 in the DAN. When individuals were exposed to ModVig intensity exercise, increases in FC within the FPCN, DMN, and SN were associated with improved EF and PS performance in those in their mid-60s to mid-70s. These findings support the importance of targeting higher moderate-to-vigorous aerobic exercise intensity for interventions, particularly in this age group, to enhance brain and cognitive health.

## 1 Introduction

Aging increases the risk of cognitive decline and Alzheimer's disease-related dementias [1], [2]. Declines in executive function (EF) and processing speed (PS), which are cognitive abilities essential for maintaining independence, often emerge by midlife and are partly driven by age-related deterioration in brain network function [3], [4]. Early changes in the brain, such as diminished brain network function and reduced modular organization (i.e., the degree to which brain networks preserve distinct communities that enable efficient information processing), are considered indicators of risk for mild cognitive impairment and Alzheimer's disease. These declines reflect neurodegenerative processes of aging, including amyloid-β accumulation, tau pathology, cerebrovascular dysfunction, and white matter deterioration [5], [6], [7]. Identifying sensitive time windows to counteract cognitive and brain health decline is therefore essential for sustaining quality of life in aging populations. Importantly, evidence shows that adopting an active lifestyle, especially regular physical activity, represents a promising preventive strategy against cognitive decline. Erickson et al. [8] reported that physical activity was associated with benefits across the lifespan, with consistent neural biomarker improvements at all ages, more robust brain effects in children and adults over 50, and less consistent cognitive benefits in younger adults.

Aerobic exercise is a promising strategy for mitigating the adverse effects of aging on brain network function and its associated impact on cognition [9]. The benefits of exercise on brain network function are often evaluated using functional magnetic resonance imaging (fMRI), a non-invasive tool that provides a proxy measure of neuronal activity. By correlating fMRI signals across brain regions, researchers can assess the strength of pairwise communication within and between functional brain networks [10]. Recognizing functional connectivity (FC) as a critical biomarker of brain functionality, numerous neuroimaging studies have examined the effects of aerobic exercise training and cardiorespiratory fitness (CRF), defined as the integrated ability of the cardiopulmonary system to deliver oxygen-rich blood and of peripheral tissues to effectively utilize that oxygen through adaptations such as increased blood volume and enhanced oxidative enzyme activity, on FC in older adults. Studies consistently report that aerobic exercise improves CRF, which in turn demonstrates beneficial effects on the health and integrity of functional brain networks [11], [12], [13], [14]. Moreover, as Won et al. [15] discuss, among older adults aged 60 years and older, improvements in CRF through aerobic exercise interventions lead to enhanced cognitive performance, which may be mediated by increases in FC within brain networks. Collectively, regular aerobic exercise that enhances CRF appears to be a key factor in promoting both FC and cognition among older adults [16].

Many brain networks have been studied in relation to physical activity behavior and CRF, and evidence suggests that these factors can make independent contributions to FC. For example, Voss et al. [14] reported that physical activity levels and CRF each explained unique variance in FC, underscoring the importance of distinguishing between behavioral and physiological influences. The default mode network (DMN) and salience network (SN) are frequently highlighted as being particularly responsive to aerobic exercise interventions [17], [18]. Cross-sectional studies of older adults aged 55–80 years found that those regularly engaged in aerobic exercise exhibited higher FC within the DMN [12], [17], which was associated with better performance on EF tasks [15]. Similarly, a 12-week treadmill walking intervention by Won et al. [18] reported training-induced increases in FC within the SN among healthy older adults with a mean age of 78 years, and these increases were positively correlated with improved immediate recall of logical memory, reflecting enhanced working memory processes. In contrast, other studies have reported mixed results. Chirles et al. [19] observed a decrease in FC within the DMN among older adults aged 60–88 years, while McFadden et al. [20] found no significant changes in FC of the SN in obese young to middle-aged adults with a mean age of 38 years. Won et al. [18] similarly reported no changes in FC within the frontoparietal control network (FPCN) in older adults with a mean age of 78 years, whereas Dorsman et al. [21] observed significant increases in FPCN FC among older adults with an average age of 73 years.

Such discrepancies in findings may arise from several factors that moderate these effects, including differences in exercise protocols, participant characteristics, and baseline CRF levels. In particular, exercise intensity may be an important determinant of exercise-related cognitive and neural adaptations in older adults. More vigorous aerobic activity is thought to induce greater improvements in CRF, cerebrovascular function, and neurotrophic signaling compared to lower-intensity activity, which may contribute to stronger effects on cognition and FC [16]. Additionally, variability in how brain networks are defined and measured presents a critical challenge in exercise neuroimaging studies. Inconsistent operational definitions of networks complicate comparisons and interpretations across studies. Most exercise neuroimaging research have predominantly focused on cortical networks that support higher-order cognition, while exercise-related changes in motor and sensory networks have received comparatively less attention. To address this gap, our study assessed large-scale network connectivity using Yeo's seven-network framework [22]. This model includes the FPCN, DMN, dorsal attention (DAN), limbic (LN), SN, somatomotor (SMN), and visual (VN).^1^ These networks represent replicable and widely referenced brain systems that play distinct roles across diverse functional domains, from sensorimotor to higher-order cognitive processes. By adopting Yeo's seven-network framework, this study offers a comprehensive approach to examining the effects of aerobic exercise on brain networks, facilitating more consistent and comparable findings across studies.

Another issue to consider is the substantial variability in age among studies examining exercise-induced changes in brain functional networks. While long-term aerobic exercise has been well-documented to enhance cognition and FC in older adults [9], [14], [17], [18], [23], little is known about whether these effects differ across distinct stages of older adulthood. Previous research has mainly focused on group comparisons, either between different age groups or between intervention groups that account for age. However, this approach may mask the unique cognitive and brain health benefits that could be more pronounced at specific ages within the older adult population. For example, preliminary evidence suggests that physical activity is most strongly associated with EF in individuals aged 65 to 73 years [24], although these cross-sectional findings cannot establish causality and may be influenced by confounding factors such as depression. These findings underscore the need for more nuanced investigations to determine whether the protective effects of aerobic exercise interventions on cognition, FC, and their associations are more significant during specific age periods compared to others.

The current study utilized time-varying effect modeling (TVEM) to identify potential critical periods during which aerobic exercise provides the greatest benefits to cognition and brain network FC. The use of TVEM allows for the examination of how the effects of aerobic exercise interventions on cognition and FC vary continuously across different ages. From the perspective of aging and health, identifying specific age ranges where participation in aerobic exercise is most beneficial can help healthcare providers refine recommendations and prioritize exercise interventions for individuals most vulnerable to aging-related effects or at heightened risk of neurodegenerative disorders. Such knowledge does not imply that exercise should be withheld outside these periods but rather highlights potential windows of heightened sensitivity where the benefits of aerobic exercise may be maximized.

Here, we aimed to identify critical age periods through the following primary and exploratory analyses. Our first aim was to determine whether the effects of aerobic exercise training on EF and PS vary with age among older adults engaged in moderate-to-vigorous (ModVig) intensity training compared to light-intensity training (Light). We predicted that older adults in the ModVig group would show greater improvements in EF and PS than those in the Light group, with the magnitude of these differences varying across age. Our second aim was to examine how aerobic exercise training-induced changes in FC across brain networks vary with age among older adults. Given that aging has been associated with reductions in FC and altered functional organization in networks supporting higher-order cognition, we predicted that the ModVig group would exhibit greater FC strength relative to the Light group, potentially reflecting attenuation of these age-related alterations. We further expected that these effects would vary across age, with the strongest increases expected in the DMN and/or SN based on prior findings. Our third aim was to investigate how the magnitude of the associations between aerobic exercise training-induced changes in FC and changes in cognition varies with age. We predicted that the ModVig group would exhibit stronger positive associations between changes in FC and cognition than the Light group, and that these associations would vary across age, potentially reflecting age-related differences in responsiveness to aerobic exercise training.

## 2 Methods

### 2.1 Study design and procedure

**Fig. 1:**
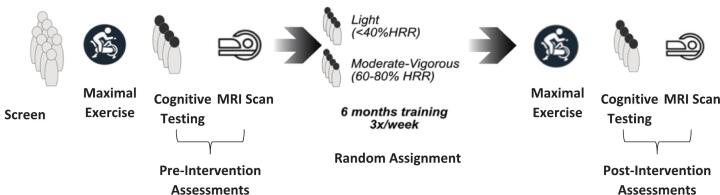
Illustration of the overall study design.

The present study utilized a randomized controlled trial (RCT) to administer exercise intensity doses to non-demented, relatively inactive older adults. Initially, participants completed an orientation session that included informed consent, health history surveys, cognitive screening, and a mock MRI. Those who qualified after these sessions completed a maximal exercise test on a stationary bike to determine their VO_2_ max. This information was used to set up individualized aerobic exercise workloads for subsequent training. Participants then completed cognitive assessments and MRI scans both before and after 6-month aerobic exercise training. They were randomly assigned to one of two exercise training groups: an experimental group engaging in moderate-to-vigorous intensity exercise (ModVig group) or an active control group engaging in primarily light-intensity exercise (Light group). Randomization was performed using a blocked procedure stratified by age group (55–67 yrs., 68–80 yrs.) and biological sex (male/female) to ensure balanced group allocation. The study's procedures were approved by the University of Iowa's Institutional Review Board (IRB), and all participants were compensated in accordance with the IRB's approved procedures. Fig. 1 provides a schematic overview of the study procedures.

**Table 1:** Functional roles of the seven Yeo networks referenced in the study.

Network	Primary Functions	References
Frontoparietal Control Network	Higher-level executive functions, cognitive control	Seeley et al. [63]
Default Mode Network	Reflective thinking, internally generated thoughts (e.g., episodic memory)	Buckner et al. [64]
Dorsal Attention Network	Directs attention to spatially relevant stimuli	Rajan et al. [65]
Limbic Network	Regulates emotions, memory consolidation	Enatsu et al. [66]
Salience Network	Detects salient stimuli, supports cognitive switching	Goulden et al. [67]
Somatomotor Network	Voluntary movements, sensory integration	Biswal et al. [68]
Visual Network	Visual information processing	Yang et al. [69]

### 2.2 Participants

We recruited non-demented older adults, aged 55 to 80 years, at the time of enrollment. Participants were recruited from Iowa City, Iowa, and surrounding communities within a 1-h drive from the University of Iowa campus in Iowa City, IA (approximately a 60-mile radius). Eligible participants met the following criteria: (1) engaged in less than sixty minutes of moderate-intensity physical activity per week in the six months prior to the study, (2) had no history of neurological incidents or disease, (3) had no chronic psychiatric disorders, (4) had a corrected visual acuity of 20/40 or better, (5) were not colorblind, (6) had normal hearing, (7) had no implanted wires or metal devices that could interfere with accurate measurements in MRI, (8) were able to undergo an MRI scan, (9) scored above 20 on the Montreal Cognitive Assessment [25], (10) were native English speakers, and (11) did not take antipsychotic medications or medications for heart disease. After the initial screening, they were further evaluated to determine risk for cardiovascular events and readiness for exercise.

Overall, 107 participants completed the main cognitive assessments, specifically EF and PS tasks, during both the pre- and post-exercise intervention phases. While 122 participants completed MRI data collection during the pre-intervention phase, 24 (*n* = 12 in ModVig; *n* = 12 in Light) were not included for several reasons (e.g., dropped out during intervention, unable to participate due to COVID-19 restrictions) from the post-intervention phase (see Voss et al., 2024). Thus, the final sample consisted of 98 participants who completed MRI data collection during both before and after the intervention and were included for FC analyses. Demographic data for the final sample are summarized in Table 2. Note that due to the discrepancy in maximum ages between the ModVig group (80 years) and the Light group (76 years), direct comparisons for ages 77 to 80 were impossible. Thus, individuals in these age ranges in the ModVig group were truncated to 76 years old.

**Table 2:** Demographic, health, and cognitive characteristics of the sample (*n* = 107).

	ModVig (*n* = 55)	Light (*n* = 52)
Characteristics	*n* (%)	
Female/male, n (%F)	37/18 (67%)	32/20 (62%)
Race		
White/Caucasian	52 (94.5%)	48 (92.4%)
Black	2 (3.7%)	0 (0%)
Asian	1 (1.8%)	2 (3.8%)
Other/More than one	0 (0%)	2 (3.8%)
	Mean (SD)	
Age (years)	63.3 (±6.5)	63.6 (±5.4)
Education (years)	16.4 (±2.1)	16.7 (±2.6)
Health		
Body Mass Index (kg/m^2^)	29.5 (±5.5)	29.3 (±5.5)
Pre-maximal exercise oxygen uptake (ml/kg/min)	19.9 (±5.2)	21.3 (±5.8)
Post-maximal exercise oxygen uptake (ml/kg/min)	22.1 (±5.5)	21.9 (±6.0)
MoCA	27.5 (±1.8)	27.0 (±2.0)

Abbreviations: SD = Standard deviation, MoCA = Montreal cognitive assessment. Note the minimum measured score for MoCA was 23 for each group.

### 2.3 Maximal exercise test

To establish the workload intensity for each participant during the training sessions, participants underwent a maximal exercise test on a stationary bike with 12‑lead electrocardiography (ECG) and cardiopulmonary gas analysis (True One, Parvomedics, Inc.) to determine their VO_2_ max. After attaching the 12‑lead ECG, a supine resting blood pressure measurement was taken, followed by additional blood pressure measurements at each stage of exercise throughout the test. This session was overseen by a trained exercise specialist and a licensed cardiologist from our research team, ensuring both safety and adherence to protocols. For the test to qualify as a true maximal test, participants had to meet two of the following three criteria: (1) a respiratory exchange ratio value of >1.10, (2) ratings of perceived exertion (RPE) of 17 or higher on the 6–20 Borg scale, and/or (3) a maximal heart rate within 10 bpm of the predicted maximal (220-age) or achieving 80% or more of the predicted maximal heart rate. According to these criteria, 87% of participants who completed the test reached their VO_2_ max.

### 2.4 Aerobic exercise training intervention

Participants completed a 6-month RCT of an aerobic exercise training intervention by being assigned to one of two training groups. The ModVig group engaged in a 24-week supervised program led by a certified exercise specialist from our research team with graduate-level training in exercise science and experience in prescribing exercise for older adults. Exercise sessions primarily consisted of aerobic modalities such as treadmill walking and stationary cycling performed at prescribed heart rate reserve (HRR) targets. Training began with sessions including a 5-min warm-up, followed by 20 min of light-intensity aerobic activity at 40% HRR, calculated as [(Peak HR – Resting HR) × percentage] + Resting HR, where resting HR was measured in a seated position after a 5-min rest, then 20 min at 50–60% HRR, and concluded with a 5-min cool-down, occurring three times a week. In each subsequent week, 5 min of exercise at 50–60% HRR were added to each session, and 5 min were subtracted from the light-intensity exercise time, until the total session time reached 40 min by the beginning of week 5. Starting in week 6, the intensity was increased to 60–70% HRR, following a similar build-up protocol to the first 5 weeks. From week 13 onwards, the intensity was further increased to 70–80% HRR, continuing with the same gradual build-up protocol.

In contrast, the Light group engaged in a 24-week supervised program focused on functional flexibility and mobility, also directed by the same exercise specialist. These sessions included low-intensity activities such as slow walking and mobility-based movements designed to maintain HR at or below 40% HRR. Training sessions consisted of a 15-min warm-up with stretching, followed by 20 min of light-intensity aerobic exercise, and ending with 15 min of dynamic stretching to improve range of motion and functional fitness, scheduled three times a week. Additional stretches were introduced each week to diversify and enhance the flexibility of all major muscle groups. The main goal for this group was to keep the intensity at or below 40% HRR during the training sessions.

**Fig. 2:**
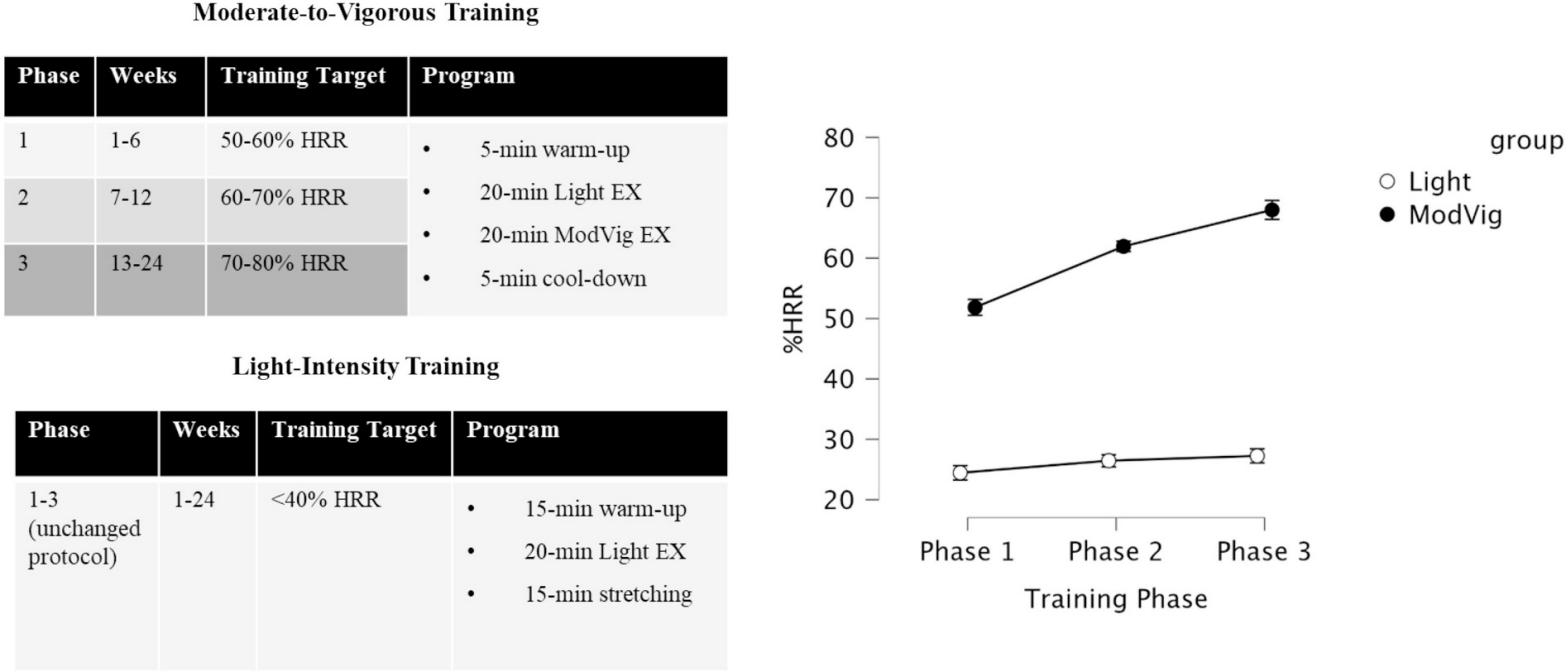
Target %HRR for each of the three phases of the aerobic exercise training intervention and the corresponding mean %HRR achieved during each phase for each group. Abbreviations: HRR = Heart rate reserve, EX = Exercise.

During their exercise sessions, participants from both groups were equipped with a Polar M200 heart rate monitor. The target heart rate for each condition was established using the results from the initial maximal exercise test, which included the peak exercise heart rate used to calculate HRR. Fig. 2 illustrates the three training phases for each group over the 24 weeks and demonstrates how effectively each group met the target %HRR during each training phase. Detailed descriptions of the exercise training protocols and study measures are provided in the published trial protocol [25] and in the associated open materials repository (https://osf.io/ywcq5/).

### 2.5 MRI data acquisition

Participant data was collected on a 3 T GE 750 W Discovery and Signa Premier MRI scanner which used a 32-channel and 48-channel head coil, respectively, at the University of Iowa Magnetic Resonance Research Facility. Participants scanned with the Discovery (D) model (*n* = 52, 42.6%) and Signa Premier (P) model (*n* = 70, 57.4%) did not significantly differ in age, education, sex, or CRF (all *ps* > 0.05), and training groups were nearly balanced in the number of participants scanned at baseline on each model and who had the same scanner pre/post (DD, PP) or a switch (DP) (*Χ*^*2*^(2) = 1.39, *p* = .50) [25]. T1 images were collected for all participants and utilized for registration to each subject's function image. T1 images were collected via a three-dimensional fast spoiled gradient echo sequence (FSPGR) BRAVO T1 scan with the following parameters: TI = 900 ms, TE = 3.168 ms, TR = 8.388 ms, flip angle = 8°, Acquisition Matrix = 256x256x196, FOV = 256x256x196, 1 mm × 1 mm × 1 mm voxels.

Before the 6-month intervention, participants completed two scan sessions within a 48-h acute phase period, each consisting of three 8-min resting-state scans acquired prior to exercise administration. For the present analyses, only the pre-intervention Day 1 session was included. Preliminary exploratory analyses indicated minimal differences in the distribution of FC values for each of the seven networks across Day 1 and Day 2 sessions, suggesting that the repeated scans produced comparable patterns of FC (see Supplementary Fig. 1). In addition, using a single pre-intervention session ensured consistency with the post-intervention protocol, which included one scan session. Within each session, the three resting-state scans were acquired consecutively and concatenated to produce 24 min of resting-state data. Following the 6-month exercise intervention, participants completed one scan session consisting of three 8-min resting-state scans, which were similarly concatenated. Participants were instructed to not exercise for 24 h prior to their pre- and post-intervention scans. During scanning, participants were instructed to focus their eyes on a fixation cross, relax, and remain awake. Wakefulness was monitored by the MRI technician throughout the scan, and no participants were observed to fall asleep. Resting state functional images were acquired with a voxel size of 3.44 × 3.44x4mm with ascending axial slice acquisition and no gap between slices (TE = 30 ms, TR = 2000 ms, flip angle = 80°, FOV = 256x256mm, Matrix size = 64x64x37; 240 volumes for a total scan time of 480 s). Each functional imaging run began with five dummy volumes that were discarded prior to nuisance regression. The T1 and resting state data described are part of a larger scan protocol outside the scope of this study; a detailed listing of scan order and sequence parameters can be found elsewhere [25].

### 2.6 Resting state preprocessing

Preprocessing was completed with FMRIPREP v22.0.2 [26], a Nipype based tool. Each T1-weighted volume was first corrected for signal intensity non-uniformity using N4BiasFieldCorrection and then skull-stripped using antsBrainExtraction.sh (using the Oasis30ANTs template). Brain tissue was segmented into cerebrospinal fluid (CSF), white-matter (WM), and gray-matter (GM) using the brain-extracted T1-weighted images processed with fast (FSL v5.0.9). To reconcile ANTs-derived and FreeSurfer-derived segmentations of the cortical gray-matter, a custom variation of Mindboggle was implemented [27]. Spatial normalization to the ICBM 152 Nonlinear Asymmetrical template version 2009c [28] was performed through nonlinear registration using ANTs [29] with brain-extracted versions of both the T1-weighted volume and the ICBM template. Volume-based spatial normalization to MNI152NLin2009cAsym standard space was performed through nonlinear registration with antsRegistration (ANTs 2.3.3), using brain-extracted versions of both T1w reference and the T1w template.

Next, for each of the resting state BOLD runs, the following preprocessing was performed per subject. First, each BOLD reference was co-registered to the T1w reference using *bbregister* (FreeSurfer), which implements boundary-based registration [30]. Co-registration was configured with six degrees of freedom. Head-motion parameters with respect to the BOLD reference (transformation matrices, and six rotation and translation parameters) were estimated using mcflirt before any spatiotemporal filtering (i.e., slice timing correction; FSL 5.0.9) [31]. Gridded (volumetric) resamplings were performed using antsApplyTransforms (ANTs), configured with Lanczos interpolation to minimize the smoothing effects of other kernels [32]. The BOLD time-series were then resampled into both their original, native space and standard MNI152NLin2009cAsym space by applying the transforms to correct for head-motion. Each resampled, slice time corrected, and preprocessed BOLD run in standard space is now referred to as preprocessed BOLD.

Regressors used in resting-state fMRI preprocessing were measured with FMRIPREP scripts in the following ways. First, an eroded WM and CSF mask was used to extract each signal, respectively, from each preprocessed BOLD image run. An Independent Component Analysis Automated Removal of Motion Artifacts (ICA-AROMA) procedure was implemented with each preprocessed BOLD run to identify structured motion contamination per subject [33]. Each regressor was calculated after removal of non-steady state volumes (5 in total). Temporal filtering (3dBandpass function from AFNI, butterworth filter) was applied to ensure BOLD fluctuations are within a frequency range of 0.01 < f < 0.08 Hz. This range has been identified as optimally sensitive to slow wave rest-state signals needed for functional connectivity analyses. A general linear model was used to remove the influence of each WM, CSF, and aggressive ICA-AROMA noise components from the preprocessed BOLD. Nuisance regression was performed in a single step for each run separately using the recommended xcpEngine aroma design file settings, v1.2.4. [34] [https://github.com/PennLINC/xcpEngine/blob/master/designs/fc-aroma.dsn]. Given ICA-AROMA was applied, and in-scanner head motion is stable within subjects, we did not exclude participants based mean framewise displacement (FD). Supplemental Fig. 2 visualizes the stability of head motion at pre- and post-intervention and the distribution of FD at both time-points; all participants maintained an FD below 0.80 mm.

### 2.7 Resting state functional connectivity of association networks

**Fig. 3:**
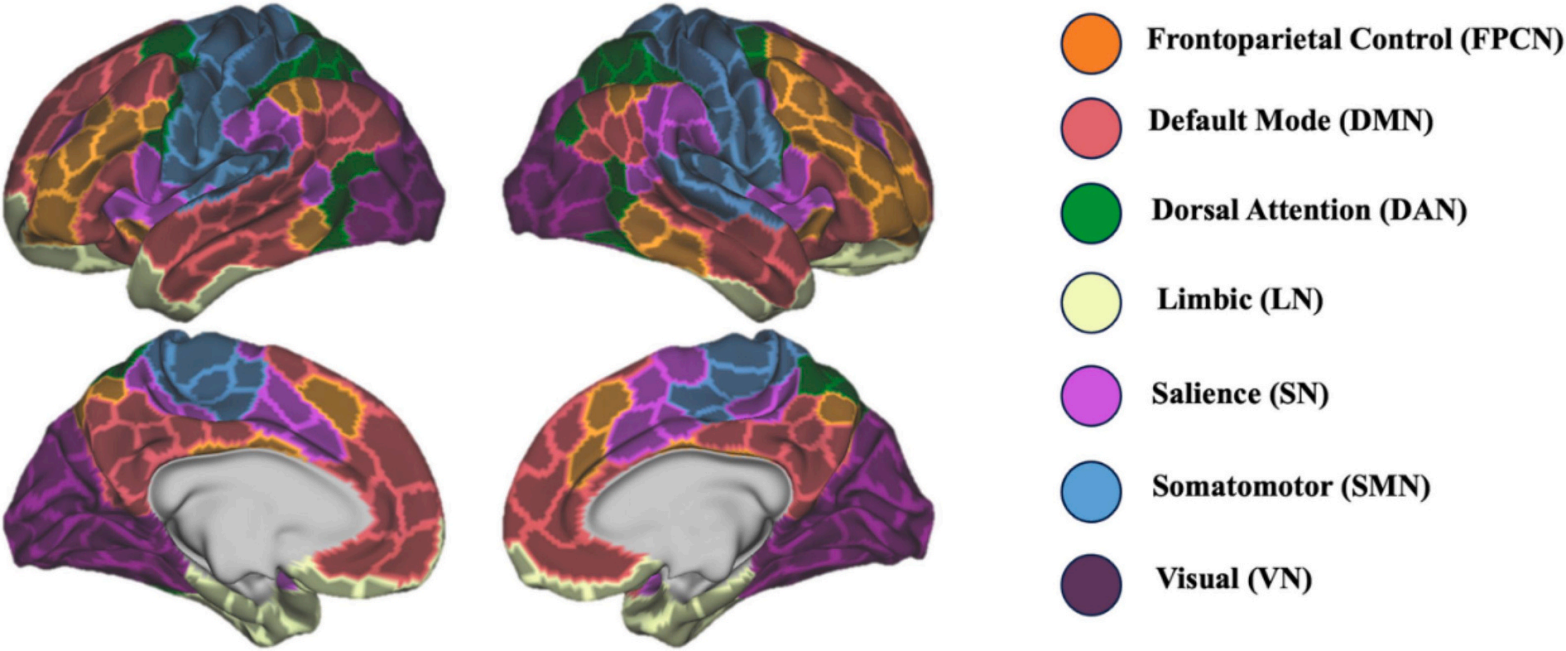
Schaefer 400 Yeo-7 brain network atlas topography for the FPCN (orange), DMN (red), DAN (green), LN (yellow), SN (lilac), SMN (blue), and VN (purple). Abbreviations: FPCN = Frontoparietal control network, DMN = Default mode network, DAN = Dorsal attention network, LN = Limbic network, SN = Salience network, SMN = Somatomotor network, VN = Visual network. (For interpretation of the references to colour in this figure legend, the reader is referred to the web version of this article.)

The residual time-series for each run was transformed into Schaefer 400 parcel Yeo seven network atlas space to extract brain network signals for FC analyses [35]. Our goal was to quantify average rest-state FC for the FPCN, DMN, DAN, LN, SN, SMN, and VN (Fig. 3). We achieved this by first concatenating residual BOLD timeseries from the pre intervention (three runs) session 1, and post intervention (three runs) session, separately to derive pre and post intervention network FC estimates. This resulted in 24 min of rest-state timeseries data to compute each pre and post FC measure. Per participant, FC was defined as the average fisher's z(*r*) of all non-zero pairwise correlations after removal of the center diagonal within a network. Due to signal fallout in limbic regions constituting the temporal poles and orbital frontal cortices, approximately half of the LN parcels were missing in more than 20% of participants in each group. However, as shown in Supplemental Table 1, groups were balanced in the LN parcels affected at each time point and parcels represented in 80% or more of participants included in both the temporal poles and orbital frontal cortices. Additionally, given data were collected with two different MRI coils, that could affect image smoothness and sensitivity to head motion, we verified that preprocessing was effective in minimizing FC differences based on scanner coil (Supplemental Fig. 3).

To evaluate the effect of an aerobic exercise intervention on FC of each brain network, we calculated delta values of FC change for each training group by subtracting the pre-intervention value from the post-intervention value. A higher delta value indicates greater aerobic exercise benefits from the intervention (Table 3).

**Table 3:** Mean (SD) of cognitive task performance and functional connectivity among brain networks pre- and post-intervention.

	ModVig		Light	
Cognitive Tasks (n = 107)	Pre	Post	Pre	Post
Pattern Comparison	16.1 (±2.7)	16.6 (±2.6)	16.3 (±2.7)	16.1 (±2.8)
Letter Comparison	10.7 (±2.1)	10.9 (±2.2)	11.0 (±1.9)	10.7 (±2.0)
DSST	70.6 (±11.9)	74.7 (±17.2)	70.6 (±12.8)	76.9 (±10.6)
Brain Networks FC (*n* = 98)				
Frontoparietal Control	0.25 (±0.05)	0.23 (±0.06)	0.26 (±0.06)	0.25 (±0.06)
Default Mode	0.25 (±0.06)	0.24 (±0.05)	0.26 (±0.06)	0.24 (±0.06)
Dorsal Attention	0.33 (±0.08)	0.33 (±0.07)	0.34 (±0.09)	0.31 (±0.08)
Limbic	0.11 (±0.05)	0.12 (±0.06)	0.12 (±0.05)	0.12 (±0.06)
Salience	0.30 (±0.08)	0.30 (±0.08)	0.31 (±0.08)	0.30 (±0.08)
Somatomotor	0.33 (±0.13)	0.34 (±0.12)	0.34 (±0.14)	0.33 (±0.14)
Visual	0.32 (±0.09)	0.32 (±0.08)	0.34 (±0.08)	0.33 (±0.08)

Note. All cognitive scores were measured based on response accuracy. Abbreviations: SD = Standard deviation, DSST = Digit symbol substitution test, FC = Functional connectivity.

### 2.8 Cognitive assessment

To assess EF, participants completed a paper-and-pencil version of the Digit Symbol Substitution Test (DSST) [36]. For this task, participants were given a sheet of paper with a key at the top that mapped digits 1 through 9 to specific symbols. They were then presented with rows of numbers in a random order and required to write the corresponding symbol in a box below each number. Before the actual test, a practice task featuring seven example trials was administered to ensure participants understood the instructions, and a research assistant checked the accuracy of these practice trials. For the main task, participants had two minutes to fill in as many symbols correctly as possible. The total DSST score, with a maximum possible score of 133, was determined by the number of correct symbols drawn.

To assess PS, participants completed paper-and-pencil versions of the pattern and letter comparison tasks [36]. For the pattern comparison task, participants were asked to compare two patterns and accurately write an “S” (for “same”) or a “D” (for “different”) on the line between them. Participants were to do this for as many of the 30 pattern pairs as possible within 30 s. This process was repeated for a second set of trials. Similarly, for the letter comparison task, participants indicated whether two strings of letters were the same or different by writing an “S” or a “D” on the line between each pair. Participants had 30 s to assess as many of the 15 letter pair strings as possible, a process that was also repeated for a second set of trials. Before the actual tests, an untimed practice page with three example trials were administered to ensure understanding of the tasks, and a research assistant checked the accuracy of these practice trials. For each main task, the total score was determined by averaging the number of correct responses across the two sets.

The main outcome for each cognitive task was the delta score change for each training group from pre to post, a higher delta score indicating greater benefit from the intervention (Table 3). The cognitive assessments described here are from a larger protocol that is outside the scope of this study; a detailed listing of measures described in the RCT protocol paper [25] and in shared materials associated with this paper (https://osf.io/ywcq5/).

### 2.9 Statistical analyses

TVEM analyses were conducted using the TVEM macro package in SAS (version 9.4; accessible at methodology.psu.edu [37]). Unlike traditional regression, TVEM allows regression coefficients to vary at each increment of time (age), enabling the modeling of non-parametric trajectories in regression coefficients on a point-by-point basis along the time axis [38], [39]. This approach uses P-splines to fit data flexibly between each data point, creating smooth slope estimates based on the overall shape of the data. The model estimates include intercept and slope functions that map the association between a predictor and an outcome at each continuous increment of time (or age). In this study, all TVEM models were fit using the P-spline method, which was employed to estimate and visualize parameter functions (see Li et al. [40] and Xie et al. [41] for further details on P-splines in TVEM). Each function was divided into multiple knots (i.e., the splitting points between intervals), and each knot was modeled using a polynomial function (e.g., linear, quadratic, or cubic). Model selection was carried out using a comparative approach by testing various numbers of knots. The process began with five knots, and the shapes of the estimated functions were inspected. If the resulting function appeared relatively simple, the number of knots was gradually reduced, and improvements in model fit were assessed by decreases in the Akaike and Bayesian information criteria (AIC and BIC, respectively). The final model was selected based on the lowest AIC and BIC values [42]. Standard errors were estimated using the Huber-White “sandwich” formula, which adjusts for the correlation among repeated measurements within individuals (see Li et al. [40] for more on sandwich standard errors in TVEM).

Our analyses were conducted in three stages. First, to document how the estimated delta scores for each cognitive task varied across ages 55 to 76, we employed intercept-only models in regression TVEM. These models examine how the mean values of a variable change over age without incorporating other predictors. Separate models were run for the DSST, pattern comparison task, and letter comparison task as measures of EF and PS. Next, to assess the moderating effect of aerobic exercise training on EF and PS performance, intercept-only linear TVEM models were applied separately for older adults in the ModVig group and the Light group. A sample model equation for delta scores in DSST is provided below:

$$∆{\mathrm{DSST}}_{i}=\beta_{0}\left(t\right)+e_{i},$$

where $∆{\mathrm{DSST}}_{i}$ represents the change score in DSST for individual *i*, $\beta_{0}\left(t\right)$ is the intercept function representing the estimated mean change score in DSST as a continuous function of age across 55 to 76 years, and *e*_*i*_ is the error term reflecting individual *i*'s deviation from the curve. This deviation indicates the difference between their observed and expected change score in DSST for an individual of their specific age.

Second, the same model equation was applied, but with the outcome variable changed to document how the estimated delta values in FC varied across ages 55 to 76. Separate models were run for each network. To assess the moderating effect of aerobic exercise training on each network, intercept-only linear TVEM models were applied separately for older adults in the ModVig group and the Light group. A sample model equation for delta values in FC of the FPCN is provided below:

$$∆\mathrm{FPCN}\;{\mathrm{FC}}_{i}=\beta_{0}\left(t\right)+e_{i},$$

where $∆\mathrm{FPCN}\;{\mathrm{FC}}_{i}$ represents the change score in FC of the FPCN for individual *i*, $\beta_{0}\left(t\right)$ is the intercept function representing the estimated mean change score in FC of the FPCN as a continuous function of age across 55 to 76 years, and *e*_*i*_ is the error term reflecting individual *i*'s deviation from the curve. This deviation indicates the difference between their observed and expected change score in FC of the FPCN for an individual of their specific age.

Finally, a linear TVEM model was employed to examine how the association between two variables of interest changes as a function of time (or age), while controlling for covariates. This model was used to evaluate the age-varying associations between changes in cognitive performance and changes in FC induced by exercise training across ages 55 to 76. Separate models were run to analyze how changes in each cognitive task were associated with changes in FC for each brain network across the age span. For example, one model examined how changes in DSST performance were associated with changes in FC of the FPCN. Linear TVEM models for the ModVig and Light groups were analyzed separately to assess the age-varying moderation effects of aerobic exercise training. Sex was included as a time-invariant covariate in all linear TVEM models. Other covariates such as education, medication use, and socioeconomic status were considered but not included in the present models in order to maintain model stability and interpretability within the TVEM framework, given the sample size and exploratory focus of the study. We acknowledge that these factors are important influences on cognitive outcomes and discuss them further as limitations, highlighting their value for future research. We also highlight in Supplemental Fig. 4 that education did not differ as a function of age within each exercise group, suggesting it is unlikely to be a confound in our analyses. A sample model equation for examining the age-varying association between changes in DSST performance and changes in FC of the FPCN is provided below:

$$∆\mathrm{FPCN}\;{\mathrm{FC}}_{i}=\beta_{0}\left(t\right)+\beta_{1}\left(t\right)∆{\mathrm{DSST}}_{i}+\beta_{2}{\mathrm{Sex}}_{i}+e_{i},$$

where $\beta_{1}\left(t\right)$ represents the age-varying association between changes in DSST performance and changes in FC of the FPCN, $\beta_{2}$ captures the time-invariant effect of sex as a covariate, and *e*_*i*_ reflects individual *i*'s residual error term.

All TVEM results are presented in figure format because estimating time-varying coefficients at continuous age intervals produces a large number of coefficients. Detailed numerical values are provided in the supplementary materials. The default setting of 100 time points in the TVEM tables was selected to balance granularity and visual clarity. This setting provides a detailed depiction of how outcome variables change over time while capturing subtle variations. The use of 100 time points ensures that the resulting plots create smooth curves, effectively representing nuances in the data without appearing overly clustered or sparse. Periods where age-specific 95% confidence intervals (CIs) do not include 0 are interpreted as statistically significant at the *p* < .05 level. Additionally, a conservative evaluation of significant differences between groups (ModVig vs. Light) can be made when their respective CIs do not overlap [43]. The randomized controlled trial was powered a priori for the primary trial endpoint (training-related change in learning rate on the task-switch paradigm), as described in the published trial protocol [25]. The present TVEM analyses were not powered to detect age-specific moderation effects and, given the absence of strong prior evidence defining discrete age thresholds, are interpreted as exploratory and hypothesis-generating with respect to potential age windows of responsiveness in exercise training-related changes in BOLD-derived network FC and cognitive performance.

## 3 Results

### 3.1 Model selection

Table 4 summarizes the model fit information for each model used to identify the optimal number of knots for the intercept and slope coefficient functions. The best-fitting model was determined based on the lowest AIC and BIC values, which serve as relative indices of model fit. Model selection was first conducted using the full sample rather than stratifying by exercise group, because splitting the sample at this step would have substantially reduced the sample size in each subgroup, increasing the risk of unstable estimates and overfitting. By using the full sample, we were able to identify an optimal functional form (e.g., balance of smoothness and complexity) that generalized across the dataset while preserving model stability. After establishing this functional form, all subsequent TVEM analyses were conducted separately for the ModVig and Light groups to evaluate group-specific associations.

**Table 4:** Model fit information for intercept and slope coefficient functions using the full sample (ModVig and Light groups combined).

Model	# Knots for Intercept	# Knots for Slope Coefficient	AIC	BIC
∆DSST	5		765.13	790.98
∆Pattern	5		453.80	479.65
∆Letter	5		412.77	438.62
∆FPCNFC	3		226.93	206.84
∆DMNFC	3		222.58	202.49
∆DANFC	2		179.35	161.70
∆LNFC	2		228.20	210.54
∆SNFC	3		197.25	177.16
∆SMNFC	2		192.21	174.56
∆VNFC	2		156.75	139.09
∆DSST∗∆FPCNFC	3	1	162.54	128.34
∆Pattern∗∆FPCNFC	3	1	177.84	143.65
∆Letter∗∆FPCNFC	3	1	180.30	146.10
∆DSST∗∆DMNFC	2	1	161.55	129.64
∆Pattern∗∆DMNFC	2	1	178.91	147.01
∆Letter∗∆DMNFC	2	1	185.48	153.57
∆DSST∗∆DANFC	2	1	117.66	85.76
∆Pattern∗∆DANFC	2	1	133.52	101.61
∆Letter∗∆DANFC	2	1	137.08	105.17
∆DSST∗∆LNFC	2	1	164.53	132.62
∆Pattern∗∆LNFC	2	1	177.22	139.02
∆Letter∗∆LNFC	2	1	176.15	141.96
∆DSST∗∆SNFC	2	1	143.16	111.26
∆Pattern∗∆SNFC	2	1	156.21	124.30
∆Letter∗∆SNFC	2	1	160.96	129.06
∆DSST∗∆SMNFC	2	1	140.50	118.59
∆Pattern∗∆SMNFC	2	1	150.57	128.66
∆Letter∗∆SMNFC	2	1	157.76	128.17
∆DSST∗∆VNFC	2	1	196.48	164.58
∆Pattern∗∆VNFC	2	1	113.41	81.50
∆Letter∗∆VNFC	2	1	116.08	84.17

Note. ∆ represents change scores calculated by subtracting pre-intervention scores from post-intervention scores. Abbreviations: DSST = Digit symbol substitution test, FC = Functional connectivity, FPCN = Frontoparietal control network, DMN = Default mode network, DAN = Dorsal attention network, LN = Limbic network, SN = Salience network, SMN = Somatomotor network, VN = Visual network, AIC = Akaike information criterion, BIC = Bayesian information criterion.

### 3.2 Cognition by age and aerobic exercise training

**Fig. 4:**
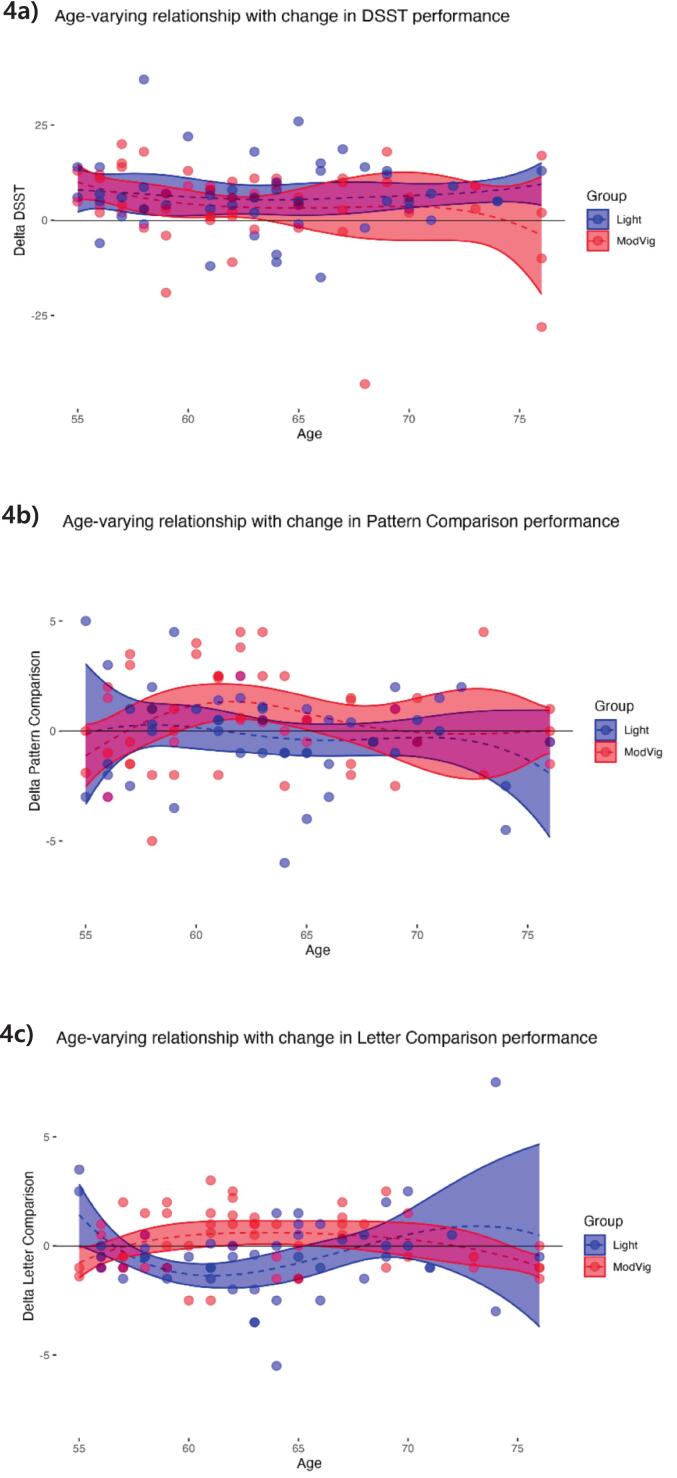
Intercept-only linear TVEM models for changes on the DSST (panel 4a), pattern comparison task (panel 4b), and letter comparison task (panel 4c) across ages 55 to 76 years for the ModVig (red lines) and Light (blue lines) groups. Each observed point is represented by a circle, while the dotted lines indicate the estimated delta scores for each cognitive task. The shaded areas represent the 95% confidence intervals. Abbreviations: DSST = Digit symbol substitution test. (For interpretation of the references to colour in this figure legend, the reader is referred to the web version of this article.)

One goal of the TVEM analysis was to examine how the measures of EF and PS varied across ages 55 to 76, comparing the ModVig group with the Light group. In our intercept-only linear TVEM models, we found no significant differences in delta scores for the DSST (Fig. 4a) or the pattern comparison task (Fig. 4b) between the ModVig and Light groups. However, for the letter comparison task, age-varying patterns in delta scores differed between the groups. These patterns were evident only among individuals aged 58 to 65, with delta scores being higher in the ModVig group than in the Light group based on non-overlapping CIs (Fig. 4c and Supplemental Table 2). The largest difference occurred at age 61, where older adults in the ModVig group had a mean delta score of 0.58 (95% CI = 0.02, 1.14), compared to a mean delta score of −1.35 (95% CI = −1.93, −0.77) in the Light group.

### 3.3 Functional network connectivity by age and aerobic exercise training

**Fig. 5:**
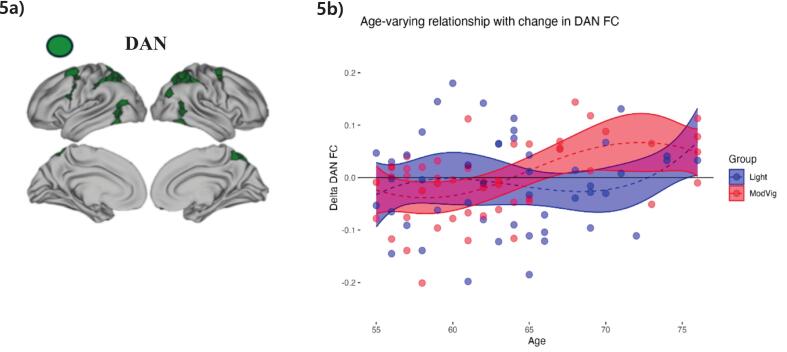
Depicts the cortical topography of Yeo-7 Schaefer atlas DAN (panel 5a). Intercept-only linear TVEM models for changes in FC of the DAN (panel 5b) across ages 55 to 76 years for the ModVig (red lines) and Light (blue lines) groups. Each observed point is represented by a circle, while the dotted lines indicate the estimated delta scores for FC of the DAN. The shaded areas represent the 95% confidence intervals. Abbreviations: DAN = Dorsal attention network, FC = Functional connectivity. (For interpretation of the references to colour in this figure legend, the reader is referred to the web version of this article.)

Another goal of our analysis, using the intercept-only linear TVEM models, was to examine how the average FC of Yeo's seven brain networks varied across ages 55 to 76, comparing the ModVig group with the Light group. Age-varying patterns in delta values of FC in the DAN (Fig. 5b and Supplemental Table 3) differed between the ModVig and Light groups. These differences were apparent only during the ages of 67 to 69 years. Within this age range, changes in FC in the DAN were greater in the ModVig group compared to the Light group. The largest difference in the DAN occurred at age 69, where older adults in the ModVig group had a mean delta score of 0.06 (95% CI = 0.01, 0.10), compared to a mean delta score of −0.03 (95% CI = −0.07, 0.01) in the Light group. No age ranges showed non-overlapping CIs for changes in FC of the FPCN, DMN, LN, SN, SMN, or VN (Supplemental Fig. 5).

### 3.4 Associations between functional connectivity and cognition by age and aerobic exercise training

While adjusting for sex, linear TVEM models were run to examine how associations between changes in FC and changes in cognitive task performance varied across ages 55 to 76, comparing the ModVig group with the Light group.

**Fig. 6:**
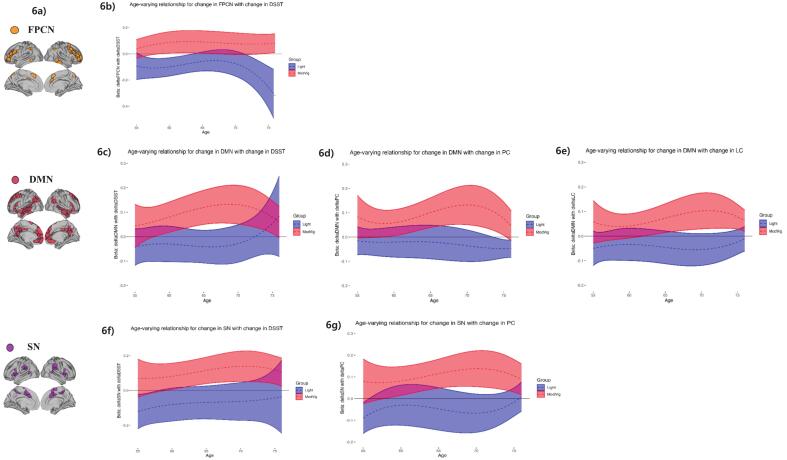
Depicts the cortical topography of Yeo-7 Schaefer atlas FPCN, DMN, and SN (panel 6a). Linear TVEM models for the associations between changes in FC and changes in cognition across ages 55 to 76 years for the ModVig (red lines) and Light (blue lines) groups, while adjusting for sex. Results are presented as regression coefficients (*β,* solid lines). The 95% CIs (shaded area) that do not include 0 (black line) indicate periods of significance. FPCN FC-DSST (panel 6b), DMN FC-DSST (panel 6c), DMN FC-PC (panel 6d), DMN FC-LC (panel 6e), SN FC-DSST (panel 6f), and SN FC-PC (panel 6 g). Abbreviations: FPCN = Frontoparietal control network, DMN = Default mode network, SN = Salience network, FC = Functional connectivity, DSST = Digit symbol substitution test, PC = Pattern comparison, LC = Letter comparison. (For interpretation of the references to colour in this figure legend, the reader is referred to the web version of this article.)

Within the FPCN, a positive association was evident between changes in FC and changes in EF such that a greater increase in FC was associated with greater improvement in DSST performance. This association was observed only in the ModVig group among individuals aged 58 to 64 based on CIs that did not include zero (Fig. 6b and Supplemental Table 4.1.a), with the strongest magnitude observed at age 62 (*β* = 0.09; 95% CI = 0.01, 0.18).

Within the DMN, a positive association was evident between changes in FC and changes in EF such that a greater increase in FC was associated with greater improvement in DSST performance. This association was observed only in the ModVig group among individuals aged 62 to 69 based on CIs that did not include zero (Fig. 6c and Supplemental Table 4.2.a), with the strongest magnitude observed at age 69 (*β* = 0.13; 95% CI = 0.06, 0.21). Similarly, a positive association was evident between changes in FC and changes in PS, where a greater increase in FC was associated with improved performance on both the pattern comparison (Fig. 6d and Supplemental Table 4.2.b) and letter comparison tasks (Fig. 6e and Supplemental Table 4.2.c). These associations were observed only in the ModVig group among individuals aged 66 to 75 based on CIs that did not include zero. For pattern comparison, the strongest magnitude was observed at age 70 (*β* = 0.13; 95% CI = 0.05, 0.21), while for letter comparison, it was observed at age 71 (*β* = 0.10; 95% CI = 0.03, 0.18).

Within the SN, a positive association was evident between changes in FC and changes in EF such that a greater increase in FC was associated with greater improvement in DSST performance. This association was observed only in the ModVig group among individuals aged 62 to 70 based on CIs that did not include zero (Fig. 6f and Supplemental Table 4.3.a), with the strongest magnitude observed at age 70 (*β* = 0.14; 95% CI = 0.05, 0.23). Similarly, a positive association was evident between changes in FC and changes in PS on the pattern comparison task, where a greater increase in FC corresponded to improved performance. This association was observed only in the ModVig group among individuals aged 65 to 74 based on CIs that did not include zero (Fig. 6g and Supplemental Table 4.3.b), with the strongest magnitude observed at age 70 (*β* = 0.14; 95% CI = 0.06, 0.22).

Overall, age-varying changes in the associations between FC and cognition were most evident among individuals in their mid-60s to mid-70s. These findings suggest that this age range may be more responsive to improvements in FC and cognition resulting from moderate-to-vigorous aerobic exercise training; however, these patterns should be interpreted cautiously given the exploratory nature of the analyses. Notably, associations between FC in specific brain networks (FPCN, DMN, and SN) and DSST performance appeared at slightly earlier ages. No age ranges showed non-overlapping CIs between groups for changes in the DAN, SMN, LN, or VN in relation to changes in cognition (Supplemental Fig. 6).

## 4 Discussion

The present study investigated age-related variations in cognition, FC, and their associations in response to six months of aerobic exercise training using a TVEM approach. Older adults in the ModVig group showed improvements in PS, particularly on the letter comparison task, as well as increases in DAN FC at specific ages. Additionally, increases in FC of the FPCN, DMN, and SN were associated with improved performance on measures of PS (pattern comparison and letter comparison) and EF (DSST) among ModVig participants during certain age periods. While more vigorous exercise was associated with improvements in cognition and FC, these effects were not consistent across all age groups, underscoring the importance of considering age-related heterogeneity in response to exercise training.

### 4.1 Age-specific cognitive improvements from aerobic exercise training

Previous studies have demonstrated that aerobic exercise training enhances PS. For instance, Rosano et al. [44] reported that healthy older adults who participated in a one-year moderate-intensity walking program achieved higher PS scores compared to a non-exercise group. Similarly, Smith et al. [45], in a meta-analysis of 24 RCTs involving participants aged 18 to 94, found that aerobic exercise was associated with improvements in PS. However, unlike our findings, their study reported no association between participants' mean age and PS improvements. Our results extend this prior work by identifying ages 58 to 65 as a period during which improvements in PS were most evident, with more vigorous aerobic exercise potentially associated with greater gains in PS (Fig. 4c). Notably, this age range coincided with some of the highest delta scores in the letter comparison task across the sample, suggesting potential beneficial effects of aerobic exercise training on cognitive aging. These effects appeared especially pronounced in tasks requiring rapid visual identification and matching, as demonstrated in the letter comparison task.

Although no significant group differences were observed in EF measures, the DSST was the only task that showed consistently positive delta scores across ages in both groups (Fig. 4a). This pattern suggests potential EF benefits of aerobic exercise training among older adults, regardless of training intensity. Supporting this, Chen et al. [23] reported in a meta-analysis that both middle-aged (55–65 years) and older adults (66–75 years) experienced EF improvements from long-term exercise training compared to sedentary counterparts.

### 4.2 Age-specific variations in functional connectivity in select brain networks from aerobic exercise training

Our TVEM analysis highlights the importance of age- and network-specific variations in FC following aerobic exercise training among older adults. Contrary to our prediction, the ModVig group exhibited increases in FC within the DAN, particularly between ages 67 and 69 (Fig. 5b). While these findings align with prior work showing that aerobic exercise modifies functional brain networks in older adults [9], [46], [47], the use of TVEM revealed unique, age-specific patterns in the DAN that would not have been detected with conventional analytic methods. This pattern may suggest that aerobic exercise intensity is associated with variation in within-network FC across age and should be viewed as exploratory evidence of age-varying patterns, rather than discrete periods of heightened sensitivity, during which aerobic training may help preserve cognitive and motor function before steeper age-related declines occur.

Observed changes in FC during the late 60s may reflect underlying neurophysiological processes. As the brain ages, especially beyond the late 60s, it undergoes natural declines in both functional and structural integrity, including reductions in WM fiber systems [48], [49]. The age range highlighted in our study overlaps with this period of heightened vulnerability, when moderate-to-vigorous exercise may be beneficial in offsetting declines. Several mechanisms have been also proposed to explain how aerobic exercise may be associated with cognitive aging processes. Candidate pathways include (1) upregulation of neurotrophic factors such as BDNF [50], [51], [52], [53] and (2) enhanced mitochondrial function and density [54], which may support neuronal signaling and the incorporation of new neurons into brain networks, potentially contributing to FC integrity [55]. Improved cerebrovascular health is another plausible mechanism. Aerobic exercise enhances blood flow and vascular function, facilitating oxygen and nutrient delivery and supporting synaptic plasticity. These vascular and cellular adaptations may contribute to maintaining more resilient networks despite structural aging [16], [56]. Although it is difficult to pinpoint precise mechanisms for DAN-related changes, neurotransmitter systems may play a role. Aerobic exercise increases the release of norepinephrine (NE) and dopamine (DA), which may enhance attention and responsiveness by modulating DAN activity [57], [58]. While prior work in younger adults has shown that acute exercise-induced NE and DA changes regulate DAN FC [59], long-term exercise may also induce adaptive changes in receptor function. Although our study did not directly test this hypothesis, prior evidence highlights it as a plausible pathway through which aerobic training may be associated with functional network plasticity and cognitive resilience in aging populations. Together, these cellular, vascular, and neurochemical adaptations may contribute to strengthening large-scale network integrity and increasing the likelihood that training-related changes in FC are associated with cognitive performance. In this context, higher intensity aerobic training may be associated with stronger FC-cognition coupling, particularly at ages when these networks retain sufficient plastic potential despite age-related vulnerability to structural damage and atrophy.

Our results may help contextualize why some networks, particularly the DMN, appeared less responsive to aerobic training in the present trial. Specifically, we did not observe age ranges with non-overlapping CIs for DMN FC, despite previous reports of exercise-related increases in DMN FC [9], [12]. A possible explanation relates to our relatively active comparison condition. Participants in the Light group engaged in regular low-intensity aerobic physical activity, which may have been sufficient to maintain DMN function and thereby reduce contrasts with the ModVig group. In prior work demonstrating training-related increases in DMN FC, aerobic training has typically been compared with non-aerobic flexibility/toning controls that impose minimal cardiovascular load, producing larger between-group effects [9]. Moreover, both our sample and Voss et al.'s [9] trial included similar average ages (63 vs. 65 years) and nearly identical age ranges (55–80 years). Given this overlap and the fact that our trial compared two aerobic intensities rather than aerobic training versus a non-aerobic control condition, between-group differences in DMN outcomes may have been inherently attenuated. Accordingly, differences in intervention design and control conditions may be more likely explanations for the absence of DMN effects than age per se. Future studies could extend this work by testing a broader range of aerobic doses (e.g., greater separation in training intensity or volume across aerobic condition) to more precisely characterize the conditions under which DMN FC is most responsive to aerobic exercise.

### 4.3 Aerobic exercise training effects on the associations between functional connectivity and cognition by age

Prior work has established associations between aerobic exercise-related increases in FC and cognitive improvements in healthy older adults [9], [47], [53]. Our study extends this evidence by showing that increases in FC within the FPCN, DMN and SN were associated with improvements in EF and PS, specifically among ModVig participants in their mid-60s to mid-70s. Importantly, this is the first aerobic exercise trial in older adults to directly compare two levels of aerobic intensity when examining FC-cognition associations, allowing us to identify age-specific patterns that would be obscured in group-average analyses.

The role of CRF in these associations is noteworthy. Cross-sectional studies have shown that higher CRF is linked to better cognition, possibly through enhanced FC [9]. CRF is thought to be a key pathway through which regular exercise induces structural, functional, and vascular adaptations that preserve brain and cognitive health during aging [16]. In our sample, exploratory analyses revealed modest VO_2_ max improvements in the ModVig group, particularly among adults aged 63 to 68 (see Supplemental Fig. 7). Notably, this age range overlapped with the period in which FC-cognition associations were most evident, particularly for the DMN and SN. Although these findings may suggest a potential role of CRF in exercise-related changes in brain function and cognition, future work is needed to directly test the mechanisms linking fitness adaptations with neural and cognitive changes following exercise training.

Interestingly, the FC-cognition associations appeared relatively consistent compared with the more distinct age-specific patterns observed for cognition and FC examined separately. For example, while significant associations between FC and DSST performance were found in the FPCN, DMN and SN exclusively in the ModVig group (Fig. 6b, c, f), no age ranges showed non-overlapping CIs between groups for DSST performance alone (Fig. 4a). This pattern suggests that aerobic exercise intensity may be associated with variation in the coupling between FC changes and cognitive performance, rather than consistent differences in FC or cognition in isolation.

Collectively, these findings suggest that higher-intensity aerobic exercise training may be associated with age-related patterns linking FC and cognition, even when differences in cognitive change alone are not apparent. Rather than indicating broad, age-spanning benefits, the relatively flat associations outside midlife-to-early-old-age highlight variability in these relationships across age, with the mid-60s to mid-70s reflecting a range in which these associations were most apparent.

### 4.4 Limitations and future directions

This study used a novel TVEM approach to compare the effects of light- versus moderate-to-vigorous intensity aerobic exercise on age-specific changes in brain network FC and cognition. While these analyses provide valuable insights, several limitations should be considered. First, only 15% of participants were over 70 years old, limiting the stability of estimates in this age group and resulting in wider CIs. Greater sample density at older ages would yield more reliable estimates along the smoothed curve. Nonetheless, TVEM remains a robust approach that leverages all available data to model age-varying effects [60]. Second, our sample lacked racial and ethnic diversity, which constrains generalizability. We acknowledge that racial-ethnic disparities influence both cognitive aging [61] and physical activity participation [62]. Future studies should recruit more diverse samples to clarify population-level benefits of exercise training. Finally, our TVEM models included only sex as a covariate to preserve model stability and interpretability. Although factors such as education, medication use, and socioeconomic status are important for cognitive outcomes, they were not incorporated here. Including these covariates in future work would provide a more comprehensive understanding of exercise-cognition links.

Importantly, the overall lack of consistent group-level differences regardless of age suggests that group-average comparisons may obscure meaningful variability when both conditions involve aerobic activity. Thus, null findings at the group level do not necessarily imply an absence of exercise effects but rather highlight nuanced, age-varying responses in exercise-induced changes that conventional regression methods may fail to capture. To address this limitation, we employed TVEM in the main study, enabling us to examine how changes in cognition and FC vary continuously with age in response to exercise training. Relatedly, the age ranges highlighted in this study should be viewed as exploratory and used to inform future a priori, confirmatory analyses in independent samples of older adults. In this context, it is also worth noting that the training-group sample sizes in this trial are comparable to or larger than those in prior studies reporting exercise-training effects on BOLD-derived FC [9].

## 5 Conclusion

This study underscores the importance of considering age-related variability when evaluating the effects of aerobic exercise on FC and cognition in older adults. Using a TVEM approach, we found that six months of moderate-to-vigorous training was associated with increases in FC in the FPCN, DMN, and SN, which were also associated with improvements in EF and PS. These patterns were most evident in adults in their mid-60s to mid-70s, suggesting that more vigorous aerobic activity may be associated with greater benefits during this period. Future studies with more diverse samples are needed to replicate these findings and to clarify the mechanisms linking aerobic exercise, brain FC, and cognition across different stages of aging.

## CRediT authorship contribution statement

**Myungjin Jung:** Writing – review & editing, Writing – original draft, Validation, Software, Project administration, Methodology, Formal analysis, Data curation, Conceptualization. **Marco Pipoly:** Writing – review & editing, Visualization, Formal analysis. **Bryan Madero:** Writing – review & editing, Investigation, Formal analysis. **Chris Oehler:** Resources, Investigation. **Kelsey Baller:** Writing – review & editing, Investigation. **Vincent A. Magnotta:** Writing – review & editing. **Jeffrey D. Long:** Writing – review & editing. **Gary L. Pierce:** Writing – review & editing. **Michelle W. Voss:** Writing – review & editing, Visualization, Supervision, Project administration, Funding acquisition.

## Funding

This work was supported by 10.13039/100000049National Institute on Aging (R01 AG055500). MR imaging was performed on equipment supported by High-end instrumentation grant S10OD025025.

## Declaration of competing interest

The authors declare that they have no known competing financial interests or personal relationships that could have appeared to influence the work reported in this paper.

## Footnotes

1 The functional roles of the seven networks examined in this study are summarized in Table 1, which outlines their primary cognitive and neural functions.

## References

[1] Park D.C., Reuter-Lorenz P. (2009). The adaptive brain: aging and neurocognitive scaffolding. Annu Rev Psychol.

[2] Salthouse T.A. (2017). Contributions of the individual differences approach to cognitive aging. J Gerontol B Psychol Sci Soc Sci.

[3] Hausman H.K., O’Shea A., Kraft J.N., Boutzoukas E.M., Evangelista N.D., Van Etten E.J. (2020). The role of resting-state network functional connectivity in cognitive aging. Front Aging Neurosci.

[4] Salthouse T.A. (2000). Aging and measures of processing speed. Biol Psychol.

[5] Jack C.R., Wiste H.J., Weigand S.D. (2014). Age-specific population frequencies of cerebral β-amyloidosis and neurodegeneration among people with normal cognitive function aged 50-89 years: a cross-sectional study. Lancet Neurol.

[6] Zhang Z., Chan M.Y., Han L. (2023). Dissociable effects of Alzheimer’s disease-related cognitive dysfunction and aging on functional brain network segregation. J Neurosci.

[7] Buckley R.F., Schultz A.P., Hedden T. (2017). Functional network integrity presages cognitive decline in preclinical Alzheimer disease. Neurology.

[8] Erickson K.I., Hillman C., Stillman C.M., Ballard R.M., Bloodgood B., Conroy D.E. (2019). Physical activity, cognition, and brain outcomes: a review of the 2018 physical activity guidelines. Med Sci Sports Exerc.

[9] Voss M.W., Prakash R.S., Erickson K.I., Basak C., Chaddock L., Kim J.S. (2010). Plasticity of brain networks in a randomized intervention trial of exercise training in older adults. Front Aging Neurosci.

[10] Sporns O. (2013). Structure and function of complex brain networks. Dialogues Clin Neurosci.

[11] Flodin P., Jonasson L.S., Riklund K., Nyberg L., Boraxbekk C.J. (2017). Does aerobic exercise influence intrinsic brain activity? An aerobic exercise intervention among healthy old adults. Front Aging Neurosci.

[12] McGregor K.M., Crosson B., Krishnamurthy L.C., Krishnamurthy V., Hortman K., Gopinath K. (2018). Effects of a 12-week aerobic spin intervention on resting state networks in previously sedentary older adults. Front Psychol.

[13] Tozzi L., Carballedo A., Lavelle G., Doolin K., Doyle M., Amico F. (2016). Longitudinal functional connectivity changes correlate with mood improvement after regular exercise in a dose-dependent fashion. Eur J Neurosci.

[14] Voss M.W., Weng T.B., Burzynska A.Z., Wong C.N., Cooke G.E., Clark R. (2016). Fitness, but not physical activity, is related to functional integrity of brain networks associated with aging. Neuroimage.

[15] Won J., Zaborszky L., Purcell J.J., Ranadive S.M., Gentili R.J., Smith J.C. (2023). Basal forebrain functional connectivity as a mediator of associations between cardiorespiratory fitness and cognition in healthy older women. Brain Imaging Behav.

[16] Voss M.W., Jain S. (2022). Getting fit to counteract cognitive aging: evidence and future directions. Physiology (Bethesda).

[17] Voss M.W., Erickson K.I., Prakash R.S., Chaddock L., Malkowski E., Alves H. (2010). Functional connectivity: a source of variance in the association between cardiorespiratory fitness and cognition?. Neuropsychologia.

[18] Won J., Nielson K.A., Smith J.C. (2023). Large-scale network connectivity and cognitive function changes after exercise training in older adults with intact cognition and mild cognitive impairment. J Alzheimers Dis Rep.

[19] Chirles T.J., Reiter K., Weiss L.R., Alfini A.J., Nielson K.A., Smith J.C. (2017). Exercise training and functional connectivity changes in mild cognitive impairment and healthy elders. J Alzheimer’s Dis.

[20] McFadden K.L., Cornier M.A., Melanson E.L., Bechtell J.L., Tregellas J.R. (2013). Effects of exercise on resting-state default mode and salience network activity in overweight/obese adults. Neuroreport.

[21] Dorsman K.A., Weiner-Light S., Staffaroni A.M., Brown J.A., Wolf A., Cobigo Y. (2020). Get moving! Increases in physical activity are associated with increasing functional connectivity trajectories in typically aging adults. Front Aging Neurosci.

[22] Yeo B.T., Krienen F.M., Sepulcre J., Sabuncu M.R., Lashkari D., Hollinshead M. (2011). The organization of the human cerebral cortex estimated by intrinsic functional connectivity. J Neurophysiol.

[23] Chen F.T., Etnier J.L., Chan K.H., Chiu P.K., Hung T.M., Chang Y.K. (2020). Effects of exercise training interventions on executive function in older adults: a systematic review and meta-analysis. Sports Med.

[24] Jung M., Lee S., Kang M., Allen H.K. (2023). Age-varying association between depression symptoms and executive function among older adults: moderation by physical activity. J Psychiatr Res.

[25] Voss M.W., Oehler C., Daniels W., Sodoma M., Madero B., Kent J. (2024). Exercise effects on brain health and learning from minutes to months: the brain EXTEND trial. Contemp Clin Trials.

[26] Esteban O., Markiewicz C.J., Blair R.W., Moodie C.A., Isik A.I., Erramuzpe A. (2019). fMRIPrep: a robust preprocessing pipeline for functional MRI. Nat Methods.

[27] Klein A., Ghosh S.S., Bao F.S., Giard J., Häme Y., Stavsky E. (2017). Mindboggling morphometry of human brains. PLoS Comput Biol.

[28] Fonov V., Coupe P., Eskildsen S.F., Collins L.D. (2011). Alzheimer’s Association International Conference.

[29] Avants B.B., Epstein C.L., Grossman M., Gee J.C. (2008). Symmetric diffeomorphic image registration with cross-correlation: evaluating automated labeling of elderly and neurodegenerative brain. Med Image Anal.

[30] Greve D.N., Fischl B. (2009). Accurate and robust brain image alignment using boundary-based registration. Neuroimage.

[31] Jenkinson M., Bannister P., Brady M., Smith S. (2002). Improved optimization for the robust and accurate linear registration and motion correction of brain images. Neuroimage.

[32] Lanczos C. (1964). A precision approximation of the gamma function. J SIAM Numer ANAL.

[33] Pruim R.H., Mennes M., van Rooij D., Llera A., Buitelaar J.K., Beckmann C.F. (2015). ICA-AROMA: a robust ICA-based strategy for removing motion artifacts from fMRI data. Neuroimage.

[34] Ciric R., Rosen A.F., Erus G., Cieslak M., Adebimpe A., Cook P.A. (2018). Mitigating head motion artifact in functional connectivity MRI. Nat Protoc.

[35] Schaefer A., Kong R., Gordon E.M., Laumann T.O., Zuo X.N., Holmes A.J. (2018). Local-global parcellation of the human cerebral cortex from intrinsic functional connectivity MRI. Cereb Cortex.

[36] Earles J.L., Salthouse T.A. (1995). Interrelations of age, health, and speed. J Gerontol B Psychol Sci Soc Sci.

[37] Li R., Dziak J.J., Tan X., Huang L., Wagner A.T., Yang J. (2015). http://methodology.psu.edu/.

[38] Lanza S.T., Vasilenko S.A., Liu X., Li R., Piper M.E. (2013). Advancing the understanding of craving during smoking cessation attempts: a demonstration of the time-varying effect model. Nicotine Tob Res.

[39] Tan X., Shiyko M.P., Li R., Li Y., Dierker L. (2012). A time-varying effect model for intensive longitudinal data. Psychol Methods.

[40] Li R., Dziak J.J., Huang L., Wagner A.T., Yang J. (2017). http://methodology.psu.edu/.

[41] Xie H., Drake R.E., Kim S.J., McHugo G.J. (2017). Analyzing long-duration and high-frequency data using the time-varying effect model. Adm Policy Ment Health.

[42] Shiyko M.P., Lanza S.T., Tan X., Li R., Shiffman S. (2012). Using the time-varying effect model (TVEM) to examine dynamic associations between negative affect and self confidence on smoking urges: differences between successful quitters and relapsers. Prev Sci.

[43] Vasilenko S.A. (2017). Age-varying associations between nonmarital sexual behavior and depressive symptoms across adolescence and young adulthood. Dev Psychol.

[44] Rosano C., Venkatraman V.K., Guralnik J., Newman A.B., Glynn N.W., Launer L. (2010). Psychomotor speed and functional brain MRI 2 years after completing a physical activity treatment. J Gerontol A Biol Sci Med Sci.

[45] Smith P.J., Blumenthal J.A., Hoffman B.M., Cooper H., Strauman T.A., Welsh-Bohmer K. (2010). Aerobic exercise and neurocognitive performance: a meta-analytic review of randomized controlled trials. Psychosom Med.

[46] Won J., Callow D.D., Pena G.S., Jordan L.S., Arnold-Nedimala N.A., Nielson K.A. (2021). Hippocampal functional connectivity and memory performance after exercise intervention in older adults with mild cognitive impairment. J Alzheimer’s Dis.

[47] Won J., Faroqi-Shah Y., Callow D.D., Williams A., Awoyemi A., Nielson K.A. (2021). Association between greater cerebellar network connectivity and improved phonemic fluency performance after exercise training in older adults. Cerebellum.

[48] Mendez Colmenares A., Prytherch B., Thomas M.L., Burzynska A.Z. (2023). Within-person changes in the aging white matter microstructure and their modifiers: a meta-analysis and systematic review of longitudinal diffusion tensor imaging studies. Imaging Neurosci.

[49] de Kort F.A.S., Vinke E.J., van der Lelij E.J. (2025). Cerebral white matter hyperintensity volumes: normative age- and sex-specific values from 15 population-based cohorts comprising 14,876 individuals. Neurobiol Aging.

[50] Moore D., Jung M., Hillman C.H., Kang M., Loprinzi P.D. (2022). Interrelationships between exercise, functional connectivity, and cognition among healthy adults: a systematic review. Psychophysiology.

[51] Rasmussen P., Brassard P., Adser H., Pedersen M.V., Leick L., Hart E. (2009). Evidence for a release of brain-derived neurotrophic factor from the brain during exercise. Exp Physiol.

[52] Seifert T., Brassard P., Wissenberg M., Rasmussen P., Nordby P., Stallknecht B. (2010). Endurance training enhances BDNF release from the human brain. Am J Physiol Regul Integr Comp Physiol.

[53] Voss M.W., Erickson K.I., Prakash R.S., Chaddock L., Kim J.S., Alves H. (2013). Neurobiological markers of exercise-related brain plasticity in older adults. Brain Behav Immun.

[54] Won J., Callow D.D., Pena G.S., Gogniat M.A., Kommula Y., Arnold-Nedimala N.A. (2021). Evidence for exercise-related plasticity in functional and structural neural network connectivity. Neurosci Biobehav Rev.

[55] Steib K., Schäffner I., Jagasia R., Ebert B., Lie D.C. (2014). Mitochondria modify exercise-induced development of stem cell-derived neurons in the adult brain. J Neurosci.

[56] Davenport M.H., Hogan D.B., Eskes G.A., Longman R.S., Poulin M.J. (2012). Cerebrovascular reserve: the link between fitness and cognitive function?. Exerc Sport Sci Rev.

[57] McMorris T. (2016). Developing the catecholamines hypothesis for the acute exercise-cognition interaction in humans: lessons from animal studies. Physiol Behav.

[58] McMorris T. (2021). The acute exercise-cognition interaction: from the catecholamines hypothesis to an interoception model. Int J Psychophysiol.

[59] Mather M., Huang R., Clewett D., Nielsen S.E., Velasco R., Tu K. (2020). Isometric exercise facilitates attention to salient events in women via the noradrenergic system. Neuroimage.

[60] Lanza S.T., Linden-Carmichael A.N. (2021). time-varying effect modeling for the behavioral, social, and health sciences.

[61] Peterson R.L., Fain M.J., Butler E., Ehiri J.E., Carvajal S.C. (2020). The role of social and behavioral risk factors in explaining racial disparities in age-related cognitive impairment: a structured narrative review. Neuropsychol Dev Cogn B Aging Neuropsychol Cogn.

[62] Jung M., Kim H., Ryu S., Kang M. (2021). Secular trends in physical activity among immigrants in the United States, 2009–2018. J Phys Act Health.

[63] Seeley W.W., Menon V., Schatzberg A.F., Keller J., Glover G.H., Kenna H. (2007). Dissociable intrinsic connectivity networks for salience processing and executive control. J Neurosci.

[64] Buckner R.L., Andrews-Hanna J.R., Schacter D.L. (2008). The brain’s default network: anatomy, function, and relevance to disease. Ann N Y Acad Sci.

[65] Rajan A., Meyyappan S., Liu Y., Samuel I.B.H., Nandi B., Mangun G.R. (2021). The microstructure of attentional control in the dorsal attention network. J Cogn Neurosci.

[66] Enatsu R., Gonzalez-Martinez J., Bulacio J., Kubota Y., Mosher J., Burgess R.C. (2015). Connections of the limbic network: a corticocortical evoked potentials study. Cortex.

[67] Goulden N., Khusnulina A., Davis N.J., Bracewell R.M., Bokde A.L., McNulty J.P. (2014). The salience network is responsible for switching between the default mode network and the central executive network: replication from DCM. Neuroimage.

[68] Biswal B., Zerrin Yetkin F., Haughton V.M., Hyde J.S. (1995). Functional connectivity in the motor cortex of resting human brain using echo-planar MRI. Magn Reson Med.

[69] Yang Y.L., Deng H.X., Xing G.Y., Xia X.L., Li H.F. (2015). Brain functional network connectivity based on a visual task: visual information processing-related brain regions are significantly activated in the task state. Neural Regen Res.

