# Supplementary material for: Effects of aerobic exercise intervention on the association between brain functional connectivity and cognition among older adults
Source: Aging Brain. 2026 Jul 28;10:100162. doi: 10.1016/j.nbas.2026.100162 (PMC13450027; doi:10.1016/j.nbas.2026.100162)
Supplement: Supplementary file 1 [file mmc1.docx]

**Supplementary Information**

For exploratory purposes, a multiple regression analysis was conducted to examine whether there were significant differences in changes in each cognitive task and functional connectivity (FC) of each brain network based on age (as a continuous variable), sex (male vs. female), and training group (ModVig vs. Light). Change scores were calculated by subtracting pre-intervention scores from post-intervention scores for each outcome variable. Cognitive performance was assessed using the DSST (a measure of EF) and the pattern and letter comparison tasks (measures of PS). Functional network connectivity was measured using resting-state fMRI for the FPCN, DMN, DAN, LN, SN, SMN, and VN.

Results indicated that neither age, sex, nor training group significantly predicted changes in each cognitive task, all *ps* > .05. Similarly, these predictors did not significantly predict changes in FC of each brain network, all *ps* > .05. However, the training group showed a statistically significant difference in changes in FC of the LN, with the ModVig group exhibiting greater changes compared to the Light group, *β* = .035, *SE* = .012, *t* = 2.873, *p* = .005. These findings suggest that conventional regression methods may fail to capture nuanced, age-specific variations in exercise-induced changes. To address this limitation, we employed TVEM in the main manuscript, enabling us to examine how changes in cognition and FC vary continuously with age in response to exercise training.

**Supplemental Figure Captions**

*Figure 1.* Distribution of Fisher z-transformed functional connectivity (FC) values across pre-intervention scan sessions (Day 1 and Day 2) for each of the seven brain networks. Density plots illustrate the distribution of network-level FC values. Abbreviations: FPCN = Frontoparietal control network, DMN = Default mode network, DAN = Dorsal attention network, LN = Limbic network, SN = Salience network, SMN = Somatomotor network, VN = Visual network.

*Figure 2.* Stability of in-scanner head motion at baseline and post-intervention. Scatterplot of mean framewise displacement at baseline (x-axis) versus post-intervention (y-axis) with marginal distributions.

*Figure 3.* Scanner coil differences in network FC at pre- and post-intervention. Mean network FC for the Discovery and Premier scan models across Yeo 7 networks at pre- (top) and post-intervention (bottom).

*Figure 4.* Education levels by age bin and training group. Mean years of education across 5-year age bins for the Light and Moderate-to-vigorous (ModVig) intensity training groups.

*Figure 5.*	Intercept-only linear TVEM models reveal a non-significant difference in changes in FC of the FPCN (panel 1a), DMN (panel 1b), LN (panel 1c), SN (panel 1d), SMN (panel 1e), and VN (panel 1f) across ages 55 to 76 for the ModVig (red lines) and Light (blue lines) groups. Each observed point is represented by a circle, while the dotted lines indicate the estimated delta scores for FC across each of the brain networks. The shaded areas represent the 95% confidence intervals. Abbreviations: FC = Functional connectivity, FPCN = Frontoparietal control network, DMN = Default mode network, LN = Limbic network, SN = Salience network, SMN = Somatomotor network, VN = Visual network.

*Figure 6.*	While adjusting for sex, across ages 55 to 76, linear TVEM models reveal a non-significant association between changes in FC and changes in cognition within the FPCN (panel 2.1.b and 2.1.c), DAN (panel 2.2.a-2.2.c), LN (panel 2.3.a-2.3.c), SN (panel 2.4.c), SMN (panel 2.5.a-2.5.c), and VN (panel 2.6.a-2.6.c) for the ModVig (red lines) and Light (blue lines) groups. Results are presented as regression coefficients (*β*). The 95% CIs (shaded area) that do not include 0 (black line) indicate periods of significance. FPCN FC-PC (panel 2.1.b), FPCN FC-LC (panel 2.1.c), DAN FC-DSST (panel 2.2.a), DAN FC-PC (panel 2.2.b), DAN FC-LC (panel 2.2.c), LN FC-DSST (panel 2.3.a), LN FC-PC (panel 2.3.b), LN FC-LC (panel 2.3.c), SN-LC (panel 2.4.c), SMN-DSST (panel 2.5.a), SMN-PC (panel 2.5.b), SMN-LC (panel 2.5.c), VN-DSST (panel 2.6.a), VN-PC (panel 2.6.b), and VN-LC (panel 2.6.c). *Note*. Significant effects observed in FPCN FC-DSST, DMN FC-DSST, DMN FC-PC, DMN FC-LC, SN-DSST, and SN-PC are presented in Figure 6 of the main manuscript and are thus not included in the supplementary material. Panels labeled with 'a' correspond to DSST, 'b' to PC, and 'c' to LC. Abbreviations: FC = Functional connectivity, FPCN = Frontoparietal control network, DAN = Dorsal attention network, LN = Limbic network, SN = Salience network, SMN = Somatomotor network, VN = Visual network. DSST = Digital symbol substitution test, PC = Pattern comparison, LC = Letter comparison.

*Figure 7.*	An intercept-only linear TVEM model for changes in VO_2_ max across ages 55 to 76 for the ModVig (red lines) and Light (blue lines) groups. Each observed point is represented by a circle, while the dotted lines indicate the estimated delta scores for VO_2_ max. The shaded areas represent the 95% confidence intervals.

**Supplemental Figure 1.**

**Supplemental Figure 2.**

**Supplemental Figure 3.**

**Supplemental Figure 4.**

**Supplemental Figure 5.**

**1d)**

**1a)**

**1e)**

**1b)**

**1f)**

**1c)**

**Supplemental Figure 6.**

**2.1.c)**

**2.1.b)**

**2.2.c)**

**2.2.a)**

**2.2.b)**

**2.3.a)**

**2.3.b)**

**2.3.c)**

**2.4.c)**

**2.5.a)**

**2.5.b)**

**2.5.c)**

**2.6.a)**

**2.6.b)**

**2.6.c)**

**Supplemental Figure 7.**
