# Supplementary material for: Effects of aerobic exercise intervention on the association between brain functional connectivity and cognition among older adults
Source: Aging Brain. 2026 Jul 28;10:100162. doi: 10.1016/j.nbas.2026.100162 (PMC13450027; doi:10.1016/j.nbas.2026.100162)
Supplement: Supplementary file 2 [file mmc2.docx]

**Supplemental Table 1**

To check representativeness of parcels in the atlas in our data, parcels in the Yeo7 Schaefer400 atlas were flagged as “high missing” if more than 20% of subjects in an intervention group were missing data from the parcel in at least one of three runs. Only parcels from the Limbic network were identified at either baseline and post-intervention. Tables below summarize how many parcels were identified and their corresponding labels.

**Baseline:** For the light group, 50% of the parcels in the Limbic network were flagged as high missing, which is comparable to 46% of parcels flagged as high missing in the ModVig group.

| group | n_group | ROI | n_missing_gt0 | prop_missing_gt0 | high_missing_20 | network |
| --- | --- | --- | --- | --- | --- | --- |
| Light | 52 | LH_Limbic_OFC_1 | 35 | 0.6730769 | 1 | Limbic |
| Light | 52 | LH_Limbic_OFC_4 | 20 | 0.3846154 | 1 | Limbic |
| Light | 52 | LH_Limbic_TempPole_1 | 22 | 0.4230769 | 1 | Limbic |
| Light | 52 | LH_Limbic_TempPole_2 | 46 | 0.8846154 | 1 | Limbic |
| Light | 52 | LH_Limbic_TempPole_3 | 22 | 0.4230769 | 1 | Limbic |
| Light | 52 | LH_Limbic_TempPole_4 | 45 | 0.8653846 | 1 | Limbic |
| Light | 52 | RH_Limbic_OFC_1 | 30 | 0.5769231 | 1 | Limbic |
| Light | 52 | RH_Limbic_OFC_3 | 41 | 0.7884615 | 1 | Limbic |
| Light | 52 | RH_Limbic_OFC_5 | 35 | 0.6730769 | 1 | Limbic |
| Light | 52 | RH_Limbic_TempPole_1 | 34 | 0.6538462 | 1 | Limbic |
| Light | 52 | RH_Limbic_TempPole_2 | 20 | 0.3846154 | 1 | Limbic |
| Light | 52 | RH_Limbic_TempPole_3 | 33 | 0.6346154 | 1 | Limbic |
| Light | 52 | RH_Limbic_TempPole_4 | 11 | 0.2115385 | 1 | Limbic |
| ModVig | 55 | LH_Limbic_OFC_1 | 38 | 0.6909091 | 1 | Limbic |
| ModVig | 55 | LH_Limbic_OFC_4 | 30 | 0.5454545 | 1 | Limbic |
| ModVig | 55 | LH_Limbic_TempPole_1 | 28 | 0.5090909 | 1 | Limbic |
| ModVig | 55 | LH_Limbic_TempPole_2 | 46 | 0.8363636 | 1 | Limbic |
| ModVig | 55 | LH_Limbic_TempPole_3 | 25 | 0.4545455 | 1 | Limbic |
| ModVig | 55 | LH_Limbic_TempPole_4 | 42 | 0.7636364 | 1 | Limbic |
| ModVig | 55 | RH_Limbic_OFC_1 | 35 | 0.6363636 | 1 | Limbic |
| ModVig | 55 | RH_Limbic_OFC_3 | 42 | 0.7636364 | 1 | Limbic |
| ModVig | 55 | RH_Limbic_OFC_5 | 43 | 0.7818182 | 1 | Limbic |
| ModVig | 55 | RH_Limbic_TempPole_1 | 37 | 0.6727273 | 1 | Limbic |
| ModVig | 55 | RH_Limbic_TempPole_2 | 22 | 0.4000000 | 1 | Limbic |
| ModVig | 55 | RH_Limbic_TempPole_3 | 37 | 0.6727273 | 1 | Limbic |

**Baseline parcels** **in Limbic system that were *not* flagged as high missing** are listed below, and show representation across the anatomical areas affected by signal drop-out such as orbitofrontal cortex and temporal pole. Thus the remaining parcels are representative of the major sub-regions within the Limbic system.

| group | n_group | ROI | n_missing_gt0 | prop_missing_gt0 | high_missing_20 | network |
| --- | --- | --- | --- | --- | --- | --- |
| Light | 52 | LH_Limbic_OFC_2 | 0 | 0.00000000 | 0 | Limbic |
| Light | 52 | LH_Limbic_OFC_3 | 7 | 0.13461538 | 0 | Limbic |
| Light | 52 | LH_Limbic_OFC_5 | 0 | 0.00000000 | 0 | Limbic |
| Light | 52 | LH_Limbic_TempPole_5 | 4 | 0.07692308 | 0 | Limbic |
| Light | 52 | LH_Limbic_TempPole_6 | 8 | 0.15384615 | 0 | Limbic |
| Light | 52 | LH_Limbic_TempPole_7 | 2 | 0.03846154 | 0 | Limbic |
| Light | 52 | LH_Limbic_TempPole_8 | 3 | 0.05769231 | 0 | Limbic |
| Light | 52 | RH_Limbic_OFC_2 | 1 | 0.01923077 | 0 | Limbic |
| Light | 52 | RH_Limbic_OFC_4 | 0 | 0.00000000 | 0 | Limbic |
| Light | 52 | RH_Limbic_OFC_6 | 3 | 0.05769231 | 0 | Limbic |
| Light | 52 | RH_Limbic_TempPole_5 | 8 | 0.15384615 | 0 | Limbic |
| Light | 52 | RH_Limbic_TempPole_6 | 7 | 0.13461538 | 0 | Limbic |
| Light | 52 | RH_Limbic_TempPole_7 | 10 | 0.19230769 | 0 | Limbic |
| ModVig | 55 | LH_Limbic_OFC_2 | 0 | 0.00000000 | 0 | Limbic |
| ModVig | 55 | LH_Limbic_OFC_3 | 10 | 0.18181818 | 0 | Limbic |
| ModVig | 55 | LH_Limbic_OFC_5 | 0 | 0.00000000 | 0 | Limbic |
| ModVig | 55 | LH_Limbic_TempPole_5 | 7 | 0.12727273 | 0 | Limbic |
| ModVig | 55 | LH_Limbic_TempPole_6 | 3 | 0.05454545 | 0 | Limbic |
| ModVig | 55 | LH_Limbic_TempPole_7 | 3 | 0.05454545 | 0 | Limbic |
| ModVig | 55 | LH_Limbic_TempPole_8 | 4 | 0.07272727 | 0 | Limbic |
| ModVig | 55 | RH_Limbic_OFC_2 | 3 | 0.05454545 | 0 | Limbic |
| ModVig | 55 | RH_Limbic_OFC_4 | 0 | 0.00000000 | 0 | Limbic |
| ModVig | 55 | RH_Limbic_OFC_6 | 4 | 0.07272727 | 0 | Limbic |
| ModVig | 55 | RH_Limbic_TempPole_4 | 9 | 0.16363636 | 0 | Limbic |
| ModVig | 55 | RH_Limbic_TempPole_5 | 10 | 0.18181818 | 0 | Limbic |
| ModVig | 55 | RH_Limbic_TempPole_6 | 10 | 0.18181818 | 0 | Limbic |
| ModVig | 55 | RH_Limbic_TempPole_7 | 5 | 0.09090909 | 0 | Limbic |

**Post-intervention parcels flagged as high missing**: For the light group, 46% of the parcels in the Limbic network were flagged as high missing, which is comparable to 46% of parcels flagged as high missing in the ModVig group.

| group | n_group | ROI | n_missing_gt0 | prop_missing_gt0 | high_missing_20 | network |
| --- | --- | --- | --- | --- | --- | --- |
| Light | 52 | LH_Limbic_OFC_1 | 26 | 0.5000000 | 1 | Limbic |
| Light | 52 | LH_Limbic_OFC_4 | 19 | 0.3653846 | 1 | Limbic |
| Light | 52 | LH_Limbic_TempPole_1 | 24 | 0.4615385 | 1 | Limbic |
| Light | 52 | LH_Limbic_TempPole_2 | 38 | 0.7307692 | 1 | Limbic |
| Light | 52 | LH_Limbic_TempPole_3 | 22 | 0.4230769 | 1 | Limbic |
| Light | 52 | LH_Limbic_TempPole_4 | 34 | 0.6538462 | 1 | Limbic |
| Light | 52 | RH_Limbic_OFC_1 | 24 | 0.4615385 | 1 | Limbic |
| Light | 52 | RH_Limbic_OFC_3 | 33 | 0.6346154 | 1 | Limbic |
| Light | 52 | RH_Limbic_OFC_5 | 31 | 0.5961538 | 1 | Limbic |
| Light | 52 | RH_Limbic_TempPole_1 | 28 | 0.5384615 | 1 | Limbic |
| Light | 52 | RH_Limbic_TempPole_2 | 19 | 0.3653846 | 1 | Limbic |
| Light | 52 | RH_Limbic_TempPole_3 | 24 | 0.4615385 | 1 | Limbic |
| ModVig | 55 | LH_Limbic_OFC_1 | 33 | 0.6000000 | 1 | Limbic |
| ModVig | 55 | LH_Limbic_OFC_4 | 23 | 0.4181818 | 1 | Limbic |
| ModVig | 55 | LH_Limbic_TempPole_1 | 27 | 0.4909091 | 1 | Limbic |
| ModVig | 55 | LH_Limbic_TempPole_2 | 41 | 0.7454545 | 1 | Limbic |
| ModVig | 55 | LH_Limbic_TempPole_3 | 23 | 0.4181818 | 1 | Limbic |
| ModVig | 55 | LH_Limbic_TempPole_4 | 39 | 0.7090909 | 1 | Limbic |
| ModVig | 55 | RH_Limbic_OFC_1 | 32 | 0.5818182 | 1 | Limbic |
| ModVig | 55 | RH_Limbic_OFC_3 | 37 | 0.6727273 | 1 | Limbic |
| ModVig | 55 | RH_Limbic_OFC_5 | 39 | 0.7090909 | 1 | Limbic |
| ModVig | 55 | RH_Limbic_TempPole_1 | 33 | 0.6000000 | 1 | Limbic |
| ModVig | 55 | RH_Limbic_TempPole_2 | 19 | 0.3454545 | 1 | Limbic |
| ModVig | 55 | RH_Limbic_TempPole_3 | 35 | 0.6363636 | 1 | Limbic |

**Post-intervention parcels in Limbic system that were *not* flagged as high missing are listed below**, and show representation across the anatomical areas affected by signal drop-out such as orbitofrontal cortex and temporal pole. Thus the remaining parcels are representative of the major sub-regions within the Limbic system.

| group | n_group | ROI | n_missing_gt0 | prop_missing_gt0 | high_missing_20 | network |
| --- | --- | --- | --- | --- | --- | --- |
| Light | 52 | LH_Limbic_OFC_2 | 0 | 0.00000000 | 0 | Limbic |
| Light | 52 | LH_Limbic_OFC_3 | 3 | 0.05769231 | 0 | Limbic |
| Light | 52 | LH_Limbic_OFC_5 | 1 | 0.01923077 | 0 | Limbic |
| Light | 52 | LH_Limbic_TempPole_5 | 7 | 0.13461538 | 0 | Limbic |
| Light | 52 | LH_Limbic_TempPole_6 | 5 | 0.09615385 | 0 | Limbic |
| Light | 52 | LH_Limbic_TempPole_7 | 3 | 0.05769231 | 0 | Limbic |
| Light | 52 | LH_Limbic_TempPole_8 | 1 | 0.01923077 | 0 | Limbic |
| Light | 52 | RH_Limbic_OFC_2 | 0 | 0.00000000 | 0 | Limbic |
| Light | 52 | RH_Limbic_OFC_4 | 0 | 0.00000000 | 0 | Limbic |
| Light | 52 | RH_Limbic_OFC_6 | 3 | 0.05769231 | 0 | Limbic |
| Light | 52 | RH_Limbic_TempPole_4 | 10 | 0.19230769 | 0 | Limbic |
| Light | 52 | RH_Limbic_TempPole_5 | 9 | 0.17307692 | 0 | Limbic |
| Light | 52 | RH_Limbic_TempPole_6 | 6 | 0.11538462 | 0 | Limbic |
| Light | 52 | RH_Limbic_TempPole_7 | 4 | 0.07692308 | 0 | Limbic |
| ModVig | 55 | LH_Limbic_OFC_2 | 0 | 0.00000000 | 0 | Limbic |
| ModVig | 55 | LH_Limbic_OFC_3 | 6 | 0.10909091 | 0 | Limbic |
| ModVig | 55 | LH_Limbic_OFC_5 | 0 | 0.00000000 | 0 | Limbic |
| ModVig | 55 | LH_Limbic_TempPole_5 | 8 | 0.14545455 | 0 | Limbic |
| ModVig | 55 | LH_Limbic_TempPole_6 | 7 | 0.12727273 | 0 | Limbic |
| ModVig | 55 | LH_Limbic_TempPole_7 | 6 | 0.10909091 | 0 | Limbic |
| ModVig | 55 | LH_Limbic_TempPole_8 | 5 | 0.09090909 | 0 | Limbic |
| ModVig | 55 | RH_Limbic_OFC_2 | 1 | 0.01818182 | 0 | Limbic |
| ModVig | 55 | RH_Limbic_OFC_4 | 0 | 0.00000000 | 0 | Limbic |
| ModVig | 55 | RH_Limbic_OFC_6 | 2 | 0.03636364 | 0 | Limbic |
| ModVig | 55 | RH_Limbic_TempPole_4 | 7 | 0.12727273 | 0 | Limbic |
| ModVig | 55 | RH_Limbic_TempPole_5 | 11 | 0.20000000 | 0 | Limbic |
| ModVig | 55 | RH_Limbic_TempPole_6 | 10 | 0.18181818 | 0 | Limbic |
| ModVig | 55 | RH_Limbic_TempPole_7 | 6 | 0.10909091 | 0 | Limbic |

**Supplemental Table 2.** Age-varying relationship with change in Letter Comparison performance.

| Age | ModVig_L  (Lower 95% CI) | $\Delta\mathrm{Letter}$  in ModVig | ModVig_U  (Upper 95% CI) | SE of  $\Delta\mathrm{letter}$ | Light_L  (Lower 95% CI) | $\Delta\mathrm{Letter}$  in Light | Light_U  (Upper 95% CI) |
| --- | --- | --- | --- | --- | --- | --- | --- |
| 55.00 | -1.47 | -0.78 | -0.08 | 0.71 | 0.02 | 1.42 | 2.82 |
| 55.21 | -1.31 | -0.68 | -0.05 | 0.64 | -0.07 | 1.19 | 2.45 |
| 55.42 | -1.16 | -0.59 | -0.02 | 0.58 | -0.15 | 0.98 | 2.11 |
| 55.64 | -1.03 | -0.50 | 0.03 | 0.52 | -0.24 | 0.78 | 1.79 |
| 55.85 | -0.91 | -0.42 | 0.08 | 0.46 | -0.32 | 0.58 | 1.49 |
| 56.06 | -0.82 | -0.34 | 0.14 | 0.42 | -0.41 | 0.41 | 1.22 |
| 56.27 | -0.73 | -0.26 | 0.21 | 0.37 | -0.50 | 0.24 | 0.97 |
| 56.48 | -0.66 | -0.19 | 0.28 | 0.34 | -0.59 | 0.08 | 0.74 |
| 56.70 | -0.60 | -0.13 | 0.35 | 0.31 | -0.68 | -0.07 | 0.53 |
| 56.91 | -0.55 | -0.06 | 0.42 | 0.28 | -0.77 | -0.21 | 0.35 |
| 57.12 | -0.51 | -0.01 | 0.50 | 0.26 | -0.86 | -0.34 | 0.18 |
| 57.33 | -0.47 | 0.05 | 0.56 | 0.25 | -0.95 | -0.46 | 0.03 |
| 57.55 | -0.43 | 0.10 | 0.63 | 0.24 | -1.04 | -0.57 | -0.10 |
| 57.76 | -0.39 | 0.15 | 0.69 | 0.23 | -1.13 | -0.67 | -0.21 |
| 57.97 | -0.36 | 0.19 | 0.75 | 0.23 | -1.21 | -0.76 | -0.31 |
| **58.18** | **-0.33** | **0.23** | **0.80** | **0.23** | **-1.30** | **-0.85** | **-0.40** |
| **58.39** | **-0.30** | **0.27** | **0.85** | **0.23** | **-1.38** | **-0.93** | **-0.48** |
| **58.61** | **-0.27** | **0.31** | **0.89** | **0.23** | **-1.45** | **-1.00** | **-0.54** |
| **58.82** | **-0.25** | **0.34** | **0.93** | **0.23** | **-1.52** | **-1.06** | **-0.60** |
| **59.03** | **-0.22** | **0.37** | **0.96** | **0.24** | **-1.58** | **-1.12** | **-0.65** |
| **59.24** | **-0.20** | **0.40** | **0.99** | **0.24** | **-1.64** | **-1.17** | **-0.70** |
| **59.45** | **-0.17** | **0.42** | **1.02** | **0.24** | **-1.69** | **-1.21** | **-0.74** |
| **59.67** | **-0.15** | **0.45** | **1.04** | **0.25** | **-1.74** | **-1.25** | **-0.77** |
| **59.88** | **-0.13** | **0.47** | **1.06** | **0.25** | **-1.78** | **-1.28** | **-0.79** |
| **60.09** | **-0.10** | **0.49** | **1.08** | **0.26** | **-1.81** | **-1.31** | **-0.81** |
| **60.30** | **-0.08** | **0.51** | **1.09** | **0.26** | **-1.84** | **-1.33** | **-0.82** |
| **60.52** | **-0.06** | **0.52** | **1.11** | **0.27** | **-1.87** | **-1.35** | **-0.83** |
| **60.73** | **-0.05** | **0.54** | **1.12** | **0.27** | **-1.89** | **-1.36** | **-0.83** |
| **60.94** | **-0.03** | **0.55** | **1.12** | **0.28** | **-1.90** | **-1.36** | **-0.82** |
| **61.15** | **-0.01** | **0.56** | **1.13** | **0.28** | **-1.92** | **-1.36** | **-0.81** |
| **61.36** | **0.00** | **0.57** | **1.13** | **0.29** | **-1.93** | **-1.36** | **-0.79** |
| **61.58** | **0.02** | **0.58** | **1.14** | **0.30** | **-1.93** | **-1.35** | **-0.77** |
| **61.79** | **0.03** | **0.58** | **1.14** | **0.30** | **-1.93** | **-1.34** | **-0.75** |
| **62.00** | **0.04** | **0.59** | **1.14** | **0.31** | **-1.93** | **-1.32** | **-0.72** |
| **62.21** | **0.05** | **0.60** | **1.14** | **0.32** | **-1.93** | **-1.30** | **-0.68** |
| **62.42** | **0.06** | **0.60** | **1.14** | **0.33** | **-1.92** | **-1.28** | **-0.64** |
| **62.64** | **0.07** | **0.61** | **1.14** | **0.33** | **-1.91** | **-1.25** | **-0.60** |
| **62.85** | **0.07** | **0.61** | **1.14** | **0.34** | **-1.89** | **-1.22** | **-0.56** |
| **63.06** | **0.08** | **0.61** | **1.14** | **0.35** | **-1.87** | **-1.19** | **-0.51** |
| **63.27** | **0.08** | **0.61** | **1.14** | **0.35** | **-1.85** | **-1.16** | **-0.46** |
| **63.48** | **0.08** | **0.61** | **1.14** | **0.36** | **-1.82** | **-1.12** | **-0.42** |
| **63.70** | **0.08** | **0.61** | **1.14** | **0.36** | **-1.79** | **-1.08** | **-0.36** |
| **63.91** | **0.08** | **0.61** | **1.14** | **0.37** | **-1.76** | **-1.03** | **-0.31** |
| **64.12** | **0.08** | **0.61** | **1.14** | **0.37** | **-1.72** | **-0.99** | **-0.26** |
| **64.33** | **0.07** | **0.61** | **1.14** | **0.37** | **-1.68** | **-0.94** | **-0.21** |
| **64.55** | **0.07** | **0.60** | **1.14** | **0.38** | **-1.63** | **-0.90** | **-0.16** |
| **64.76** | **0.06** | **0.60** | **1.14** | **0.38** | **-1.58** | **-0.85** | **-0.11** |
| **64.97** | **0.05** | **0.59** | **1.14** | **0.37** | **-1.53** | **-0.79** | **-0.06** |
| **65.18** | **0.04** | **0.59** | **1.14** | **0.37** | **-1.47** | **-0.74** | **-0.01** |
| 65.39 | 0.03 | 0.58 | 1.14 | 0.37 | -1.41 | -0.69 | 0.04 |
| 65.61 | 0.01 | 0.58 | 1.14 | 0.37 | -1.35 | -0.63 | 0.09 |
| 65.82 | 0.00 | 0.57 | 1.14 | 0.36 | -1.29 | -0.58 | 0.14 |
| 66.03 | -0.02 | 0.56 | 1.14 | 0.36 | -1.22 | -0.52 | 0.19 |
| 66.24 | -0.03 | 0.55 | 1.14 | 0.36 | -1.16 | -0.46 | 0.23 |
| 66.45 | -0.05 | 0.54 | 1.14 | 0.35 | -1.09 | -0.40 | 0.28 |
| 66.67 | -0.07 | 0.53 | 1.14 | 0.35 | -1.02 | -0.35 | 0.33 |
| 66.88 | -0.09 | 0.52 | 1.14 | 0.34 | -0.96 | -0.29 | 0.38 |
| 67.09 | -0.11 | 0.51 | 1.13 | 0.34 | -0.89 | -0.23 | 0.43 |
| 67.30 | -0.13 | 0.50 | 1.13 | 0.33 | -0.83 | -0.17 | 0.49 |
| 67.52 | -0.15 | 0.49 | 1.13 | 0.33 | -0.76 | -0.11 | 0.54 |
| 67.73 | -0.17 | 0.48 | 1.13 | 0.33 | -0.71 | -0.05 | 0.60 |
| 67.94 | -0.19 | 0.47 | 1.12 | 0.34 | -0.66 | 0.01 | 0.67 |
| 68.15 | -0.22 | 0.45 | 1.12 | 0.34 | -0.61 | 0.06 | 0.74 |
| 68.36 | -0.24 | 0.44 | 1.12 | 0.36 | -0.58 | 0.12 | 0.82 |
| 68.58 | -0.27 | 0.42 | 1.11 | 0.37 | -0.55 | 0.18 | 0.90 |
| 68.79 | -0.29 | 0.41 | 1.11 | 0.39 | -0.53 | 0.23 | 0.99 |
| 69.00 | -0.32 | 0.39 | 1.10 | 0.41 | -0.52 | 0.29 | 1.09 |
| 69.21 | -0.35 | 0.37 | 1.09 | 0.44 | -0.52 | 0.34 | 1.19 |
| 69.42 | -0.38 | 0.35 | 1.08 | 0.47 | -0.52 | 0.39 | 1.30 |
| 69.64 | -0.41 | 0.33 | 1.07 | 0.50 | -0.54 | 0.44 | 1.42 |
| 69.85 | -0.44 | 0.31 | 1.06 | 0.54 | -0.56 | 0.49 | 1.54 |
| 70.06 | -0.47 | 0.29 | 1.05 | 0.57 | -0.59 | 0.54 | 1.66 |
| 70.27 | -0.50 | 0.27 | 1.04 | 0.62 | -0.63 | 0.58 | 1.79 |
| 70.48 | -0.53 | 0.24 | 1.02 | 0.66 | -0.67 | 0.62 | 1.91 |
| 70.70 | -0.57 | 0.22 | 1.00 | 0.71 | -0.72 | 0.66 | 2.04 |
| 70.91 | -0.60 | 0.19 | 0.98 | 0.75 | -0.77 | 0.70 | 2.17 |
| 71.12 | -0.63 | 0.16 | 0.95 | 0.80 | -0.83 | 0.73 | 2.30 |
| 71.33 | -0.66 | 0.13 | 0.93 | 0.85 | -0.90 | 0.77 | 2.43 |
| 71.55 | -0.69 | 0.10 | 0.90 | 0.90 | -0.97 | 0.80 | 2.56 |
| 71.76 | -0.72 | 0.07 | 0.86 | 0.95 | -1.04 | 0.82 | 2.69 |
| 71.97 | -0.75 | 0.04 | 0.83 | 1.00 | -1.12 | 0.85 | 2.82 |
| 72.18 | -0.78 | 0.00 | 0.78 | 1.06 | -1.21 | 0.87 | 2.94 |
| 72.39 | -0.81 | -0.04 | 0.74 | 1.11 | -1.30 | 0.88 | 3.06 |
| 72.61 | -0.84 | -0.08 | 0.69 | 1.17 | -1.39 | 0.89 | 3.18 |
| 72.82 | -0.87 | -0.12 | 0.64 | 1.22 | -1.49 | 0.90 | 3.30 |
| 73.03 | -0.89 | -0.16 | 0.58 | 1.28 | -1.60 | 0.91 | 3.41 |
| 73.24 | -0.92 | -0.20 | 0.52 | 1.33 | -1.70 | 0.91 | 3.52 |
| 73.45 | -0.94 | -0.25 | 0.45 | 1.39 | -1.82 | 0.91 | 3.63 |
| 73.67 | -0.97 | -0.29 | 0.38 | 1.45 | -1.94 | 0.90 | 3.73 |
| 73.88 | -0.99 | -0.34 | 0.31 | 1.50 | -2.06 | 0.89 | 3.84 |
| 74.09 | -1.01 | -0.39 | 0.23 | 1.56 | -2.20 | 0.87 | 3.93 |
| 74.30 | -1.04 | -0.45 | 0.14 | 1.62 | -2.33 | 0.85 | 4.03 |
| 74.52 | -1.06 | -0.50 | 0.06 | 1.68 | -2.48 | 0.82 | 4.12 |
| 74.73 | -1.09 | -0.56 | -0.02 | 1.74 | -2.63 | 0.79 | 4.20 |
| 74.94 | -1.13 | -0.62 | -0.11 | 1.80 | -2.79 | 0.75 | 4.29 |
| 75.15 | -1.17 | -0.68 | -0.19 | 1.87 | -2.95 | 0.71 | 4.37 |
| 75.36 | -1.22 | -0.74 | -0.26 | 1.93 | -3.13 | 0.66 | 4.45 |
| 75.58 | -1.29 | -0.81 | -0.33 | 2.00 | -3.31 | 0.61 | 4.52 |
| 75.79 | -1.37 | -0.88 | -0.38 | 2.07 | -3.50 | 0.55 | 4.60 |
| 76.00 | -1.48 | -0.95 | -0.41 | 2.14 | -3.70 | 0.48 | 4.67 |

*Note.* $\Delta$Letter values represent the estimated mean differences in letter comparison task scores between pre- and post-intervention for each group. The age range highlighted in bold indicates where significant differences between the two groups were found. Abbreviations: SE = Standard error, CI = Confidence interval.

**Supplemental Table 3.** Age-varying relationships with change in DAN FC.

| Age | ModVig_L  (Lower 95% CI) | $\Delta DAN$  in ModVig | ModVig_U  (Upper 95% CI) | SE of  $\Delta DAN$ | Light_L  (Lower 95% CI) | $\Delta DAN$  in Light | Light_U  (Upper 95% CI) |
| --- | --- | --- | --- | --- | --- | --- | --- |
| 55.00 | -0.069 | -0.024 | 0.022 | 0.031 | -0.094 | -0.033 | 0.029 |
| 55.21 | -0.067 | -0.025 | 0.017 | 0.029 | -0.086 | -0.030 | 0.027 |
| 55.42 | -0.066 | -0.027 | 0.012 | 0.027 | -0.079 | -0.027 | 0.026 |
| 55.64 | -0.065 | -0.029 | 0.007 | 0.025 | -0.073 | -0.024 | 0.025 |
| 55.85 | -0.064 | -0.030 | 0.004 | 0.023 | -0.067 | -0.021 | 0.024 |
| 56.06 | -0.064 | -0.032 | 0.001 | 0.022 | -0.063 | -0.019 | 0.025 |
| 56.27 | -0.064 | -0.033 | -0.001 | 0.021 | -0.059 | -0.017 | 0.025 |
| 56.48 | -0.065 | -0.034 | -0.003 | 0.021 | -0.056 | -0.015 | 0.026 |
| 56.70 | -0.065 | -0.035 | -0.005 | 0.021 | -0.053 | -0.013 | 0.027 |
| 56.91 | -0.066 | -0.036 | -0.006 | 0.021 | -0.052 | -0.011 | 0.029 |
| 57.12 | -0.067 | -0.037 | -0.006 | 0.021 | -0.050 | -0.010 | 0.031 |
| 57.33 | -0.067 | -0.037 | -0.007 | 0.021 | -0.049 | -0.008 | 0.032 |
| 57.55 | -0.068 | -0.038 | -0.008 | 0.021 | -0.049 | -0.007 | 0.034 |
| 57.76 | -0.068 | -0.038 | -0.008 | 0.022 | -0.049 | -0.006 | 0.036 |
| 57.97 | -0.069 | -0.038 | -0.008 | 0.022 | -0.049 | -0.005 | 0.038 |
| 58.18 | -0.069 | -0.038 | -0.008 | 0.023 | -0.049 | -0.004 | 0.040 |
| 58.39 | -0.069 | -0.038 | -0.008 | 0.023 | -0.049 | -0.004 | 0.042 |
| 58.61 | -0.068 | -0.038 | -0.008 | 0.024 | -0.049 | -0.003 | 0.043 |
| 58.82 | -0.068 | -0.038 | -0.008 | 0.024 | -0.049 | -0.002 | 0.045 |
| 59.03 | -0.067 | -0.038 | -0.008 | 0.024 | -0.050 | -0.002 | 0.046 |
| 59.24 | -0.067 | -0.037 | -0.008 | 0.025 | -0.050 | -0.002 | 0.047 |
| 59.45 | -0.066 | -0.037 | -0.007 | 0.025 | -0.051 | -0.002 | 0.047 |
| 59.67 | -0.065 | -0.036 | -0.007 | 0.025 | -0.051 | -0.002 | 0.048 |
| 59.88 | -0.064 | -0.035 | -0.006 | 0.025 | -0.051 | -0.002 | 0.048 |
| 60.09 | -0.062 | -0.034 | -0.006 | 0.025 | -0.051 | -0.002 | 0.048 |
| 60.30 | -0.061 | -0.033 | -0.005 | 0.025 | -0.052 | -0.002 | 0.048 |
| 60.52 | -0.060 | -0.032 | -0.004 | 0.025 | -0.052 | -0.002 | 0.048 |
| 60.73 | -0.058 | -0.030 | -0.003 | 0.025 | -0.052 | -0.002 | 0.047 |
| 60.94 | -0.056 | -0.029 | -0.002 | 0.025 | -0.052 | -0.003 | 0.046 |
| 61.15 | -0.054 | -0.028 | -0.001 | 0.025 | -0.052 | -0.003 | 0.046 |
| 61.36 | -0.053 | -0.026 | 0.001 | 0.025 | -0.052 | -0.004 | 0.045 |
| 61.58 | -0.051 | -0.024 | 0.002 | 0.024 | -0.052 | -0.005 | 0.043 |
| 61.79 | -0.049 | -0.022 | 0.004 | 0.024 | -0.052 | -0.005 | 0.042 |
| 62.00 | -0.047 | -0.021 | 0.006 | 0.024 | -0.052 | -0.006 | 0.041 |
| 62.21 | -0.044 | -0.019 | 0.007 | 0.023 | -0.052 | -0.007 | 0.039 |
| 62.42 | -0.042 | -0.016 | 0.009 | 0.023 | -0.052 | -0.007 | 0.037 |
| 62.64 | -0.040 | -0.014 | 0.011 | 0.022 | -0.052 | -0.008 | 0.036 |
| 62.85 | -0.038 | -0.012 | 0.014 | 0.022 | -0.052 | -0.009 | 0.034 |
| 63.06 | -0.035 | -0.010 | 0.016 | 0.021 | -0.052 | -0.010 | 0.032 |
| 63.27 | -0.033 | -0.007 | 0.018 | 0.021 | -0.052 | -0.011 | 0.030 |
| 63.48 | -0.030 | -0.005 | 0.021 | 0.020 | -0.052 | -0.012 | 0.028 |
| 63.70 | -0.028 | -0.002 | 0.023 | 0.020 | -0.052 | -0.013 | 0.026 |
| 63.91 | -0.026 | 0.000 | 0.026 | 0.019 | -0.052 | -0.013 | 0.025 |
| 64.12 | -0.023 | 0.003 | 0.028 | 0.019 | -0.051 | -0.014 | 0.023 |
| 64.33 | -0.021 | 0.005 | 0.031 | 0.018 | -0.052 | -0.015 | 0.021 |
| 64.55 | -0.018 | 0.008 | 0.034 | 0.018 | -0.052 | -0.016 | 0.019 |
| 64.76 | -0.016 | 0.010 | 0.037 | 0.018 | -0.052 | -0.017 | 0.017 |
| 64.97 | -0.013 | 0.013 | 0.039 | 0.017 | -0.052 | -0.018 | 0.016 |
| 65.18 | -0.011 | 0.016 | 0.042 | 0.017 | -0.052 | -0.019 | 0.014 |
| 65.39 | -0.009 | 0.018 | 0.045 | 0.017 | -0.053 | -0.020 | 0.013 |
| 65.61 | -0.007 | 0.021 | 0.048 | 0.017 | -0.053 | -0.021 | 0.012 |
| 65.82 | -0.004 | 0.023 | 0.051 | 0.016 | -0.054 | -0.021 | 0.011 |
| 66.03 | -0.002 | 0.026 | 0.054 | 0.016 | -0.054 | -0.022 | 0.010 |
| 66.24 | 0.000 | 0.028 | 0.057 | 0.016 | -0.055 | -0.023 | 0.009 |
| 66.45 | 0.002 | 0.031 | 0.060 | 0.016 | -0.056 | -0.023 | 0.009 |
| 66.67 | 0.003 | 0.033 | 0.063 | 0.017 | -0.057 | -0.024 | 0.008 |
| 66.88 | 0.005 | 0.035 | 0.066 | 0.017 | -0.057 | -0.025 | 0.008 |
| 67.09 | 0.007 | 0.038 | 0.069 | 0.017 | -0.058 | -0.025 | 0.008 |
| 67.30 | 0.008 | 0.040 | 0.072 | 0.017 | -0.059 | -0.026 | 0.008 |
| **67.52** | **0.009** | **0.042** | **0.075** | **0.018** | **-0.060** | **-0.026** | **0.009** |
| **67.73** | **0.010** | **0.044** | **0.078** | **0.018** | **-0.061** | **-0.026** | **0.009** |
| **67.94** | **0.011** | **0.046** | **0.081** | **0.018** | **-0.062** | **-0.026** | **0.009** |
| **68.15** | **0.012** | **0.048** | **0.084** | **0.019** | **-0.063** | **-0.026** | **0.010** |
| **68.36** | **0.013** | **0.050** | **0.087** | **0.019** | **-0.064** | **-0.027** | **0.011** |
| **68.58** | **0.013** | **0.051** | **0.090** | **0.019** | **-0.064** | **-0.026** | **0.012** |
| **68.79** | **0.014** | **0.053** | **0.093** | **0.020** | **-0.065** | **-0.026** | **0.012** |
| **69.00** | **0.014** | **0.055** | **0.096** | **0.020** | **-0.065** | **-0.026** | **0.013** |
| 69.21 | 0.014 | 0.056 | 0.098 | 0.020 | -0.066 | -0.026 | 0.014 |
| 69.42 | 0.014 | 0.058 | 0.101 | 0.021 | -0.066 | -0.025 | 0.015 |
| 69.64 | 0.014 | 0.059 | 0.104 | 0.021 | -0.066 | -0.025 | 0.017 |
| 69.85 | 0.014 | 0.060 | 0.106 | 0.021 | -0.066 | -0.024 | 0.018 |
| 70.06 | 0.014 | 0.061 | 0.109 | 0.022 | -0.065 | -0.023 | 0.019 |
| 70.27 | 0.014 | 0.062 | 0.111 | 0.022 | -0.065 | -0.022 | 0.020 |
| 70.48 | 0.013 | 0.063 | 0.113 | 0.022 | -0.064 | -0.021 | 0.022 |
| 70.70 | 0.013 | 0.064 | 0.115 | 0.022 | -0.063 | -0.020 | 0.023 |
| 70.91 | 0.013 | 0.065 | 0.117 | 0.022 | -0.061 | -0.018 | 0.025 |
| 71.12 | 0.013 | 0.065 | 0.118 | 0.022 | -0.060 | -0.017 | 0.026 |
| 71.33 | 0.012 | 0.066 | 0.119 | 0.022 | -0.058 | -0.015 | 0.028 |
| 71.55 | 0.012 | 0.066 | 0.121 | 0.022 | -0.056 | -0.013 | 0.029 |
| 71.76 | 0.012 | 0.067 | 0.121 | 0.022 | -0.054 | -0.011 | 0.031 |
| 71.97 | 0.012 | 0.067 | 0.122 | 0.021 | -0.051 | -0.009 | 0.033 |
| 72.18 | 0.012 | 0.067 | 0.122 | 0.021 | -0.049 | -0.007 | 0.035 |
| 72.39 | 0.011 | 0.067 | 0.122 | 0.021 | -0.046 | -0.005 | 0.037 |
| 72.61 | 0.011 | 0.067 | 0.122 | 0.021 | -0.043 | -0.002 | 0.039 |
| 72.82 | 0.012 | 0.067 | 0.122 | 0.020 | -0.039 | 0.001 | 0.041 |
| 73.03 | 0.012 | 0.066 | 0.121 | 0.020 | -0.036 | 0.004 | 0.044 |
| 73.24 | 0.012 | 0.066 | 0.120 | 0.020 | -0.032 | 0.007 | 0.046 |
| 73.45 | 0.012 | 0.065 | 0.118 | 0.020 | -0.028 | 0.011 | 0.049 |
| 73.67 | 0.012 | 0.064 | 0.116 | 0.020 | -0.024 | 0.014 | 0.053 |
| 73.88 | 0.013 | 0.064 | 0.114 | 0.020 | -0.021 | 0.018 | 0.057 |
| 74.09 | 0.013 | 0.063 | 0.112 | 0.020 | -0.017 | 0.022 | 0.061 |
| 74.30 | 0.013 | 0.061 | 0.110 | 0.020 | -0.014 | 0.026 | 0.066 |
| 74.52 | 0.013 | 0.060 | 0.107 | 0.021 | -0.010 | 0.031 | 0.072 |
| 74.73 | 0.013 | 0.059 | 0.104 | 0.022 | -0.007 | 0.035 | 0.078 |
| 74.94 | 0.013 | 0.057 | 0.102 | 0.023 | -0.005 | 0.040 | 0.085 |
| 75.15 | 0.012 | 0.056 | 0.099 | 0.024 | -0.002 | 0.045 | 0.093 |
| 75.36 | 0.011 | 0.054 | 0.097 | 0.026 | 0.000 | 0.051 | 0.102 |
| 75.58 | 0.009 | 0.052 | 0.095 | 0.028 | 0.002 | 0.056 | 0.111 |
| 75.79 | 0.007 | 0.050 | 0.094 | 0.030 | 0.003 | 0.062 | 0.121 |
| 76.00 | 0.003 | 0.048 | 0.093 | 0.032 | 0.005 | 0.068 | 0.132 |

*Note.* $\Delta$DAN values represent the estimated mean differences for functional connectivity in the DAN between pre- and post-intervention for each group. The age range highlighted in bold indicates where significant differences between the two groups were found. Abbreviations: DAN = Dorsal attention network, SE = Standard error, CI = Confidence interval.

**Supplemental Table 4.1.a.** Age-varying relationships for change in FPCN FC with change in DSST performance.

| Age | ModVig_L  (Lower 95% CI) | Coefficients (*β*)  in ModVig | ModVig_U  (Upper 95% CI) | SE of  Association | Light_L  (Lower 95% CI) | Coefficients (*β*)  in Light | Light_U  (Upper 95% CI) |
| --- | --- | --- | --- | --- | --- | --- | --- |
| 55.00 | -0.037 | 0.037 | 0.110 | 0.053 | -0.196 | -0.093 | 0.010 |
| 55.21 | -0.033 | 0.040 | 0.113 | 0.050 | -0.195 | -0.096 | 0.003 |
| 55.42 | -0.029 | 0.043 | 0.116 | 0.048 | -0.193 | -0.098 | -0.003 |
| 55.64 | -0.025 | 0.047 | 0.119 | 0.047 | -0.192 | -0.100 | -0.009 |
| 55.85 | -0.022 | 0.050 | 0.122 | 0.045 | -0.191 | -0.102 | -0.014 |
| 56.06 | -0.019 | 0.053 | 0.125 | 0.044 | -0.189 | -0.104 | -0.018 |
| 56.27 | -0.017 | 0.055 | 0.128 | 0.042 | -0.188 | -0.105 | -0.022 |
| 56.48 | -0.014 | 0.058 | 0.131 | 0.041 | -0.187 | -0.106 | -0.025 |
| 56.70 | -0.012 | 0.061 | 0.134 | 0.040 | -0.187 | -0.107 | -0.028 |
| 56.91 | -0.010 | 0.063 | 0.137 | 0.040 | -0.186 | -0.108 | -0.031 |
| 57.12 | -0.008 | 0.066 | 0.139 | 0.039 | -0.185 | -0.109 | -0.032 |
| 57.33 | -0.007 | 0.068 | 0.142 | 0.038 | -0.184 | -0.109 | -0.034 |
| 57.55 | -0.005 | 0.070 | 0.145 | 0.038 | -0.183 | -0.109 | -0.035 |
| 57.76 | -0.004 | 0.072 | 0.147 | 0.037 | -0.183 | -0.109 | -0.036 |
| 57.97 | -0.002 | 0.074 | 0.150 | 0.037 | -0.182 | -0.109 | -0.036 |
| 58.18 | -0.001 | 0.076 | 0.152 | 0.037 | -0.181 | -0.109 | -0.036 |
| 58.39 | 0.000 | 0.077 | 0.155 | 0.037 | -0.180 | -0.108 | -0.036 |
| **58.61** | **0.001** | **0.079** | **0.157** | **0.036** | **-0.179** | **-0.107** | **-0.036** |
| **58.82** | **0.002** | **0.080** | **0.159** | **0.036** | **-0.178** | **-0.107** | **-0.036** |
| **59.03** | **0.002** | **0.082** | **0.161** | **0.036** | **-0.176** | **-0.106** | **-0.035** |
| **59.24** | **0.003** | **0.083** | **0.163** | **0.036** | **-0.175** | **-0.105** | **-0.034** |
| **59.45** | **0.004** | **0.084** | **0.164** | **0.036** | **-0.174** | **-0.103** | **-0.033** |
| **59.67** | **0.005** | **0.085** | **0.166** | **0.036** | **-0.172** | **-0.102** | **-0.032** |
| **59.88** | **0.005** | **0.086** | **0.167** | **0.036** | **-0.170** | **-0.101** | **-0.031** |
| **60.09** | **0.006** | **0.087** | **0.169** | **0.035** | **-0.169** | **-0.099** | **-0.030** |
| **60.30** | **0.006** | **0.088** | **0.170** | **0.035** | **-0.167** | **-0.098** | **-0.028** |
| **60.52** | **0.006** | **0.089** | **0.171** | **0.035** | **-0.165** | **-0.096** | **-0.027** |
| **60.73** | **0.007** | **0.090** | **0.172** | **0.035** | **-0.163** | **-0.094** | **-0.026** |
| **60.94** | **0.007** | **0.090** | **0.173** | **0.035** | **-0.161** | **-0.093** | **-0.024** |
| **61.15** | **0.007** | **0.091** | **0.174** | **0.035** | **-0.159** | **-0.091** | **-0.022** |
| **61.36** | **0.008** | **0.091** | **0.175** | **0.035** | **-0.157** | **-0.089** | **-0.021** |
| **61.58** | **0.008** | **0.092** | **0.175** | **0.035** | **-0.155** | **-0.087** | **-0.019** |
| **61.79** | **0.008** | **0.092** | **0.176** | **0.035** | **-0.153** | **-0.085** | **-0.017** |
| **62.00** | **0.008** | **0.092** | **0.176** | **0.035** | **-0.151** | **-0.083** | **-0.015** |
| **62.21** | **0.008** | **0.092** | **0.177** | **0.035** | **-0.149** | **-0.081** | **-0.013** |
| **62.42** | **0.008** | **0.093** | **0.177** | **0.035** | **-0.147** | **-0.079** | **-0.011** |
| **62.64** | **0.008** | **0.093** | **0.177** | **0.035** | **-0.145** | **-0.077** | **-0.009** |
| **62.85** | **0.008** | **0.093** | **0.178** | **0.035** | **-0.143** | **-0.075** | **-0.007** |
| **63.06** | **0.008** | **0.093** | **0.178** | **0.035** | **-0.142** | **-0.073** | **-0.005** |
| **63.27** | **0.008** | **0.093** | **0.178** | **0.035** | **-0.140** | **-0.072** | **-0.003** |
| **63.48** | **0.008** | **0.093** | **0.178** | **0.035** | **-0.139** | **-0.070** | **-0.001** |
| **63.70** | **0.007** | **0.092** | **0.177** | **0.035** | **-0.137** | **-0.068** | **0.001** |
| **63.91** | **0.007** | **0.092** | **0.177** | **0.036** | **-0.136** | **-0.066** | **0.003** |
| **64.12** | **0.007** | **0.092** | **0.177** | **0.036** | **-0.135** | **-0.065** | **0.006** |
| 64.33 | 0.007 | 0.092 | 0.177 | 0.036 | -0.134 | -0.063 | 0.008 |
| 64.55 | 0.006 | 0.091 | 0.176 | 0.036 | -0.133 | -0.061 | 0.010 |
| 64.76 | 0.006 | 0.091 | 0.176 | 0.037 | -0.132 | -0.060 | 0.012 |
| 64.97 | 0.005 | 0.091 | 0.176 | 0.037 | -0.131 | -0.059 | 0.014 |
| 65.18 | 0.005 | 0.090 | 0.175 | 0.037 | -0.131 | -0.057 | 0.016 |
| 65.39 | 0.005 | 0.090 | 0.175 | 0.038 | -0.131 | -0.056 | 0.018 |
| 65.61 | 0.004 | 0.089 | 0.174 | 0.038 | -0.130 | -0.055 | 0.020 |
| 65.82 | 0.004 | 0.089 | 0.174 | 0.039 | -0.130 | -0.054 | 0.022 |
| 66.03 | 0.003 | 0.088 | 0.173 | 0.039 | -0.131 | -0.054 | 0.024 |
| 66.24 | 0.003 | 0.088 | 0.173 | 0.040 | -0.131 | -0.053 | 0.025 |
| 66.45 | 0.002 | 0.087 | 0.172 | 0.040 | -0.132 | -0.053 | 0.027 |
| 66.67 | 0.002 | 0.087 | 0.171 | 0.041 | -0.132 | -0.052 | 0.028 |
| 66.88 | 0.001 | 0.086 | 0.171 | 0.041 | -0.133 | -0.052 | 0.029 |
| 67.09 | 0.001 | 0.085 | 0.170 | 0.042 | -0.134 | -0.052 | 0.030 |
| 67.30 | 0.000 | 0.085 | 0.169 | 0.042 | -0.136 | -0.052 | 0.031 |
| 67.52 | 0.000 | 0.084 | 0.169 | 0.043 | -0.137 | -0.053 | 0.031 |
| 67.73 | -0.001 | 0.084 | 0.168 | 0.044 | -0.139 | -0.053 | 0.032 |
| 67.94 | -0.001 | 0.083 | 0.167 | 0.044 | -0.141 | -0.054 | 0.032 |
| 68.15 | -0.002 | 0.082 | 0.167 | 0.045 | -0.143 | -0.055 | 0.032 |
| 68.36 | -0.002 | 0.082 | 0.166 | 0.045 | -0.145 | -0.057 | 0.032 |
| 68.58 | -0.003 | 0.081 | 0.165 | 0.046 | -0.148 | -0.058 | 0.031 |
| 68.79 | -0.003 | 0.081 | 0.164 | 0.046 | -0.150 | -0.060 | 0.030 |
| 69.00 | -0.004 | 0.080 | 0.164 | 0.047 | -0.153 | -0.062 | 0.029 |
| 69.21 | -0.004 | 0.079 | 0.163 | 0.047 | -0.156 | -0.064 | 0.028 |
| 69.42 | -0.004 | 0.079 | 0.162 | 0.047 | -0.160 | -0.067 | 0.026 |
| 69.64 | -0.005 | 0.078 | 0.161 | 0.048 | -0.163 | -0.070 | 0.024 |
| 69.85 | -0.005 | 0.078 | 0.161 | 0.048 | -0.167 | -0.073 | 0.022 |
| 70.06 | -0.005 | 0.077 | 0.160 | 0.049 | -0.172 | -0.076 | 0.020 |
| 70.27 | -0.006 | 0.077 | 0.159 | 0.049 | -0.176 | -0.080 | 0.017 |
| 70.48 | -0.006 | 0.076 | 0.158 | 0.050 | -0.181 | -0.084 | 0.014 |
| 70.70 | -0.006 | 0.076 | 0.158 | 0.050 | -0.186 | -0.088 | 0.011 |
| 70.91 | -0.006 | 0.076 | 0.157 | 0.051 | -0.192 | -0.092 | 0.007 |
| 71.12 | -0.006 | 0.075 | 0.156 | 0.051 | -0.198 | -0.097 | 0.003 |
| 71.33 | -0.006 | 0.075 | 0.155 | 0.052 | -0.204 | -0.103 | -0.001 |
| 71.55 | -0.006 | 0.075 | 0.155 | 0.053 | -0.211 | -0.108 | -0.005 |
| 71.76 | -0.005 | 0.074 | 0.154 | 0.053 | -0.219 | -0.114 | -0.010 |
| 71.97 | -0.005 | 0.074 | 0.153 | 0.054 | -0.226 | -0.121 | -0.015 |
| 72.18 | -0.005 | 0.074 | 0.153 | 0.055 | -0.235 | -0.127 | -0.020 |
| 72.39 | -0.005 | 0.074 | 0.152 | 0.056 | -0.244 | -0.134 | -0.025 |
| 72.61 | -0.004 | 0.074 | 0.152 | 0.057 | -0.253 | -0.142 | -0.031 |
| 72.82 | -0.004 | 0.074 | 0.151 | 0.058 | -0.263 | -0.150 | -0.036 |
| 73.03 | -0.003 | 0.074 | 0.151 | 0.059 | -0.274 | -0.158 | -0.042 |
| 73.24 | -0.002 | 0.074 | 0.150 | 0.061 | -0.286 | -0.167 | -0.048 |
| 73.45 | -0.002 | 0.074 | 0.150 | 0.062 | -0.298 | -0.176 | -0.053 |
| 73.67 | -0.001 | 0.074 | 0.149 | 0.064 | -0.311 | -0.185 | -0.059 |
| 73.88 | 0.000 | 0.075 | 0.149 | 0.066 | -0.325 | -0.195 | -0.065 |
| 74.09 | 0.001 | 0.075 | 0.149 | 0.069 | -0.340 | -0.206 | -0.071 |
| 74.30 | 0.002 | 0.075 | 0.149 | 0.071 | -0.356 | -0.217 | -0.077 |
| 74.52 | 0.003 | 0.076 | 0.149 | 0.074 | -0.373 | -0.228 | -0.083 |
| 74.73 | 0.004 | 0.076 | 0.149 | 0.077 | -0.390 | -0.240 | -0.089 |
| 74.94 | 0.004 | 0.077 | 0.149 | 0.080 | -0.409 | -0.252 | -0.095 |
| 75.15 | 0.005 | 0.078 | 0.150 | 0.083 | -0.428 | -0.265 | -0.101 |
| 75.36 | 0.006 | 0.078 | 0.150 | 0.087 | -0.449 | -0.278 | -0.107 |
| 75.58 | 0.007 | 0.079 | 0.151 | 0.091 | -0.470 | -0.292 | -0.113 |
| 75.79 | 0.008 | 0.080 | 0.152 | 0.095 | -0.493 | -0.306 | -0.119 |
| 76.00 | 0.009 | 0.081 | 0.153 | 0.100 | -0.516 | -0.321 | -0.125 |

*Note.* Coefficients (*β*) represent the estimated magnitude of the associations between changes in FPCN FC and changes in DSST performance for each group. The age range highlighted in bold indicates where significant differences between the two groups were found. Abbreviations: FPCN = Frontoparietal control network, DSST = Digit symbol substitution test, SE = Standard error, CI = Confidence interval.

**Supplemental Table 4.2.a.** Age-varying relationships for change in DMN FC with change in DSST performance.

| Age | ModVig_L  (Lower 95% CI) | Coefficients (*β*)  in ModVig | ModVig_U  (Upper 95% CI) | SE of  Association | Light_L  (Lower 95% CI) | Coefficients (*β*)  in Light | Light_U  (Upper 95% CI) |
| --- | --- | --- | --- | --- | --- | --- | --- |
| 55.00 | -0.045 | 0.044 | 0.133 | 0.038 | -0.118 | -0.042 | 0.033 |
| 55.21 | -0.040 | 0.045 | 0.130 | 0.037 | -0.114 | -0.041 | 0.032 |
| 55.42 | -0.035 | 0.046 | 0.127 | 0.037 | -0.111 | -0.039 | 0.032 |
| 55.64 | -0.031 | 0.047 | 0.125 | 0.036 | -0.108 | -0.038 | 0.032 |
| 55.85 | -0.027 | 0.048 | 0.123 | 0.036 | -0.106 | -0.037 | 0.033 |
| 56.06 | -0.023 | 0.050 | 0.122 | 0.035 | -0.105 | -0.036 | 0.034 |
| 56.27 | -0.019 | 0.051 | 0.121 | 0.035 | -0.104 | -0.035 | 0.034 |
| 56.48 | -0.016 | 0.052 | 0.120 | 0.035 | -0.103 | -0.034 | 0.035 |
| 56.70 | -0.013 | 0.054 | 0.120 | 0.035 | -0.102 | -0.033 | 0.036 |
| 56.91 | -0.010 | 0.055 | 0.120 | 0.035 | -0.102 | -0.032 | 0.037 |
| 57.12 | -0.007 | 0.057 | 0.120 | 0.036 | -0.101 | -0.031 | 0.038 |
| 57.33 | -0.005 | 0.058 | 0.121 | 0.036 | -0.101 | -0.031 | 0.039 |
| 57.55 | -0.002 | 0.060 | 0.122 | 0.036 | -0.101 | -0.030 | 0.040 |
| 57.76 | 0.000 | 0.061 | 0.123 | 0.036 | -0.101 | -0.030 | 0.041 |
| 57.97 | 0.002 | 0.063 | 0.124 | 0.037 | -0.101 | -0.030 | 0.042 |
| 58.18 | 0.004 | 0.065 | 0.125 | 0.037 | -0.101 | -0.029 | 0.043 |
| 58.39 | 0.006 | 0.066 | 0.127 | 0.037 | -0.102 | -0.029 | 0.043 |
| 58.61 | 0.008 | 0.068 | 0.129 | 0.037 | -0.102 | -0.029 | 0.043 |
| 58.82 | 0.009 | 0.070 | 0.131 | 0.037 | -0.102 | -0.029 | 0.044 |
| 59.03 | 0.011 | 0.072 | 0.132 | 0.037 | -0.102 | -0.029 | 0.044 |
| 59.24 | 0.013 | 0.073 | 0.134 | 0.037 | -0.102 | -0.029 | 0.044 |
| 59.45 | 0.014 | 0.075 | 0.136 | 0.037 | -0.103 | -0.029 | 0.044 |
| 59.67 | 0.016 | 0.077 | 0.139 | 0.037 | -0.103 | -0.030 | 0.043 |
| 59.88 | 0.017 | 0.079 | 0.141 | 0.037 | -0.103 | -0.030 | 0.043 |
| 60.09 | 0.019 | 0.081 | 0.143 | 0.037 | -0.103 | -0.030 | 0.043 |
| 60.30 | 0.020 | 0.083 | 0.145 | 0.037 | -0.103 | -0.031 | 0.042 |
| 60.52 | 0.022 | 0.084 | 0.147 | 0.037 | -0.103 | -0.031 | 0.041 |
| 60.73 | 0.023 | 0.086 | 0.149 | 0.037 | -0.103 | -0.031 | 0.041 |
| 60.94 | 0.025 | 0.088 | 0.152 | 0.037 | -0.103 | -0.032 | 0.040 |
| 61.15 | 0.026 | 0.090 | 0.154 | 0.036 | -0.103 | -0.032 | 0.039 |
| 61.36 | 0.028 | 0.092 | 0.156 | 0.036 | -0.103 | -0.033 | 0.038 |
| 61.58 | 0.029 | 0.094 | 0.158 | 0.036 | -0.103 | -0.033 | 0.037 |
| 61.79 | 0.031 | 0.095 | 0.160 | 0.036 | -0.104 | -0.034 | 0.036 |
| 62.00 | 0.032 | 0.097 | 0.162 | 0.035 | -0.104 | -0.034 | 0.035 |
| 62.21 | 0.034 | 0.099 | 0.164 | 0.035 | -0.104 | -0.035 | 0.034 |
| **62.42** | **0.035** | **0.101** | **0.166** | **0.035** | **-0.104** | **-0.035** | **0.033** |
| **62.64** | **0.037** | **0.103** | **0.169** | **0.035** | **-0.104** | **-0.036** | **0.032** |
| **62.85** | **0.038** | **0.104** | **0.171** | **0.035** | **-0.104** | **-0.036** | **0.032** |
| **63.06** | **0.039** | **0.106** | **0.173** | **0.034** | **-0.104** | **-0.037** | **0.031** |
| **63.27** | **0.041** | **0.108** | **0.175** | **0.034** | **-0.105** | **-0.037** | **0.030** |
| **63.48** | **0.042** | **0.109** | **0.177** | **0.034** | **-0.105** | **-0.038** | **0.029** |
| **63.70** | **0.043** | **0.111** | **0.178** | **0.034** | **-0.105** | **-0.038** | **0.028** |
| **63.91** | **0.045** | **0.112** | **0.180** | **0.034** | **-0.106** | **-0.039** | **0.028** |
| **64.12** | **0.046** | **0.114** | **0.182** | **0.034** | **-0.106** | **-0.039** | **0.027** |
| **64.33** | **0.047** | **0.116** | **0.184** | **0.034** | **-0.106** | **-0.040** | **0.027** |
| **64.55** | **0.048** | **0.117** | **0.186** | **0.034** | **-0.107** | **-0.040** | **0.027** |
| **64.76** | **0.049** | **0.118** | **0.188** | **0.034** | **-0.107** | **-0.040** | **0.026** |
| **64.97** | **0.050** | **0.120** | **0.189** | **0.034** | **-0.108** | **-0.041** | **0.026** |
| **65.18** | **0.051** | **0.121** | **0.191** | **0.034** | **-0.108** | **-0.041** | **0.026** |
| **65.39** | **0.052** | **0.122** | **0.193** | **0.034** | **-0.109** | **-0.041** | **0.026** |
| **65.61** | **0.052** | **0.123** | **0.194** | **0.035** | **-0.109** | **-0.041** | **0.026** |
| **65.82** | **0.053** | **0.125** | **0.196** | **0.035** | **-0.110** | **-0.042** | **0.027** |
| **66.03** | **0.054** | **0.126** | **0.197** | **0.035** | **-0.110** | **-0.042** | **0.027** |
| **66.24** | **0.054** | **0.127** | **0.199** | **0.035** | **-0.111** | **-0.042** | **0.028** |
| **66.45** | **0.055** | **0.127** | **0.200** | **0.036** | **-0.111** | **-0.042** | **0.028** |
| **66.67** | **0.055** | **0.128** | **0.202** | **0.036** | **-0.112** | **-0.041** | **0.029** |
| **66.88** | **0.055** | **0.129** | **0.203** | **0.036** | **-0.112** | **-0.041** | **0.029** |
| **67.09** | **0.056** | **0.130** | **0.204** | **0.036** | **-0.112** | **-0.041** | **0.030** |
| **67.30** | **0.056** | **0.130** | **0.205** | **0.037** | **-0.113** | **-0.041** | **0.031** |
| **67.52** | **0.056** | **0.131** | **0.206** | **0.037** | **-0.113** | **-0.040** | **0.032** |
| **67.73** | **0.056** | **0.132** | **0.207** | **0.037** | **-0.113** | **-0.040** | **0.033** |
| **67.94** | **0.056** | **0.132** | **0.208** | **0.037** | **-0.113** | **-0.039** | **0.034** |
| **68.15** | **0.056** | **0.132** | **0.209** | **0.038** | **-0.112** | **-0.039** | **0.035** |
| **68.36** | **0.055** | **0.132** | **0.209** | **0.038** | **-0.112** | **-0.038** | **0.037** |
| **68.58** | **0.055** | **0.133** | **0.210** | **0.038** | **-0.112** | **-0.037** | **0.038** |
| **68.79** | **0.055** | **0.133** | **0.210** | **0.038** | **-0.111** | **-0.036** | **0.039** |
| **69.00** | **0.054** | **0.132** | **0.211** | **0.039** | **-0.110** | **-0.035** | **0.041** |
| **69.21** | **0.054** | **0.132** | **0.211** | **0.039** | **-0.110** | **-0.034** | **0.042** |
| **69.42** | **0.053** | **0.132** | **0.211** | **0.039** | **-0.109** | **-0.032** | **0.044** |
| **69.64** | **0.053** | **0.132** | **0.211** | **0.039** | **-0.107** | **-0.031** | **0.046** |
| **69.85** | **0.052** | **0.131** | **0.210** | **0.039** | **-0.106** | **-0.029** | **0.047** |
| **70.06** | **0.051** | **0.131** | **0.210** | **0.039** | **-0.105** | **-0.028** | **0.049** |
| 70.27 | 0.050 | 0.130 | 0.209 | 0.039 | -0.103 | -0.026 | 0.051 |
| 70.48 | 0.050 | 0.129 | 0.209 | 0.039 | -0.101 | -0.024 | 0.053 |
| 70.70 | 0.049 | 0.128 | 0.208 | 0.040 | -0.099 | -0.022 | 0.056 |
| 70.91 | 0.048 | 0.127 | 0.206 | 0.040 | -0.098 | -0.020 | 0.058 |
| 71.12 | 0.047 | 0.126 | 0.205 | 0.040 | -0.095 | -0.017 | 0.061 |
| 71.33 | 0.046 | 0.125 | 0.204 | 0.040 | -0.093 | -0.015 | 0.063 |
| 71.55 | 0.044 | 0.123 | 0.202 | 0.040 | -0.091 | -0.012 | 0.067 |
| 71.76 | 0.043 | 0.122 | 0.200 | 0.041 | -0.089 | -0.010 | 0.070 |
| 71.97 | 0.042 | 0.120 | 0.198 | 0.041 | -0.087 | -0.007 | 0.074 |
| 72.18 | 0.040 | 0.118 | 0.196 | 0.041 | -0.085 | -0.004 | 0.078 |
| 72.39 | 0.039 | 0.116 | 0.193 | 0.042 | -0.083 | 0.000 | 0.082 |
| 72.61 | 0.037 | 0.114 | 0.191 | 0.043 | -0.080 | 0.003 | 0.087 |
| 72.82 | 0.036 | 0.112 | 0.188 | 0.044 | -0.079 | 0.007 | 0.092 |
| 73.03 | 0.034 | 0.109 | 0.185 | 0.045 | -0.077 | 0.010 | 0.098 |
| 73.24 | 0.032 | 0.107 | 0.181 | 0.046 | -0.075 | 0.014 | 0.104 |
| 73.45 | 0.031 | 0.104 | 0.178 | 0.047 | -0.074 | 0.019 | 0.111 |
| 73.67 | 0.029 | 0.101 | 0.174 | 0.049 | -0.073 | 0.023 | 0.119 |
| 73.88 | 0.027 | 0.098 | 0.170 | 0.051 | -0.072 | 0.027 | 0.127 |
| 74.09 | 0.024 | 0.095 | 0.166 | 0.053 | -0.072 | 0.032 | 0.136 |
| 74.30 | 0.022 | 0.092 | 0.162 | 0.055 | -0.071 | 0.037 | 0.145 |
| 74.52 | 0.020 | 0.088 | 0.157 | 0.058 | -0.071 | 0.042 | 0.155 |
| 74.73 | 0.017 | 0.085 | 0.153 | 0.061 | -0.072 | 0.047 | 0.166 |
| 74.94 | 0.014 | 0.081 | 0.148 | 0.064 | -0.073 | 0.053 | 0.178 |
| 75.15 | 0.011 | 0.077 | 0.143 | 0.067 | -0.074 | 0.058 | 0.190 |
| 75.36 | 0.007 | 0.073 | 0.138 | 0.071 | -0.075 | 0.064 | 0.204 |
| 75.58 | 0.004 | 0.068 | 0.133 | 0.075 | -0.077 | 0.070 | 0.218 |
| 75.79 | 0.000 | 0.064 | 0.128 | 0.079 | -0.079 | 0.077 | 0.232 |
| 76.00 | -0.005 | 0.059 | 0.124 | 0.084 | -0.081 | 0.083 | 0.248 |

*Note.* Coefficients (*β*) represent the estimated magnitude of the associations between changes in DMN FC and changes in DSST performance for each group. The age range highlighted in bold indicates where significant differences between the two groups were found. Abbreviations: DMN = Default mode network, DSST = Digit symbol substitution test, SE = Standard error, CI = Confidence interval.

**Supplemental Table 4.2.b.** Age-varying relationships for change in DMN FC with change in Pattern Comparison performance.

| Age | ModVig_L  (Lower 95% CI) | Coefficients (*β*)  in ModVig | ModVig_U  (Upper 95% CI) | SE of  Association | Light_L  (Lower 95% CI) | Coefficients (*β*)  in Light | Light_U  (Upper 95% CI) |
| --- | --- | --- | --- | --- | --- | --- | --- |
| 55.00 | -0.005 | 0.084 | 0.172 | 0.029 | -0.073 | -0.015 | 0.043 |
| 55.21 | -0.004 | 0.080 | 0.165 | 0.029 | -0.072 | -0.016 | 0.040 |
| 55.42 | -0.004 | 0.077 | 0.158 | 0.028 | -0.072 | -0.017 | 0.038 |
| 55.64 | -0.003 | 0.074 | 0.152 | 0.028 | -0.072 | -0.018 | 0.037 |
| 55.85 | -0.003 | 0.072 | 0.146 | 0.027 | -0.072 | -0.018 | 0.035 |
| 56.06 | -0.003 | 0.069 | 0.141 | 0.027 | -0.072 | -0.019 | 0.034 |
| 56.27 | -0.002 | 0.067 | 0.136 | 0.027 | -0.073 | -0.020 | 0.034 |
| 56.48 | -0.002 | 0.065 | 0.132 | 0.027 | -0.073 | -0.020 | 0.033 |
| 56.70 | -0.002 | 0.063 | 0.128 | 0.027 | -0.074 | -0.020 | 0.033 |
| 56.91 | -0.002 | 0.061 | 0.124 | 0.027 | -0.075 | -0.021 | 0.033 |
| 57.12 | -0.002 | 0.060 | 0.122 | 0.028 | -0.075 | -0.021 | 0.033 |
| 57.33 | -0.001 | 0.059 | 0.119 | 0.028 | -0.076 | -0.021 | 0.033 |
| 57.55 | -0.001 | 0.058 | 0.117 | 0.028 | -0.077 | -0.021 | 0.034 |
| 57.76 | -0.001 | 0.057 | 0.115 | 0.028 | -0.077 | -0.022 | 0.034 |
| 57.97 | -0.001 | 0.056 | 0.113 | 0.029 | -0.078 | -0.022 | 0.035 |
| 58.18 | 0.000 | 0.056 | 0.112 | 0.029 | -0.079 | -0.022 | 0.035 |
| 58.39 | 0.000 | 0.056 | 0.111 | 0.029 | -0.079 | -0.022 | 0.036 |
| 58.61 | 0.000 | 0.056 | 0.111 | 0.030 | -0.080 | -0.022 | 0.037 |
| 58.82 | 0.001 | 0.056 | 0.110 | 0.030 | -0.080 | -0.022 | 0.037 |
| 59.03 | 0.001 | 0.056 | 0.110 | 0.030 | -0.081 | -0.021 | 0.038 |
| 59.24 | 0.002 | 0.056 | 0.110 | 0.031 | -0.081 | -0.021 | 0.039 |
| 59.45 | 0.003 | 0.057 | 0.111 | 0.031 | -0.082 | -0.021 | 0.039 |
| 59.67 | 0.004 | 0.057 | 0.111 | 0.031 | -0.082 | -0.021 | 0.040 |
| 59.88 | 0.004 | 0.058 | 0.112 | 0.031 | -0.082 | -0.021 | 0.041 |
| 60.09 | 0.005 | 0.059 | 0.113 | 0.032 | -0.083 | -0.021 | 0.041 |
| 60.30 | 0.006 | 0.060 | 0.114 | 0.032 | -0.083 | -0.021 | 0.042 |
| 60.52 | 0.008 | 0.061 | 0.115 | 0.032 | -0.083 | -0.020 | 0.043 |
| 60.73 | 0.009 | 0.062 | 0.116 | 0.032 | -0.084 | -0.020 | 0.043 |
| 60.94 | 0.010 | 0.064 | 0.117 | 0.033 | -0.084 | -0.020 | 0.044 |
| 61.15 | 0.011 | 0.065 | 0.119 | 0.033 | -0.084 | -0.020 | 0.044 |
| 61.36 | 0.013 | 0.067 | 0.121 | 0.033 | -0.084 | -0.020 | 0.045 |
| 61.58 | 0.014 | 0.068 | 0.122 | 0.033 | -0.084 | -0.020 | 0.045 |
| 61.79 | 0.016 | 0.070 | 0.124 | 0.033 | -0.085 | -0.019 | 0.046 |
| 62.00 | 0.017 | 0.072 | 0.126 | 0.033 | -0.085 | -0.019 | 0.046 |
| 62.21 | 0.019 | 0.073 | 0.128 | 0.034 | -0.085 | -0.019 | 0.047 |
| 62.42 | 0.020 | 0.075 | 0.130 | 0.034 | -0.085 | -0.019 | 0.047 |
| 62.64 | 0.022 | 0.077 | 0.133 | 0.034 | -0.086 | -0.019 | 0.048 |
| 62.85 | 0.024 | 0.079 | 0.135 | 0.034 | -0.086 | -0.019 | 0.048 |
| 63.06 | 0.025 | 0.081 | 0.137 | 0.034 | -0.086 | -0.019 | 0.048 |
| 63.27 | 0.027 | 0.083 | 0.140 | 0.034 | -0.086 | -0.019 | 0.048 |
| 63.48 | 0.028 | 0.085 | 0.142 | 0.035 | -0.087 | -0.019 | 0.049 |
| 63.70 | 0.030 | 0.087 | 0.145 | 0.035 | -0.087 | -0.019 | 0.049 |
| 63.91 | 0.032 | 0.090 | 0.148 | 0.035 | -0.087 | -0.019 | 0.049 |
| 64.12 | 0.033 | 0.092 | 0.150 | 0.035 | -0.088 | -0.019 | 0.049 |
| 64.33 | 0.035 | 0.094 | 0.153 | 0.035 | -0.088 | -0.020 | 0.049 |
| 64.55 | 0.036 | 0.096 | 0.156 | 0.035 | -0.089 | -0.020 | 0.049 |
| 64.76 | 0.038 | 0.098 | 0.159 | 0.035 | -0.089 | -0.020 | 0.049 |
| 64.97 | 0.039 | 0.100 | 0.161 | 0.035 | -0.090 | -0.020 | 0.049 |
| 65.18 | 0.040 | 0.102 | 0.164 | 0.035 | -0.090 | -0.021 | 0.049 |
| 65.39 | 0.041 | 0.104 | 0.167 | 0.035 | -0.091 | -0.021 | 0.048 |
| 65.61 | 0.043 | 0.106 | 0.170 | 0.035 | -0.091 | -0.022 | 0.048 |
| 65.82 | 0.044 | 0.108 | 0.173 | 0.036 | -0.092 | -0.022 | 0.048 |
| 66.03 | 0.045 | 0.110 | 0.176 | 0.036 | -0.092 | -0.023 | 0.047 |
| 66.24 | 0.046 | 0.112 | 0.179 | 0.036 | -0.093 | -0.023 | 0.047 |
| **66.45** | **0.047** | **0.114** | **0.181** | **0.036** | **-0.093** | **-0.024** | **0.046** |
| **66.67** | **0.048** | **0.116** | **0.184** | **0.036** | **-0.094** | **-0.024** | **0.046** |
| **66.88** | **0.049** | **0.118** | **0.187** | **0.036** | **-0.095** | **-0.025** | **0.045** |
| **67.09** | **0.049** | **0.119** | **0.189** | **0.036** | **-0.095** | **-0.025** | **0.044** |
| **67.30** | **0.050** | **0.121** | **0.192** | **0.036** | **-0.096** | **-0.026** | **0.044** |
| **67.52** | **0.050** | **0.122** | **0.194** | **0.035** | **-0.096** | **-0.027** | **0.043** |
| **67.73** | **0.051** | **0.124** | **0.197** | **0.035** | **-0.097** | **-0.027** | **0.042** |
| **67.94** | **0.051** | **0.125** | **0.199** | **0.035** | **-0.097** | **-0.028** | **0.041** |
| **68.15** | **0.052** | **0.126** | **0.201** | **0.035** | **-0.098** | **-0.029** | **0.040** |
| **68.36** | **0.052** | **0.128** | **0.203** | **0.035** | **-0.098** | **-0.030** | **0.039** |
| **68.58** | **0.052** | **0.129** | **0.205** | **0.035** | **-0.099** | **-0.030** | **0.038** |
| **68.79** | **0.052** | **0.129** | **0.207** | **0.035** | **-0.099** | **-0.031** | **0.037** |
| **69.00** | **0.052** | **0.130** | **0.208** | **0.034** | **-0.099** | **-0.032** | **0.036** |
| **69.21** | **0.052** | **0.131** | **0.210** | **0.034** | **-0.100** | **-0.033** | **0.034** |
| **69.42** | **0.052** | **0.131** | **0.211** | **0.034** | **-0.100** | **-0.034** | **0.033** |
| **69.64** | **0.051** | **0.132** | **0.212** | **0.034** | **-0.100** | **-0.034** | **0.032** |
| **69.85** | **0.051** | **0.132** | **0.213** | **0.033** | **-0.101** | **-0.035** | **0.030** |
| **70.06** | **0.051** | **0.132** | **0.213** | **0.033** | **-0.101** | **-0.036** | **0.029** |
| **70.27** | **0.050** | **0.132** | **0.213** | **0.033** | **-0.101** | **-0.037** | **0.027** |
| **70.48** | **0.049** | **0.131** | **0.214** | **0.032** | **-0.101** | **-0.038** | **0.026** |
| **70.70** | **0.049** | **0.131** | **0.213** | **0.032** | **-0.101** | **-0.038** | **0.024** |
| **70.91** | **0.048** | **0.130** | **0.213** | **0.031** | **-0.101** | **-0.039** | **0.023** |
| **71.12** | **0.047** | **0.130** | **0.212** | **0.031** | **-0.101** | **-0.040** | **0.021** |
| **71.33** | **0.046** | **0.129** | **0.211** | **0.031** | **-0.100** | **-0.041** | **0.019** |
| **71.55** | **0.045** | **0.127** | **0.210** | **0.030** | **-0.100** | **-0.041** | **0.017** |
| **71.76** | **0.043** | **0.126** | **0.209** | **0.029** | **-0.100** | **-0.042** | **0.016** |
| **71.97** | **0.042** | **0.124** | **0.207** | **0.029** | **-0.099** | **-0.043** | **0.014** |
| **72.18** | **0.041** | **0.123** | **0.205** | **0.028** | **-0.099** | **-0.043** | **0.012** |
| **72.39** | **0.039** | **0.121** | **0.202** | **0.028** | **-0.098** | **-0.044** | **0.010** |
| **72.61** | **0.037** | **0.118** | **0.199** | **0.027** | **-0.097** | **-0.045** | **0.008** |
| **72.82** | **0.035** | **0.116** | **0.196** | **0.026** | **-0.097** | **-0.045** | **0.006** |
| **73.03** | **0.033** | **0.113** | **0.193** | **0.026** | **-0.096** | **-0.046** | **0.005** |
| **73.24** | **0.031** | **0.110** | **0.189** | **0.025** | **-0.095** | **-0.046** | **0.003** |
| **73.45** | **0.029** | **0.107** | **0.185** | **0.024** | **-0.094** | **-0.046** | **0.001** |
| **73.67** | **0.027** | **0.103** | **0.180** | **0.023** | **-0.093** | **-0.047** | **-0.001** |
| **73.88** | **0.024** | **0.100** | **0.175** | **0.023** | **-0.092** | **-0.047** | **-0.003** |
| **74.09** | **0.021** | **0.096** | **0.170** | **0.022** | **-0.091** | **-0.048** | **-0.005** |
| **74.30** | **0.018** | **0.091** | **0.164** | **0.021** | **-0.089** | **-0.048** | **-0.006** |
| **74.52** | **0.015** | **0.087** | **0.158** | **0.021** | **-0.088** | **-0.048** | **-0.008** |
| **74.73** | **0.012** | **0.082** | **0.152** | **0.020** | **-0.087** | **-0.048** | **-0.009** |
| **74.94** | **0.008** | **0.077** | **0.146** | **0.019** | **-0.086** | **-0.048** | **-0.010** |
| **75.15** | **0.004** | **0.071** | **0.139** | **0.019** | **-0.085** | **-0.048** | **-0.011** |
| 75.36 | 0.000 | 0.066 | 0.131 | 0.019 | -0.085 | -0.048 | -0.012 |
| 75.58 | -0.005 | 0.060 | 0.124 | 0.018 | -0.084 | -0.048 | -0.012 |
| 75.79 | -0.010 | 0.053 | 0.116 | 0.018 | -0.084 | -0.048 | -0.012 |
| 76.00 | -0.015 | 0.046 | 0.108 | 0.018 | -0.084 | -0.048 | -0.012 |

*Note.* Coefficients (*β*) represent the estimated magnitude of the associations between changes in DMN FC and changes in pattern comparison performance for each group. The age range highlighted in bold indicates where significant differences between the two groups were found. Abbreviations: DMN = Default mode network, SE = Standard error, CI = Confidence interval.

**Supplemental Table 4.2.c.** Age-varying relationships for change in DMN FC with change in Letter Comparison performance.

| Age | ModVig_L  (Lower 95% CI) | Coefficients (*β*)  in ModVig | ModVig_U  (Upper 95% CI) | SE of  Association | Light_L  (Lower 95% CI) | Coefficients (*β*)  in Light | Light_U  (Upper 95% CI) |
| --- | --- | --- | --- | --- | --- | --- | --- |
| 55.00 | -0.029 | 0.058 | 0.145 | 0.036 | -0.120 | -0.050 | 0.020 |
| 55.21 | -0.027 | 0.056 | 0.139 | 0.034 | -0.115 | -0.048 | 0.018 |
| 55.42 | -0.026 | 0.053 | 0.133 | 0.033 | -0.111 | -0.047 | 0.017 |
| 55.64 | -0.025 | 0.051 | 0.128 | 0.032 | -0.108 | -0.046 | 0.016 |
| 55.85 | -0.023 | 0.050 | 0.123 | 0.031 | -0.105 | -0.045 | 0.016 |
| 56.06 | -0.022 | 0.048 | 0.118 | 0.030 | -0.103 | -0.044 | 0.016 |
| 56.27 | -0.021 | 0.046 | 0.114 | 0.030 | -0.101 | -0.042 | 0.016 |
| 56.48 | -0.020 | 0.045 | 0.110 | 0.030 | -0.100 | -0.041 | 0.017 |
| 56.70 | -0.019 | 0.044 | 0.107 | 0.030 | -0.099 | -0.040 | 0.018 |
| 56.91 | -0.018 | 0.043 | 0.104 | 0.030 | -0.098 | -0.040 | 0.019 |
| 57.12 | -0.017 | 0.042 | 0.101 | 0.030 | -0.098 | -0.039 | 0.021 |
| 57.33 | -0.017 | 0.041 | 0.099 | 0.031 | -0.098 | -0.038 | 0.022 |
| 57.55 | -0.016 | 0.040 | 0.097 | 0.031 | -0.098 | -0.037 | 0.023 |
| 57.76 | -0.015 | 0.040 | 0.095 | 0.031 | -0.098 | -0.037 | 0.025 |
| 57.97 | -0.014 | 0.039 | 0.093 | 0.032 | -0.098 | -0.036 | 0.026 |
| 58.18 | -0.014 | 0.039 | 0.092 | 0.032 | -0.098 | -0.035 | 0.027 |
| 58.39 | -0.013 | 0.039 | 0.091 | 0.032 | -0.098 | -0.035 | 0.028 |
| 58.61 | -0.012 | 0.039 | 0.090 | 0.032 | -0.098 | -0.035 | 0.029 |
| 58.82 | -0.012 | 0.039 | 0.090 | 0.033 | -0.098 | -0.034 | 0.030 |
| 59.03 | -0.011 | 0.039 | 0.090 | 0.033 | -0.099 | -0.034 | 0.031 |
| 59.24 | -0.010 | 0.039 | 0.089 | 0.033 | -0.099 | -0.034 | 0.031 |
| 59.45 | -0.010 | 0.040 | 0.090 | 0.033 | -0.099 | -0.034 | 0.032 |
| 59.67 | -0.009 | 0.040 | 0.090 | 0.033 | -0.099 | -0.034 | 0.032 |
| 59.88 | -0.008 | 0.041 | 0.090 | 0.034 | -0.099 | -0.034 | 0.032 |
| 60.09 | -0.008 | 0.042 | 0.091 | 0.034 | -0.100 | -0.034 | 0.032 |
| 60.30 | -0.007 | 0.042 | 0.092 | 0.034 | -0.100 | -0.034 | 0.032 |
| 60.52 | -0.006 | 0.043 | 0.092 | 0.034 | -0.100 | -0.034 | 0.032 |
| 60.73 | -0.005 | 0.044 | 0.093 | 0.034 | -0.100 | -0.034 | 0.032 |
| 60.94 | -0.004 | 0.045 | 0.095 | 0.034 | -0.101 | -0.034 | 0.032 |
| 61.15 | -0.003 | 0.046 | 0.096 | 0.034 | -0.101 | -0.035 | 0.031 |
| 61.36 | -0.002 | 0.047 | 0.097 | 0.034 | -0.101 | -0.035 | 0.031 |
| 61.58 | -0.002 | 0.049 | 0.099 | 0.034 | -0.101 | -0.035 | 0.031 |
| 61.79 | -0.001 | 0.050 | 0.100 | 0.034 | -0.102 | -0.036 | 0.030 |
| 62.00 | 0.000 | 0.051 | 0.102 | 0.034 | -0.102 | -0.036 | 0.030 |
| 62.21 | 0.001 | 0.052 | 0.103 | 0.034 | -0.103 | -0.037 | 0.029 |
| 62.42 | 0.002 | 0.054 | 0.105 | 0.034 | -0.103 | -0.037 | 0.029 |
| 62.64 | 0.003 | 0.055 | 0.107 | 0.034 | -0.104 | -0.038 | 0.028 |
| 62.85 | 0.004 | 0.057 | 0.109 | 0.034 | -0.105 | -0.039 | 0.027 |
| 63.06 | 0.005 | 0.058 | 0.111 | 0.034 | -0.105 | -0.039 | 0.027 |
| 63.27 | 0.007 | 0.060 | 0.113 | 0.034 | -0.106 | -0.040 | 0.026 |
| 63.48 | 0.008 | 0.061 | 0.115 | 0.034 | -0.107 | -0.041 | 0.026 |
| 63.70 | 0.009 | 0.063 | 0.117 | 0.034 | -0.107 | -0.041 | 0.025 |
| 63.91 | 0.010 | 0.065 | 0.120 | 0.034 | -0.108 | -0.042 | 0.024 |
| 64.12 | 0.011 | 0.066 | 0.122 | 0.034 | -0.109 | -0.043 | 0.024 |
| 64.33 | 0.011 | 0.068 | 0.124 | 0.034 | -0.110 | -0.043 | 0.023 |
| 64.55 | 0.012 | 0.070 | 0.127 | 0.034 | -0.111 | -0.044 | 0.022 |
| 64.76 | 0.013 | 0.071 | 0.129 | 0.034 | -0.111 | -0.045 | 0.022 |
| 64.97 | 0.014 | 0.073 | 0.131 | 0.034 | -0.112 | -0.046 | 0.021 |
| 65.18 | 0.015 | 0.074 | 0.134 | 0.034 | -0.113 | -0.046 | 0.020 |
| 65.39 | 0.016 | 0.076 | 0.136 | 0.034 | -0.114 | -0.047 | 0.019 |
| 65.61 | 0.017 | 0.078 | 0.139 | 0.034 | -0.115 | -0.048 | 0.019 |
| 65.82 | 0.018 | 0.079 | 0.141 | 0.034 | -0.116 | -0.049 | 0.018 |
| **66.03** | **0.019** | **0.081** | **0.143** | **0.034** | **-0.116** | **-0.049** | **0.017** |
| **66.24** | **0.019** | **0.083** | **0.146** | **0.034** | **-0.117** | **-0.050** | **0.017** |
| **66.45** | **0.020** | **0.084** | **0.148** | **0.034** | **-0.118** | **-0.051** | **0.016** |
| **66.67** | **0.021** | **0.086** | **0.150** | **0.034** | **-0.118** | **-0.051** | **0.016** |
| **66.88** | **0.022** | **0.087** | **0.153** | **0.034** | **-0.119** | **-0.052** | **0.015** |
| **67.09** | **0.022** | **0.089** | **0.155** | **0.034** | **-0.119** | **-0.053** | **0.014** |
| **67.30** | **0.023** | **0.090** | **0.157** | **0.034** | **-0.120** | **-0.053** | **0.014** |
| **67.52** | **0.024** | **0.091** | **0.159** | **0.034** | **-0.120** | **-0.054** | **0.013** |
| **67.73** | **0.024** | **0.093** | **0.161** | **0.034** | **-0.121** | **-0.054** | **0.013** |
| **67.94** | **0.025** | **0.094** | **0.163** | **0.034** | **-0.121** | **-0.054** | **0.012** |
| **68.15** | **0.025** | **0.095** | **0.165** | **0.034** | **-0.121** | **-0.055** | **0.012** |
| **68.36** | **0.026** | **0.096** | **0.167** | **0.034** | **-0.121** | **-0.055** | **0.012** |
| **68.58** | **0.026** | **0.097** | **0.168** | **0.034** | **-0.121** | **-0.055** | **0.011** |
| **68.79** | **0.027** | **0.098** | **0.170** | **0.034** | **-0.121** | **-0.055** | **0.011** |
| **69.00** | **0.027** | **0.099** | **0.171** | **0.034** | **-0.121** | **-0.055** | **0.011** |
| **69.21** | **0.028** | **0.100** | **0.172** | **0.034** | **-0.121** | **-0.055** | **0.010** |
| **69.42** | **0.028** | **0.101** | **0.173** | **0.033** | **-0.121** | **-0.055** | **0.010** |
| **69.64** | **0.029** | **0.101** | **0.174** | **0.033** | **-0.120** | **-0.055** | **0.010** |
| **69.85** | **0.029** | **0.102** | **0.175** | **0.033** | **-0.120** | **-0.055** | **0.010** |
| **70.06** | **0.029** | **0.103** | **0.176** | **0.033** | **-0.119** | **-0.054** | **0.010** |
| **70.27** | **0.030** | **0.103** | **0.176** | **0.033** | **-0.118** | **-0.054** | **0.010** |
| **70.48** | **0.030** | **0.103** | **0.176** | **0.033** | **-0.118** | **-0.054** | **0.010** |
| **70.70** | **0.030** | **0.103** | **0.176** | **0.032** | **-0.117** | **-0.053** | **0.010** |
| **70.91** | **0.031** | **0.104** | **0.176** | **0.032** | **-0.116** | **-0.053** | **0.010** |
| **71.12** | **0.031** | **0.103** | **0.176** | **0.032** | **-0.114** | **-0.052** | **0.011** |
| **71.33** | **0.031** | **0.103** | **0.176** | **0.032** | **-0.113** | **-0.051** | **0.011** |
| **71.55** | **0.031** | **0.103** | **0.175** | **0.031** | **-0.112** | **-0.050** | **0.011** |
| **71.76** | **0.032** | **0.103** | **0.174** | **0.031** | **-0.110** | **-0.049** | **0.012** |
| **71.97** | **0.032** | **0.102** | **0.173** | **0.031** | **-0.109** | **-0.048** | **0.012** |
| **72.18** | **0.032** | **0.102** | **0.171** | **0.030** | **-0.107** | **-0.047** | **0.013** |
| **72.39** | **0.032** | **0.101** | **0.169** | **0.030** | **-0.105** | **-0.046** | **0.013** |
| **72.61** | **0.032** | **0.100** | **0.168** | **0.030** | **-0.103** | **-0.045** | **0.014** |
| **72.82** | **0.032** | **0.099** | **0.165** | **0.029** | **-0.101** | **-0.043** | **0.014** |
| **73.03** | **0.032** | **0.098** | **0.163** | **0.029** | **-0.099** | **-0.042** | **0.015** |
| **73.24** | **0.032** | **0.096** | **0.160** | **0.029** | **-0.097** | **-0.040** | **0.016** |
| **73.45** | **0.032** | **0.095** | **0.157** | **0.028** | **-0.094** | **-0.039** | **0.017** |
| **73.67** | **0.032** | **0.093** | **0.154** | **0.028** | **-0.092** | **-0.037** | **0.018** |
| **73.88** | **0.031** | **0.091** | **0.151** | **0.027** | **-0.089** | **-0.035** | **0.019** |
| **74.09** | **0.031** | **0.089** | **0.147** | **0.027** | **-0.086** | **-0.033** | **0.020** |
| **74.30** | **0.031** | **0.087** | **0.143** | **0.027** | **-0.083** | **-0.031** | **0.021** |
| **74.52** | **0.030** | **0.085** | **0.139** | **0.026** | **-0.080** | **-0.029** | **0.023** |
| **74.73** | **0.029** | **0.082** | **0.135** | **0.026** | **-0.078** | **-0.027** | **0.024** |
| **74.94** | **0.028** | **0.079** | **0.130** | **0.026** | **-0.075** | **-0.024** | **0.026** |
| 75.15 | 0.027 | 0.076 | 0.125 | 0.025 | -0.072 | -0.022 | 0.028 |
| 75.36 | 0.026 | 0.073 | 0.121 | 0.025 | -0.069 | -0.019 | 0.030 |
| 75.58 | 0.024 | 0.070 | 0.116 | 0.025 | -0.066 | -0.016 | 0.033 |
| 75.79 | 0.022 | 0.067 | 0.111 | 0.025 | -0.063 | -0.014 | 0.036 |
| 76.00 | 0.020 | 0.063 | 0.106 | 0.025 | -0.061 | -0.011 | 0.039 |

*Note.* Coefficients (*β*) represent the estimated magnitude of the associations between changes in DMN FC and changes in letter comparison performance for each group. The age range highlighted in bold indicates where significant differences between the two groups were found. Abbreviations: DMN = Default mode network, SE = Standard error, CI = Confidence interval.

**Supplemental Table 4.3.a.** Age-varying relationships for change in SN FC with change in DSST performance.

| Age | ModVig_L  (Lower 95% CI) | Coefficients (*β*)  in ModVig | ModVig_U  (Upper 95% CI) | SE of  Association | Light_L  (Lower 95% CI) | Coefficients (*β*)  in Light | Light_U  (Upper 95% CI) |
| --- | --- | --- | --- | --- | --- | --- | --- |
| 55.00 | -0.039 | 0.071 | 0.182 | 0.051 | -0.221 | -0.121 | -0.021 |
| 55.21 | -0.036 | 0.071 | 0.177 | 0.050 | -0.215 | -0.118 | -0.021 |
| 55.42 | -0.033 | 0.071 | 0.174 | 0.049 | -0.211 | -0.116 | -0.021 |
| 55.64 | -0.030 | 0.070 | 0.170 | 0.048 | -0.207 | -0.113 | -0.020 |
| 55.85 | -0.027 | 0.070 | 0.167 | 0.047 | -0.203 | -0.111 | -0.019 |
| 56.06 | -0.025 | 0.070 | 0.165 | 0.047 | -0.200 | -0.109 | -0.018 |
| 56.27 | -0.022 | 0.070 | 0.162 | 0.046 | -0.198 | -0.107 | -0.016 |
| 56.48 | -0.020 | 0.070 | 0.160 | 0.046 | -0.195 | -0.105 | -0.014 |
| 56.70 | -0.018 | 0.070 | 0.159 | 0.046 | -0.193 | -0.103 | -0.013 |
| 56.91 | -0.016 | 0.071 | 0.157 | 0.046 | -0.191 | -0.101 | -0.011 |
| 57.12 | -0.014 | 0.071 | 0.156 | 0.046 | -0.190 | -0.099 | -0.009 |
| 57.33 | -0.012 | 0.071 | 0.155 | 0.046 | -0.188 | -0.097 | -0.007 |
| 57.55 | -0.011 | 0.072 | 0.154 | 0.047 | -0.187 | -0.096 | -0.005 |
| 57.76 | -0.009 | 0.072 | 0.154 | 0.047 | -0.186 | -0.094 | -0.003 |
| 57.97 | -0.007 | 0.073 | 0.153 | 0.047 | -0.185 | -0.093 | -0.001 |
| 58.18 | -0.006 | 0.074 | 0.153 | 0.047 | -0.184 | -0.091 | 0.001 |
| 58.39 | -0.004 | 0.074 | 0.153 | 0.047 | -0.183 | -0.090 | 0.003 |
| 58.61 | -0.003 | 0.075 | 0.153 | 0.048 | -0.182 | -0.088 | 0.005 |
| 58.82 | -0.002 | 0.076 | 0.154 | 0.048 | -0.181 | -0.087 | 0.007 |
| 59.03 | 0.000 | 0.077 | 0.154 | 0.048 | -0.180 | -0.086 | 0.008 |
| 59.24 | 0.001 | 0.078 | 0.155 | 0.048 | -0.179 | -0.085 | 0.010 |
| 59.45 | 0.003 | 0.079 | 0.155 | 0.048 | -0.178 | -0.084 | 0.011 |
| 59.67 | 0.004 | 0.080 | 0.156 | 0.048 | -0.177 | -0.082 | 0.012 |
| 59.88 | 0.006 | 0.081 | 0.157 | 0.048 | -0.176 | -0.081 | 0.013 |
| 60.09 | 0.007 | 0.082 | 0.158 | 0.048 | -0.175 | -0.081 | 0.014 |
| 60.30 | 0.009 | 0.084 | 0.159 | 0.048 | -0.175 | -0.080 | 0.015 |
| 60.52 | 0.010 | 0.085 | 0.160 | 0.048 | -0.174 | -0.079 | 0.016 |
| 60.73 | 0.012 | 0.086 | 0.161 | 0.048 | -0.173 | -0.078 | 0.017 |
| 60.94 | 0.013 | 0.088 | 0.162 | 0.048 | -0.172 | -0.077 | 0.018 |
| 61.15 | 0.015 | 0.089 | 0.163 | 0.048 | -0.171 | -0.076 | 0.018 |
| 61.36 | 0.016 | 0.090 | 0.164 | 0.048 | -0.170 | -0.076 | 0.019 |
| 61.58 | 0.018 | 0.092 | 0.166 | 0.048 | -0.169 | -0.075 | 0.019 |
| 61.79 | 0.019 | 0.093 | 0.167 | 0.048 | -0.169 | -0.074 | 0.020 |
| **62.00** | **0.021** | **0.095** | **0.169** | **0.048** | **-0.168** | **-0.074** | **0.020** |
| **62.21** | **0.022** | **0.096** | **0.170** | **0.048** | **-0.167** | **-0.073** | **0.021** |
| **62.42** | **0.024** | **0.098** | **0.171** | **0.048** | **-0.167** | **-0.073** | **0.021** |
| **62.64** | **0.025** | **0.099** | **0.173** | **0.048** | **-0.166** | **-0.072** | **0.021** |
| **62.85** | **0.027** | **0.101** | **0.175** | **0.048** | **-0.165** | **-0.072** | **0.022** |
| **63.06** | **0.028** | **0.102** | **0.176** | **0.048** | **-0.165** | **-0.071** | **0.022** |
| **63.27** | **0.029** | **0.104** | **0.178** | **0.048** | **-0.164** | **-0.071** | **0.022** |
| **63.48** | **0.031** | **0.105** | **0.179** | **0.048** | **-0.164** | **-0.071** | **0.023** |
| **63.70** | **0.032** | **0.107** | **0.181** | **0.048** | **-0.164** | **-0.070** | **0.023** |
| **63.91** | **0.033** | **0.108** | **0.183** | **0.048** | **-0.163** | **-0.070** | **0.024** |
| **64.12** | **0.035** | **0.110** | **0.185** | **0.048** | **-0.163** | **-0.070** | **0.024** |
| **64.33** | **0.036** | **0.111** | **0.186** | **0.048** | **-0.163** | **-0.069** | **0.024** |
| **64.55** | **0.037** | **0.113** | **0.188** | **0.048** | **-0.163** | **-0.069** | **0.025** |
| **64.76** | **0.038** | **0.114** | **0.190** | **0.048** | **-0.163** | **-0.069** | **0.026** |
| **64.97** | **0.039** | **0.116** | **0.192** | **0.048** | **-0.163** | **-0.069** | **0.026** |
| **65.18** | **0.040** | **0.117** | **0.194** | **0.048** | **-0.163** | **-0.068** | **0.027** |
| **65.39** | **0.041** | **0.118** | **0.196** | **0.049** | **-0.163** | **-0.068** | **0.027** |
| **65.61** | **0.042** | **0.120** | **0.197** | **0.049** | **-0.164** | **-0.068** | **0.028** |
| **65.82** | **0.043** | **0.121** | **0.199** | **0.049** | **-0.164** | **-0.068** | **0.029** |
| **66.03** | **0.044** | **0.123** | **0.201** | **0.049** | **-0.164** | **-0.067** | **0.029** |
| **66.24** | **0.045** | **0.124** | **0.203** | **0.050** | **-0.165** | **-0.067** | **0.030** |
| **66.45** | **0.046** | **0.125** | **0.205** | **0.050** | **-0.165** | **-0.067** | **0.031** |
| **66.67** | **0.046** | **0.126** | **0.207** | **0.050** | **-0.165** | **-0.067** | **0.032** |
| **66.88** | **0.047** | **0.128** | **0.208** | **0.051** | **-0.166** | **-0.067** | **0.033** |
| **67.09** | **0.048** | **0.129** | **0.210** | **0.051** | **-0.166** | **-0.066** | **0.033** |
| **67.30** | **0.048** | **0.130** | **0.212** | **0.051** | **-0.167** | **-0.066** | **0.034** |
| **67.52** | **0.049** | **0.131** | **0.213** | **0.052** | **-0.167** | **-0.066** | **0.035** |
| **67.73** | **0.049** | **0.132** | **0.215** | **0.052** | **-0.168** | **-0.066** | **0.036** |
| **67.94** | **0.049** | **0.133** | **0.217** | **0.052** | **-0.168** | **-0.066** | **0.037** |
| **68.15** | **0.050** | **0.134** | **0.218** | **0.053** | **-0.169** | **-0.065** | **0.038** |
| **68.36** | **0.050** | **0.135** | **0.219** | **0.053** | **-0.169** | **-0.065** | **0.039** |
| **68.58** | **0.050** | **0.136** | **0.221** | **0.053** | **-0.169** | **-0.065** | **0.040** |
| **68.79** | **0.051** | **0.136** | **0.222** | **0.054** | **-0.170** | **-0.065** | **0.041** |
| **69.00** | **0.051** | **0.137** | **0.223** | **0.054** | **-0.170** | **-0.064** | **0.041** |
| **69.21** | **0.051** | **0.138** | **0.224** | **0.054** | **-0.170** | **-0.064** | **0.042** |
| **69.42** | **0.051** | **0.138** | **0.225** | **0.055** | **-0.171** | **-0.064** | **0.043** |
| **69.64** | **0.051** | **0.139** | **0.226** | **0.055** | **-0.171** | **-0.063** | **0.044** |
| **69.85** | **0.051** | **0.139** | **0.227** | **0.055** | **-0.171** | **-0.063** | **0.045** |
| **70.06** | **0.051** | **0.139** | **0.227** | **0.056** | **-0.171** | **-0.063** | **0.046** |
| **70.27** | **0.052** | **0.140** | **0.228** | **0.056** | **-0.172** | **-0.062** | **0.047** |
| **70.48** | **0.052** | **0.140** | **0.228** | **0.056** | **-0.172** | **-0.062** | **0.048** |
| **70.70** | **0.051** | **0.140** | **0.228** | **0.057** | **-0.172** | **-0.061** | **0.050** |
| 70.91 | 0.051 | 0.140 | 0.228 | 0.057 | -0.172 | -0.061 | 0.051 |
| 71.12 | 0.051 | 0.140 | 0.228 | 0.057 | -0.173 | -0.060 | 0.052 |
| 71.33 | 0.051 | 0.139 | 0.228 | 0.058 | -0.173 | -0.060 | 0.054 |
| 71.55 | 0.051 | 0.139 | 0.227 | 0.058 | -0.174 | -0.059 | 0.055 |
| 71.76 | 0.051 | 0.139 | 0.227 | 0.059 | -0.174 | -0.058 | 0.057 |
| 71.97 | 0.051 | 0.138 | 0.226 | 0.060 | -0.175 | -0.058 | 0.059 |
| 72.18 | 0.050 | 0.138 | 0.225 | 0.060 | -0.176 | -0.057 | 0.061 |
| 72.39 | 0.050 | 0.137 | 0.224 | 0.061 | -0.177 | -0.056 | 0.064 |
| 72.61 | 0.050 | 0.136 | 0.222 | 0.062 | -0.178 | -0.055 | 0.067 |
| 72.82 | 0.049 | 0.135 | 0.221 | 0.064 | -0.179 | -0.055 | 0.070 |
| 73.03 | 0.049 | 0.134 | 0.219 | 0.065 | -0.181 | -0.054 | 0.073 |
| 73.24 | 0.048 | 0.133 | 0.218 | 0.066 | -0.183 | -0.053 | 0.077 |
| 73.45 | 0.047 | 0.132 | 0.216 | 0.068 | -0.185 | -0.052 | 0.082 |
| 73.67 | 0.046 | 0.130 | 0.214 | 0.070 | -0.188 | -0.051 | 0.086 |
| 73.88 | 0.045 | 0.129 | 0.212 | 0.072 | -0.191 | -0.050 | 0.091 |
| 74.09 | 0.044 | 0.127 | 0.210 | 0.074 | -0.194 | -0.049 | 0.097 |
| 74.30 | 0.043 | 0.125 | 0.207 | 0.077 | -0.198 | -0.047 | 0.103 |
| 74.52 | 0.042 | 0.123 | 0.205 | 0.080 | -0.202 | -0.046 | 0.110 |
| 74.73 | 0.040 | 0.121 | 0.202 | 0.083 | -0.207 | -0.045 | 0.118 |
| 74.94 | 0.038 | 0.119 | 0.200 | 0.086 | -0.212 | -0.043 | 0.126 |
| 75.15 | 0.036 | 0.117 | 0.197 | 0.090 | -0.218 | -0.042 | 0.134 |
| 75.36 | 0.033 | 0.114 | 0.195 | 0.094 | -0.224 | -0.040 | 0.144 |
| 75.58 | 0.031 | 0.112 | 0.192 | 0.098 | -0.231 | -0.039 | 0.153 |
| 75.79 | 0.027 | 0.109 | 0.190 | 0.103 | -0.238 | -0.037 | 0.164 |
| 76.00 | 0.024 | 0.106 | 0.188 | 0.107 | -0.246 | -0.036 | 0.175 |

*Note.* Coefficients (*β*) represent the estimated magnitude of the associations between changes in SN FC and changes in DSST performance for each group. The age range highlighted in bold indicates where significant differences between the two groups were found. Abbreviations: SN = Salience network, DSST = Digit symbol substitution test, SE = Standard error, CI = Confidence interval.

**Supplemental Table 4.3.b.** Age-varying relationships for change in SN FC with change in Pattern Comparison performance.

| Age | ModVig_L  (Lower 95% CI) | Coefficients (*β*)  in ModVig | ModVig_U  (Upper 95% CI) | SE of  Association | Light_L  (Lower 95% CI) | Coefficients (*β*)  in Light | Light_U  (Upper 95% CI) |
| --- | --- | --- | --- | --- | --- | --- | --- |
| 55.00 | -0.024 | 0.079 | 0.183 | 0.036 | -0.161 | -0.090 | -0.018 |
| 55.21 | -0.022 | 0.078 | 0.178 | 0.036 | -0.156 | -0.085 | -0.013 |
| 55.42 | -0.019 | 0.077 | 0.174 | 0.036 | -0.151 | -0.080 | -0.008 |
| 55.64 | -0.017 | 0.076 | 0.170 | 0.037 | -0.147 | -0.075 | -0.003 |
| 55.85 | -0.015 | 0.076 | 0.166 | 0.037 | -0.144 | -0.071 | 0.001 |
| 56.06 | -0.013 | 0.075 | 0.163 | 0.037 | -0.140 | -0.067 | 0.006 |
| 56.27 | -0.011 | 0.074 | 0.160 | 0.038 | -0.138 | -0.063 | 0.011 |
| 56.48 | -0.009 | 0.074 | 0.157 | 0.038 | -0.135 | -0.060 | 0.016 |
| 56.70 | -0.008 | 0.074 | 0.155 | 0.039 | -0.133 | -0.056 | 0.020 |
| 56.91 | -0.006 | 0.073 | 0.153 | 0.040 | -0.131 | -0.053 | 0.024 |
| 57.12 | -0.005 | 0.073 | 0.151 | 0.040 | -0.129 | -0.050 | 0.028 |
| 57.33 | -0.003 | 0.073 | 0.150 | 0.041 | -0.128 | -0.048 | 0.032 |
| 57.55 | -0.002 | 0.074 | 0.149 | 0.041 | -0.126 | -0.045 | 0.036 |
| 57.76 | 0.000 | 0.074 | 0.148 | 0.042 | -0.125 | -0.043 | 0.039 |
| 57.97 | 0.001 | 0.074 | 0.147 | 0.043 | -0.124 | -0.041 | 0.042 |
| 58.18 | 0.003 | 0.074 | 0.146 | 0.043 | -0.124 | -0.039 | 0.045 |
| 58.39 | 0.004 | 0.075 | 0.146 | 0.044 | -0.123 | -0.038 | 0.048 |
| 58.61 | 0.005 | 0.076 | 0.146 | 0.044 | -0.123 | -0.036 | 0.051 |
| 58.82 | 0.007 | 0.076 | 0.146 | 0.045 | -0.122 | -0.035 | 0.053 |
| 59.03 | 0.008 | 0.077 | 0.146 | 0.045 | -0.122 | -0.034 | 0.055 |
| 59.24 | 0.010 | 0.078 | 0.146 | 0.046 | -0.122 | -0.033 | 0.057 |
| 59.45 | 0.011 | 0.079 | 0.146 | 0.046 | -0.122 | -0.032 | 0.059 |
| 59.67 | 0.012 | 0.080 | 0.147 | 0.047 | -0.122 | -0.031 | 0.060 |
| 59.88 | 0.014 | 0.081 | 0.147 | 0.047 | -0.123 | -0.031 | 0.061 |
| 60.09 | 0.015 | 0.082 | 0.148 | 0.047 | -0.123 | -0.030 | 0.063 |
| 60.30 | 0.017 | 0.083 | 0.149 | 0.048 | -0.123 | -0.030 | 0.063 |
| 60.52 | 0.018 | 0.084 | 0.149 | 0.048 | -0.124 | -0.030 | 0.064 |
| 60.73 | 0.020 | 0.085 | 0.150 | 0.048 | -0.124 | -0.030 | 0.065 |
| 60.94 | 0.021 | 0.086 | 0.151 | 0.048 | -0.125 | -0.030 | 0.065 |
| 61.15 | 0.023 | 0.088 | 0.152 | 0.049 | -0.126 | -0.030 | 0.065 |
| 61.36 | 0.025 | 0.089 | 0.154 | 0.049 | -0.126 | -0.030 | 0.065 |
| 61.58 | 0.026 | 0.090 | 0.155 | 0.049 | -0.127 | -0.031 | 0.065 |
| 61.79 | 0.028 | 0.092 | 0.156 | 0.049 | -0.128 | -0.031 | 0.065 |
| 62.00 | 0.029 | 0.093 | 0.157 | 0.049 | -0.129 | -0.032 | 0.065 |
| 62.21 | 0.031 | 0.095 | 0.159 | 0.050 | -0.130 | -0.033 | 0.064 |
| 62.42 | 0.032 | 0.096 | 0.160 | 0.050 | -0.131 | -0.034 | 0.064 |
| 62.64 | 0.034 | 0.098 | 0.162 | 0.050 | -0.132 | -0.034 | 0.063 |
| 62.85 | 0.035 | 0.099 | 0.163 | 0.050 | -0.133 | -0.035 | 0.062 |
| 63.06 | 0.037 | 0.101 | 0.165 | 0.050 | -0.134 | -0.036 | 0.062 |
| 63.27 | 0.038 | 0.102 | 0.166 | 0.050 | -0.136 | -0.037 | 0.061 |
| 63.48 | 0.040 | 0.104 | 0.168 | 0.050 | -0.137 | -0.039 | 0.060 |
| 63.70 | 0.041 | 0.106 | 0.170 | 0.050 | -0.138 | -0.040 | 0.058 |
| 63.91 | 0.043 | 0.107 | 0.172 | 0.050 | -0.139 | -0.041 | 0.057 |
| 64.12 | 0.044 | 0.109 | 0.174 | 0.050 | -0.140 | -0.042 | 0.056 |
| 64.33 | 0.045 | 0.110 | 0.175 | 0.050 | -0.141 | -0.043 | 0.055 |
| 64.55 | 0.046 | 0.112 | 0.177 | 0.050 | -0.143 | -0.045 | 0.053 |
| 64.76 | 0.047 | 0.113 | 0.179 | 0.050 | -0.144 | -0.046 | 0.052 |
| 64.97 | 0.048 | 0.115 | 0.181 | 0.050 | -0.145 | -0.047 | 0.051 |
| 65.18 | 0.049 | 0.116 | 0.183 | 0.050 | -0.146 | -0.049 | 0.049 |
| **65.39** | **0.050** | **0.118** | **0.185** | **0.050** | **-0.147** | **-0.050** | **0.048** |
| **65.61** | **0.051** | **0.119** | **0.187** | **0.050** | **-0.149** | **-0.051** | **0.046** |
| **65.82** | **0.052** | **0.121** | **0.189** | **0.050** | **-0.150** | **-0.052** | **0.045** |
| **66.03** | **0.053** | **0.122** | **0.191** | **0.049** | **-0.151** | **-0.054** | **0.043** |
| **66.24** | **0.053** | **0.123** | **0.193** | **0.049** | **-0.152** | **-0.055** | **0.042** |
| **66.45** | **0.054** | **0.125** | **0.195** | **0.049** | **-0.153** | **-0.056** | **0.040** |
| **66.67** | **0.054** | **0.126** | **0.197** | **0.049** | **-0.154** | **-0.057** | **0.039** |
| **66.88** | **0.055** | **0.127** | **0.199** | **0.049** | **-0.154** | **-0.058** | **0.037** |
| **67.09** | **0.055** | **0.128** | **0.201** | **0.049** | **-0.155** | **-0.060** | **0.036** |
| **67.30** | **0.056** | **0.129** | **0.203** | **0.049** | **-0.156** | **-0.061** | **0.035** |
| **67.52** | **0.056** | **0.131** | **0.205** | **0.048** | **-0.157** | **-0.062** | **0.033** |
| **67.73** | **0.056** | **0.131** | **0.207** | **0.048** | **-0.157** | **-0.063** | **0.032** |
| **67.94** | **0.056** | **0.132** | **0.209** | **0.048** | **-0.157** | **-0.063** | **0.031** |
| **68.15** | **0.056** | **0.133** | **0.210** | **0.048** | **-0.158** | **-0.064** | **0.029** |
| **68.36** | **0.056** | **0.134** | **0.212** | **0.048** | **-0.158** | **-0.065** | **0.028** |
| **68.58** | **0.056** | **0.135** | **0.213** | **0.047** | **-0.158** | **-0.066** | **0.027** |
| **68.79** | **0.056** | **0.135** | **0.215** | **0.047** | **-0.158** | **-0.066** | **0.026** |
| **69.00** | **0.056** | **0.136** | **0.216** | **0.047** | **-0.158** | **-0.067** | **0.025** |
| **69.21** | **0.056** | **0.137** | **0.217** | **0.046** | **-0.158** | **-0.067** | **0.024** |
| **69.42** | **0.056** | **0.137** | **0.218** | **0.046** | **-0.157** | **-0.067** | **0.023** |
| **69.64** | **0.056** | **0.137** | **0.219** | **0.046** | **-0.157** | **-0.067** | **0.022** |
| **69.85** | **0.055** | **0.137** | **0.220** | **0.045** | **-0.156** | **-0.067** | **0.022** |
| **70.06** | **0.055** | **0.138** | **0.220** | **0.045** | **-0.155** | **-0.067** | **0.021** |
| **70.27** | **0.054** | **0.138** | **0.221** | **0.044** | **-0.154** | **-0.067** | **0.020** |
| **70.48** | **0.054** | **0.137** | **0.221** | **0.044** | **-0.153** | **-0.067** | **0.020** |
| **70.70** | **0.053** | **0.137** | **0.221** | **0.044** | **-0.151** | **-0.066** | **0.019** |
| **70.91** | **0.053** | **0.137** | **0.221** | **0.043** | **-0.150** | **-0.065** | **0.019** |
| **71.12** | **0.052** | **0.137** | **0.221** | **0.043** | **-0.148** | **-0.064** | **0.019** |
| **71.33** | **0.052** | **0.136** | **0.220** | **0.042** | **-0.146** | **-0.064** | **0.019** |
| **71.55** | **0.051** | **0.135** | **0.220** | **0.042** | **-0.144** | **-0.062** | **0.019** |
| **71.76** | **0.050** | **0.135** | **0.219** | **0.041** | **-0.141** | **-0.061** | **0.019** |
| **71.97** | **0.049** | **0.134** | **0.218** | **0.040** | **-0.139** | **-0.060** | **0.020** |
| **72.18** | **0.049** | **0.133** | **0.217** | **0.040** | **-0.136** | **-0.058** | **0.020** |
| **72.39** | **0.048** | **0.132** | **0.215** | **0.039** | **-0.133** | **-0.056** | **0.021** |
| **72.61** | **0.047** | **0.130** | **0.214** | **0.039** | **-0.130** | **-0.054** | **0.022** |
| **72.82** | **0.046** | **0.129** | **0.212** | **0.038** | **-0.126** | **-0.052** | **0.023** |
| **73.03** | **0.045** | **0.127** | **0.210** | **0.037** | **-0.123** | **-0.049** | **0.024** |
| **73.24** | **0.044** | **0.126** | **0.207** | **0.037** | **-0.119** | **-0.047** | **0.025** |
| **73.45** | **0.042** | **0.124** | **0.205** | **0.036** | **-0.115** | **-0.044** | **0.027** |
| **73.67** | **0.041** | **0.122** | **0.202** | **0.036** | **-0.110** | **-0.041** | **0.029** |
| **73.88** | **0.040** | **0.119** | **0.199** | **0.035** | **-0.106** | **-0.037** | **0.031** |
| **74.09** | **0.038** | **0.117** | **0.196** | **0.035** | **-0.102** | **-0.034** | **0.034** |
| 74.30 | 0.037 | 0.115 | 0.193 | 0.034 | -0.097 | -0.030 | 0.037 |
| 74.52 | 0.035 | 0.112 | 0.189 | 0.034 | -0.092 | -0.026 | 0.040 |
| 74.73 | 0.033 | 0.109 | 0.185 | 0.033 | -0.087 | -0.022 | 0.044 |
| 74.94 | 0.031 | 0.106 | 0.181 | 0.033 | -0.083 | -0.017 | 0.048 |
| 75.15 | 0.029 | 0.103 | 0.177 | 0.033 | -0.078 | -0.013 | 0.053 |
| 75.36 | 0.026 | 0.100 | 0.173 | 0.033 | -0.073 | -0.008 | 0.058 |
| 75.58 | 0.023 | 0.096 | 0.169 | 0.034 | -0.068 | -0.002 | 0.064 |
| 75.79 | 0.020 | 0.092 | 0.164 | 0.034 | -0.063 | 0.003 | 0.070 |
| 76.00 | 0.017 | 0.088 | 0.160 | 0.035 | -0.058 | 0.009 | 0.077 |

*Note.* Coefficients (*β*) represent the estimated magnitude of the associations between changes in SN FC and changes in pattern comparison performance for each group. The age range highlighted in bold indicates where significant differences between the two groups were found. Abbreviations: SN = Salience network, SE = Standard error, CI = Confidence interval.
